# Phylogeny, historical biogeography and characters evolution of the drought resistant fern *Pyrrosia* Mirbel (Polypodiaceae) inferred from plastid and nuclear markers

**DOI:** 10.1038/s41598-017-12839-w

**Published:** 2017-10-06

**Authors:** Xueping Wei, Yaodong Qi, Xianchun Zhang, Li Luo, Hui Shang, Ran Wei, Haitao Liu, Bengang Zhang

**Affiliations:** 1 Key Laboratory of Bioactive Substances and Resources Utilization of Chinese Herbal Medicine, Ministry of Education, Institute of Medicinal Plant Development, Chinese Academy of Medical Sciences, Peking Union Medical College, Beijing, China; 2 State Key Laboratory of Systematic and Evolutionary Botany, Institute of Botany, Chinese Academy of Sciences, Beijing, 100093 China; 3 Shanghai Chenshan Plant Science Research Center, Chinese Academy of Sciences; Shanghai Chenshan Botanical Garden, Shanghai, 201602 China

## Abstract

*Pyrrosia* s.l. comprises ca. 60 species with a disjunct Africa/Asia and Australia distribution. The infrageneric classification of *Pyrrosia* s.l. is controversial based on the phylogenetic analyses of chloroplast markers and morphology. Based on the expanded taxon sampling of *Pyrrosia* s.l. (51 species), we investigated its phylogeny, biogeography, character evolution and environmental adaptation by employing five chloroplastid markers (*rbc*L, *mat*K, *psb*A-*trn*H, and *rps*4 + *rps*4-*trn*S) and one single (low)-copy nuclear gene, *LEAFY*. *Pyrrosia* s.l. was divided into six major clades and eight subclades. Reticulate evolution was revealed both among clades and among species in *Pyrrosia* s.l. Ancestral character state optimization revealed high levels of homoplastic evolution of the diagnostic characters in *Pyrrosia* s.l., while the crassulacean acid metabolism pathway seems to have an independent origin. Molecular dating and biogeographic diversification analyses suggested that *Pyrrosia* s.l. originated no later than the Oligocene and the main clades diversified during the Oligocene and Miocene, with southern Asia, the Indo-China Peninsula and southwestern and southern China as the most likely ancestral areas. Transoceanic long-distance dispersal, rather than vicariance, contributed to the intercontinental disjunction. Diversification scenarios of *Pyrrosia* s.l. under geological movements and climate fluctuation are also discussed.

## Introduction

*Pyrrosia* Mirbel (Polypodiaceae) is a terrestrial fern genus that constitutes subfamily Platycerioideae B.K. Nayar along with *Platycerium* Desv^1^. This genus contains ca. 51–100 species and is widely distributed in Asia, ranging from Australia and New Zealand to Siberia and from Africa to various south Pacific islands^2–7^. *Pyrrosia* is well circumscribed by stellate hairs and characteristic connective venation pattern, which are two key characters to understanding the evolution of Polypodiaceae^4,8–10^. Most species of *Pyrrosia* are drought tolerant, and five species have been reported to use crassulacean acid metabolism (CAM) pathway^11–15^. The occurrence of CAM is considered an effective adaptation to arid environments, although CAM in lycophytes and ferns is rare^16–18^.

The uniform appearance of species of *Pyrrosia* leads to difficulties in species-level classification. Several authors have conducted taxonomic revisions on regional or worldwide scales^2–4,6,8,19–26^. Giesenhagen^27^ was the first to systematically describe the morphology and classification of 50 species. Ching^26^ studied species from mainland Asia and neighboring islands and treated *Pyrrosia* as a natural genus; he transferred 49 species into *Pyrrosia* and described five new species of this genus. Shing^3^, Shing and Iwatsuki^2,20^ considered more than 100 species in *Pyrrosia* and recognized 64 species in Asia and the adjacent Oceania. Hovenkamp^4^ completed a monograph of *Pyrrosia* from a global perspective and recognized ca. 51 species with a wide species concept. Finally, it is widely accepted that *Pyrrosia* contains ca. 60 species^8^. However, the infrageneric classification of *Pyrrosia* is controversial (Supplementary Table S1). Nayar and Chandra divided 14 species from India into six groups^28^. Shing^3^ divided *Pyrrosia*, excluding *Drymoglossum* C. Presl and *Saxiglossum* C. Presl, into two subgenera: subg. *Pyrrosia* and subg. *Niphopsis*. subg. *Niphopsis* includes only *P. samarensis* and *P. angustata*, and subg. *Pyrrosia* was divided into two sections and five series. Hovenkamp recognized ten groups based on cladistics analyses using 24 morphological characters, while seven species were not included in any groups^4^. Furthermore, he considered five groups therein were not well-established monophyletic group due to the lack of autapomorphies^4^. This consideration maybe related to the absence of trait evaluation based on a robust phylogenetic tree. Nearly all the groups established by Hovenkamp are irreconcilable with those recognized in Shing’s classification, except that the P. angustata-group of Hovenkamp is equivalent to the subg. *Niphopsis* of Shing. Yang completed a taxonomic revision of *Pyrrosia*, excluding *Drymoglossum* C. Presl and *Saxiglossum* C. Presl, in China and proposed two subgenera and six sections^25^. Unlike Hovenkamp, Yang grouped *P. stigmosa*, which previously belonged to the P. costata-group, with sect. *Drakeanae*, which was equivalent to the P. sheareri-group of Hovenkamp based on the fronds shape and indumenta characters. Yang also grouped *P. subfurfuracea* and *P. calvata*, belonging to the P. sheareri-group of Hovenkamp, with sect. *Costatae*, which is equivalent to the P. costata-group, based on the scales-bearing types^25^. In addition, infrageneric classification of *Pyrrosia* was always closely linked to the recognition of the segregate genera *Drymoglossum* and *Saxiglossum* ^5,8,29–32^. *Drymoglossum* and *Saxiglossum* are currently accepted as members of *Pyrrosia*, which has been confirmed by phylogenetic work^33–35^.

Several molecular phylogenetic studies involved *Pyrrosia*, some of which revealed that *Pyrrosia* was monophyletic and *Platycerium* was a sister group^33,36–45^. In the most recently study, Vasques *et al*.^46^ established a subgeneric classification of *Pyrrosia* based on three chloroplast (cp) markers of 38 species. Six subgenera were proposed: subg. *Lune*, subg. *Neoniphopsis*, subg. *Niphobolus*, subg. *Niphopsis*, subg. *Pyrrosia* and subg. *Solis*. Testo and Sundue studied the evolution of ferns based on six chloroplast markers of a 4000-species dataset suggested that *Pyrrosia* was paraphyletic and that *P. liebuschii* (Hieron) Schelpe was nested in *Platycerium* ^47^. Zhou *et al*.^35^ segregated the P. africana*-*group as a new genus *Hovenkampia* Li Bing Zhang & X.M. Zhou, and recognized four clades in *Pyrrosia* based on five cp markers in the recently study. Zhao preliminarily studied the infrageneric relationship of *Pyrrosia* based on 26 species, mainly in Asia, by using one cpDNA, *rps*4*-trn*S, and one nrDNA, *LEAFY* ^48^. Four main clades were recognized in *Pyrrosia* by *rps*4-*trn*S and a potential hybrid origin of *P. piloselloides* was suggested. Nevertheless, the sampling is far from completion, and the P. africana-group was not included. Evidences from both single parent genetic chloroplast markers and parental genetic nuclear gene are urgent needed to test the monophyletic of *Pyrrosia* and further understand the phylogenetic relationship within *Pyrrosia* based on more taxa.

The integration of phylogenetics, historical biogeography, paleogeography and climatology has provided a new perspective to understand the origin and diversification of biotas, which are of great interest to evolutionary biologists^47,49–59^. *Pyrrosia* is exclusively distributed in paleotropic regions, while *Platycerium*, the mostly related group of *Pyrrosia*, is distributed in paleotropic regions and South America^4^. Based on morphological analyses, Hovenkamp^4^ proposed that *Pyrrosia* originated from Africa in the Jurassic and that the present distribution of the genus was reached via ‘rafting’ to the Indian subcontinent and Australia. Schneider *et al*.^56^ and Schuettpelz and Pryer^60^ dated the original time of *Pyrrosia* to the late Eocene (ca. 35 Ma) based on molecular dating, while Testo and Sundue^47^ established the original time of the main lineage of *Pyrrosia* in early Paleocene (ca. 63.4 Ma). Kreier and Schneider^43^ discussed the phylogeny and biogeography of *Platycerium* and reestablished two lineages (Africa + Madagascar + South America and Australia + Asia) in *Platycerium*. However, the ancestral distributions for the basal nodes are poorly resolved. Therefore, elucidating the historical biogeography of *Pyrrosia* is perplexing due to differences in the distribution and species diversity centre of *Pyrrosia* from those of *Platycerium*.

The present study, based on comprehensive taxa sampling, employs nucleotide sequences of five chloroplast DNA markers (*rbc*L, *mat*K, *psb*A-*trn*H, and *rps*4 + *rps*4-*trn*S) and one single (low)- copy nuclear gene, *LEAFY*, to reconstruct the phylogeny of *Pyrrosia* and to explore its historical biogeography. In addition, evolution of the morphological diagnostic characters and environmental adaptations related to drought resistance are investigated.

## Results

### Sequence characteristics

Five cpDNA gene regions—*mat*K, *rbc*L, *psb*A-*trn*H and *rps*4 + *rps*4-*trn*S— were amplified in all 109 *Pyrrosia* individuals and the related taxa in Polypodiaceae and Davalliaceae. The sequence characters and parsimony-informative sites for individual gene markers are summarized in Table 1. The combined data matrix of the cpDNA fragments included up to 3760 nucleotides, of which 1325 (32.58%) were variable and 1054 (28.03%) were potentially parsimony informative. A unique insertion/deletion (up to 169 bp at site 131 to 300 bp) was present in the *psb*A-*trn*H gene of *Pyrrosia*. Maximum parsimony (MP) analyses of the combinded cpDNA data set resulted in 14,809 equally most parsimonious trees with a length of 4,007 steps. The consistency and retention index were relatively high (CI = 0.60, RI = 0.86). The optimal maximum likelihood (ML) phylogram for the combinded cpDNA data was *-InL* = 26,674.4552.

**Table 1:** Descriptive statistics of analyzed DNA sequence used in the present study.

Gene	Length (bp)	Alignment length (bp)	Number of variable characters (%)	Number of parsimonious informative characters (%)
*mat*K	820	820	510 (61.20)	425 (51.83)
*rbc*L	1277	1277	306 (23.96	244 (1)
*rps*4 + *rps*4-*trn*S	927–987	1047	399 (38.11)	306 (29.23)
*psb*A-*trn*H	303–496	597	110 (18.43)	79 (13.23)
combined cpDNA	3353–3560	3760	1325 (32.58)	1054 (28.03)
*LEAFY*	955–1009	1081	606 (56.06)	515 (47.64)

Polymerase chain reaction (PCR) amplification of *LEAFY* was successfully performed in 81 individuals representing 39 species of *Pyrrosia* and the related taxa in Polypodiaceae and Davalliaceae. The amplified *LEAFY* gene fragment included intron 1, exon2, intron 2 and the flanking exon 1 and exon 3 sequences. The aligned data matrix had 1081 characters, including 606 variable characters and 515 potentially parsimony-informative characters (Table 1). The MP analyses were stopped with 13,200 equally most parsimonious trees of 1539 steps sampled (CI = 0.61, RI = 0.88). The optimal ML phylogram had an *–InL* value of 9,969.4164.

### Phylogenetic analyses

#### cpDNA

**Figure 1:**
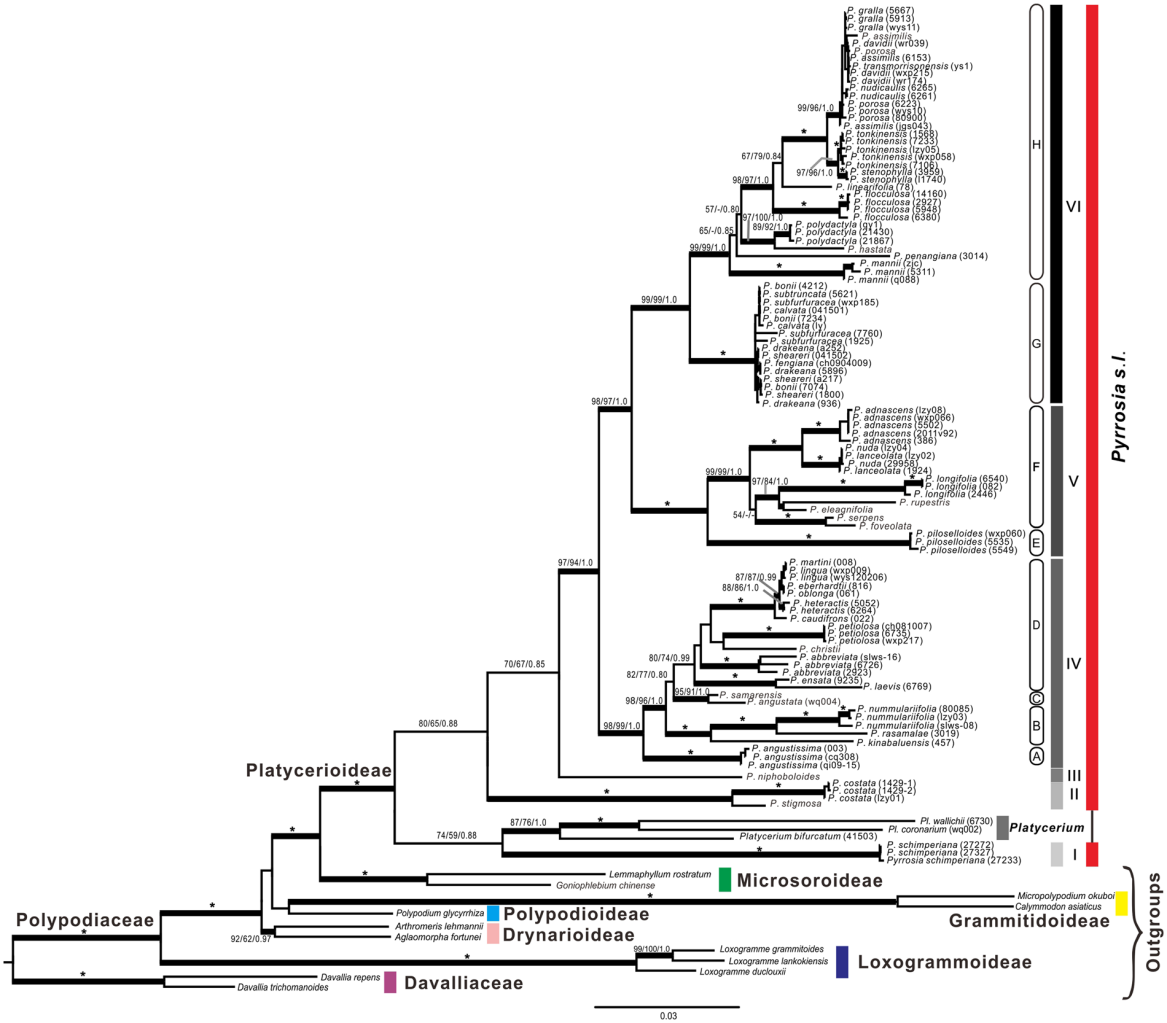
Phylogram of *Pyrrosia* s.l. obtained from the maximum likelihood (ML) analysis of the combined cpDNA data which contains all individuals of the investigated entities, including sequences of *rbc*L, *mat*K, *psb*A-*trn*H, and *rps*4 + *rps*4-*trn*S. Numbers on branches are support values (BS_ML_/BS_MP_/PP_BI_). Bold branches indicate BS_MP_, BS_ML_ ≥ 70% and PP_BI_ ≥ 0.95. Stars indicate BS_MP_, BS_ML_ = 100% and PP_BI_ = 1.0. Dash (−) indicates nodes with BS_MP_ or BS_ML_ < 50%.

The three phylogenetic analyses (MP, ML and Bayesian inference (BI)) revealed congruent topologies based on the combined data set of five cpDNA markers. Platycerioideae was recognized as a well-supported monophyly. The traditional *Pyrrosia* was paraphyletic, six main monophyletic clades (labelled as clades I–VI) were resolved (Fig. 1). Clade I clustered with *Platycerium* with weak supported, and this clade was the endemic African species, *P. schimperiana* (Mett. ex ﻿Kuhn) Alston (=*Hovenkampia schimperiana* (Mett. ex Kuhn) Li Bing Zhang & X. M. Zhou) which belonged to the P. africana-group of Hovenkamp^4^. Herein, we use *Pyrrosia* s.l. to represent *Pyrrosia* including P. africana-group, and *Pyrrosia* s.s. to represent *Pyrrosia* without P. africana-group. Clade II comprised *P. costata* and *P. stigmosa*, this clade corresponds to the P. costata-group described by Hovenkamp^4^, and it was resolved as the basal clade of *Pyrrosia* s.s. Clades III only contained *P. niphoboloides*. Clades IV to VI consisted of species distributed in Asia and Oceania, except the widespread *P. lanceolata*, which occupied Asia, Oceania and Africa. Clade IV contained four subclades (subclades A, B, C and D): *P. angustissima*, a species that previously belonged to the separated monotype genera *Saxiglossum*, was resolved as subclade A; *P*. *nummulariifolia*, *P*. *rasamalae* and *P*. *kinabaluensis* comprised subclade B with high support; subclade C consisted of species of the P. angustata-group; subclade D included the P. lingua-group and two undecided species, *P. laevis* and *P. ensata*. Clade V was strongly supported as the sister to clade VI, and both contained two subclades (subclades E and F; subclades G and H). Clade V contained *P. piloselloides* (subclade E) and species from the P. confluens-group, P. lanceolata-group and *P. foveolata* (subclade F). Clade VI was separated into two well-supported subclades: subclade G contained some of the species of the P. sheareri-group, and subclade H consisted of some of the species of the P. sheareri-group and species of P. porosa-group.

#### LEAFY

**Figure 2:**
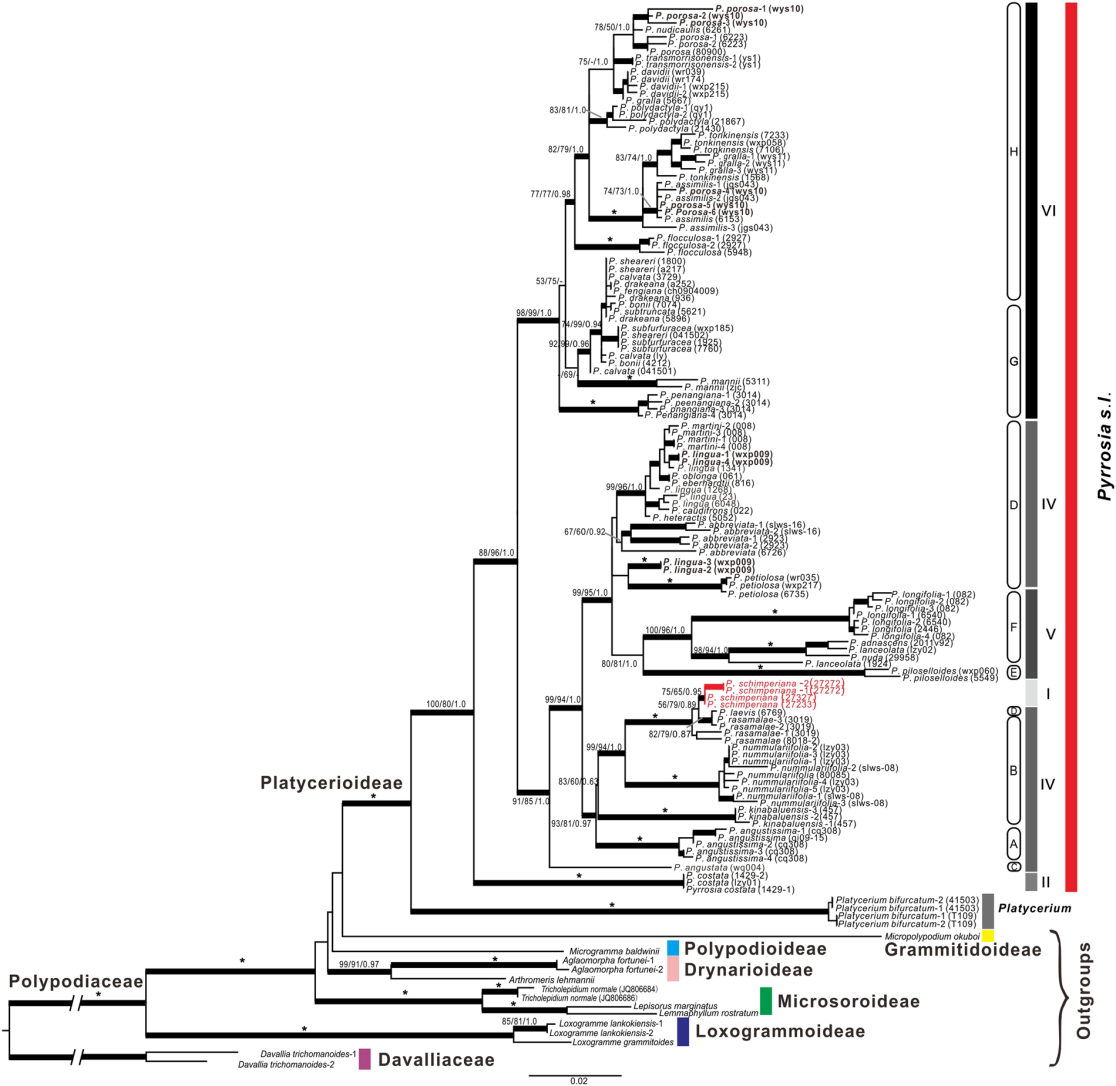
Phylogram of *Pyrrosia* s.l. obtained from the maximum likelihood (ML) analysis of *LEAFY*. Numbers on branches are support values (BS_ML_/BS_MP_/PP_BI_). Bold branches indicate BS_MP_, BS_ML_ ≥ 70% and PP_BI_ ≥ 0.95. Stars indicate BS_MP_, BS_ML_ = 100% and PP_BI_ = 1.0. Dash (−) indicates nodes with BS_MP_ or BS_ML_ < 50%. The crown branch length of Polypodiaceae and Davalliaceae were shortened as indicated by “//”.

Clade I, II, V and VI were recognized as monophyletic groups. Clade III was the basal clade of *Pyrrosia* s.l. Phylogenetic topologies of cpDNA and *LEAFY* showed significant conflicts of the relationship among clades I, IV and V, and subclades within clade IV (Fig. 2). (1) Clade I and V embed in clade IV, and resulted the monophyly clade IV in cpDNA trees splited into three parts in the tree generated from *LEAFY*: the first part, *P. angustata* (subclade C), was resolved as the basal group of clade IV; the second included subclades A, B and a species, *P. laevis*, which belonged to subclade D; the third included species from subclade D. (2) The phylogenetic position of *P. schimperiana* (clade I, = *H. schimperiana*, P. africana-group) was contradictory between datasets: *P. schimperiana* was resolved as an independent clade in the cpDNA trees (Fig. 1), while in the tree generated from *LEAFY*, *P. schimperiana* was nested in the second part of clade IV. (3) Clade V was the sister group of clade VI in the cpDNA trees, while it nested into clade IV and clustered with subclade D in the phylogenetic tree of *LEAFY*.

Furthermore, an individual of *P. lingua* had two sequence types of *LEAFY*: the sequences of one type clustered with other samples of *P. lingua*, and the sequences of another one clustered with *P. petiolosa*. Similarly, one individual of *P. porosa* also had two sequences types, one type clustered with *P. nudicaulis*, and the sequences of another type nested with *P. assimilis*.

### Molecular dating

**Figure 3:**
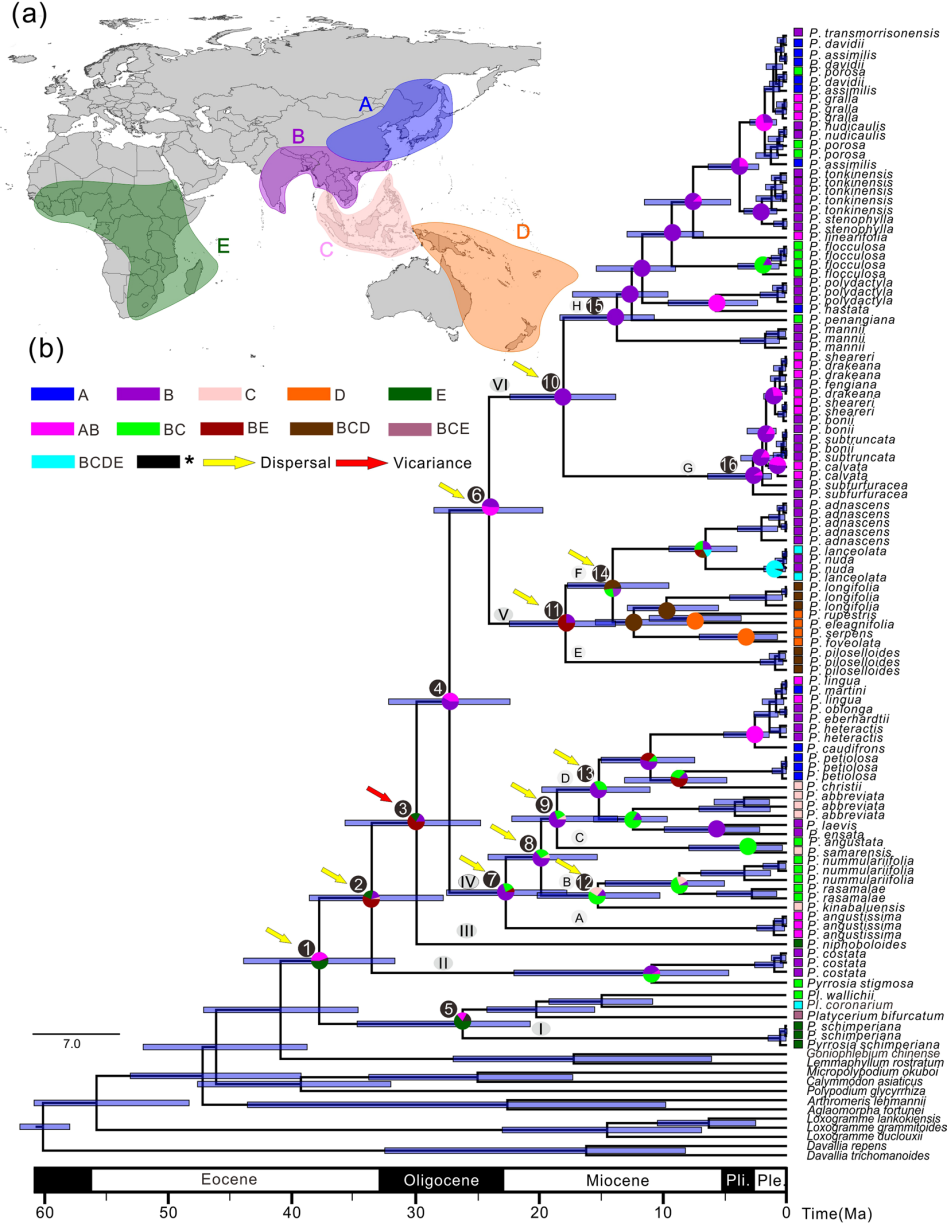
Global biogeographical patterns of *Pyrrosia* s.l. (**a**) Map showing five biogeographical areas in colors as defined in this study. (**b**) Schematic chronograms (maximum clade credibility topology) based on cpDNA data using uniform priors obtained from BEAST. The geological time scale (60 Ma–present) is shown at the bottom. Node numbers and mean ages refer to Table 2. Blue bars represent 95% highest posterior density of node age. Color-coded pie diagrams represent the probabilities of different states of ancestral area reconstruction (AAR) based on the dispersal–extinction–cladogenesis (DEC) model in RASP. Arrowheads represent the possible split events inferred in RASP. Biogeographical area abbreviations: A, eastern Asia (including central, eastern and northeastern China, Korea Peninsula, southern Japan and Far East); B, southern Asia, the Indo-China Peninsula and southwestern and southern China; C, Malesia (including Malaysia, Indonesia, and the Philippines); D, Australasia (including Australia, New Guinea, New Zealand, and the South Pacific islands); E, Africa (including Madagascar). Geological epoch abbreviations: Pli., Pliocene; Ple., Pleistocene. The spatial data of Fig. 3a was freely downloaded from http://www.diva-gis.org/Data, the base map was generated by ArcGIS v.9.3 (http://www.esri.com/software/arcgis/arcgis-for-desktop), and Fig. 3a was drawn by CorelDRAW v. x 8 (http://www.coreldraw.com/en/product/graphic-design-software/).

The mean ages and 95% highest posterior density (HPD) intervals of all labelled nodes within Platycerioideae were indicated in the chronogram (Fig. 3b) and in Table 2. The crown age of Platycerioideae was in the late Eocene (node 1, 37.98 Ma, HPD = 25.75–46.71 Ma). Clade I was separated from *Platycerium* at ca. 26.33 Ma (node 5, HPD = 16.78–39.39). The estimated age of the *Pyrrosia* s.s. (clades II–VI) was approximately the boundary of the Eocene and Oligocene (node 2, 33.71 Ma, HPD = 22.64–42.43). The most recent common ancestor (MRCA) of the main Asian and Oceanian species was dated backed 30.07 Ma (node 3, HPD = 20.80–38.75), Clade IV separated from clades V and VI at ca. 27.40 Ma (node 4, HPD = 19.56–35.90), and the latter two clades separated at ca. 24.19 Ma (node 6, HPD = 16.71–31.16). The crown node of clade IV was dated back to 22.82 Ma (node 7, HPD = 14.06–30.73). Estimated crown ages for clades V and VI are very similar, at 17.98 Ma and 18.14 Ma, respectively (nodes 11 and 10, Fig. 3b and Table 2).

**Table 2:** Mean age and 95% highest posterior density (HPD) values of each node.

Node	Estimated divergence time		Ancestral area
	Mean (Ma)	95% HPD (Ma)	DEC model (Akaike weights)
1	37.98	25.75–46.71	E (0.52)
2	33.71	22.64–42.43	BE (0.57)
3	30.07	20.80–38.75	BE (0.67)
4	27.40	19.56–35.90	B (0.69)
5	26.33	16.78–39.39	E (0.78)
6	24.19	16.71–31.16	B (0.65)
7	22.82	14.06–30.73	B (0.47)
8	19.96	13.09–28.57	B (0.59)
9	18.67	11.95–27.36	B (0.61)
10	18.14	12.36–27.31	B (1.00)
11	17.98	12.31–24.19	BE (0.75)
12	15.35	8.50–21.95	BC (0.55)
13	15.27	9.44–23.36	B (0.59)
14	14.15	8.34–20.15	B (0.39)
15	13.80	10.04–21.79	B (1.00)
16	2.76	1.22–12.22	B (0.87)

Ancestral areas with Akaike weight revealed by the dispersal–extinction–cladogenesis (DEC) model in RASP. Node numbers and area abbreviations refer to Fig. 3.

### Ancestral area reconstruction

The ancestral distributions obtained from the dispersal-extinction-cladogenesis (DEC) model for the major clades are shown in Fig. 3b and Table 2. The DEC model suggested that Platycerioideae most likely originated in areas E (Fig. 3b, node 1). The origin for the MRCA of *Pyrrosia* s.s. was unclear; two areas including southern Asia, Indo-China Peninsula and southwestern and southern China (area B), and Africa (area E), were all the supposed origin. The MRCA of the most Asian and Oceanian species originated in areas B (node 4). Clade IV may have originated in areas B and expanded towards Eastern Asia (area A) and Malesia (area C). The sister clades V and VI may have originated in area B and then expanded towards Eastern Asia (area A) and Australasia (area D), respectively. Clade I and *Platycerium* were suggested that originated in areas E. Several historical dispersal events were inferred, including the dispersal from area B to A (26.5 times), C (26.5 times), D (17.5 times) and E (17 time), and from area E to C (4.5 times), B (4 times), A (0.5 time), D (0.5 time) and D to B (3 time), C (3 times). The split of *Pyrrosia* s.s. from clade I and *Platycerium* and the split of clades II, V and VI, the diversification of the crown group of clade VI, V and VI and the infraspecific range expansions were suggested as dispersal events. The split of clade III from clades IV–VI was estimated as vicariance (node 3). Several independent dispersal events were detected within clades IV and V, respectively.

## Discussion

### Infrageneric relationships within *Pyrrosia* s.l

Six main clades and eight subclades were recognized according to the cpDNA phylogenetic reconstructions of *Pyrrosia* s.l. in this study. We suggested 11 groups for the infrageneric delimitation of *Pyrrosia* s.l.

*P. schimperiana* was resolved as clade I which clustered with *Platycerium*. This results was similar to the studies of Testo and Sundue^47^ and Zhou *et al*.^35^, but different from the most studies that treat *P. schimperiana* as the basal group of *Pyrrosia* ^44,46^, Zhou *et al*.^35^ established a new genus *Hovenkampia*, and described its diagnostic characters different from *Pyrrosia* s.s. as: rhizomes completely parenchymatous; stomata polocytic; perispore thin, tightly adhering to the exospore surfaces. As Hovenkamp^4^, Hennipman^10^ and Uffelen and Hennipman^24^ pointed that the characters above are all not synapomorphy of P. africana-group. Rhizomes of the *P. rhodesiana* (P. porosa-group) are parenchymatous. Stomata of *P. mannii* and *P. penangiana* are polocytic. P. africana-group sheared the finely granulose epispore ornamentation with the species in clade VI of our study. Furthermore, scales of this species are pseudopeltate; the fronds are monomorphic, oblanceolate and estipitate; the indumenta are monomorphic and indumenta rays are narrow boat-shaped, these characters are all shared by more than one group of *Pyrrosia* s.s.

Clade II is monophyletic and as the basal group of *Pyrrosia* s.s. lineage. Species of this clade are all monomorphic fronds, short rhizome, basifixed scales. Smooth spores are the homogeneous characters of this clade. This clade is in the equivalent of P. costata-group of Hovenkamp^4^, Pyrrosia Clade of Zhou *et al*.^35^, and subg. *Pyrrosia* of Vasques *et al*.^46^.

Clade III contained only *P. niphoboloides*, which was assigned to the P. piloselloides*-*group by Hovenkamp^4^ (or members of previous segregated genus *Drymoglossum*) along with *P. piloselloides*. However, these species did not cluster together in the present cpDNA phylogenetic trees, with *P. piloselloides* clustered in clade V, which indicates that the P. piloselloides*-*group described by Hovenkamp is polyphyletic, although the members have similar morphological characters (*e.g*. dimorphic fronds, venation, and sori arrangement). While, *P. niphoboloides* was included in Galeoglossa subclade, which is in the equivalent of subclade B of clade IV of our study in Zhou *et al*.^35^. Vasques *et al*.^46^ treated this species in subg. *Solis*, which was the basal group of *Pyrrosia* except subg. *Lunae*.The sequences of *P. niphoboloides* we used were downloaded from GenBank. The phylogenetic position of *P. niphoboloides* still need more evidences.

Members of clade IV are characterized by long-creeping rhizomes, and peltate scales, but they have various frond shapes, indumenta and venation types, and spore ornamentation. This clade has four relatively independent subclades. Species of this clade are widely distributed in East and South Asia, and extend to the Indonesia Archipelago. The basal subclade A, which includes only *P. angustissima*, had been previously described as the segregated genus *Saxiglossum* ^26^. We recognized that *P. angustissima* belonged to the basal member of clade IV.*P. angustissima* has some autapomorphy characters, including linear-triangular rhizome scales, linear laminae with involute margins and a special type of drynarioid venation^26,31,61^. These features of *P. angustissima* clearly distinguish it from other members of clade IV, which makes it a separate subgroup. Subclade B contains three species, *P. nummulariifolia*, *P. kinabaluensis* and *P. rasamalae*, all of which belonged to the P. albicans-group of Hovenkamp. These species have succulent lamina with distinct water-tissues, dimorphic indumenta (acicular and woolly rays), and spores with longitudinal ridges and finely granulose, but they lack hydathodes. Subclade C contains *P. angustata* and *P. samarensis*, which belong to P. angustata-group of Hovenkamp. This subclade has similar morphology, including dimorphic fronds, entire margin scales, pseudo-drynarioid venation, and spores with longitudinal ridges (which occurs in only this subgroup). Subclade D, which was recognized as monophyletic, includes *P. laevis*, *P. ensata* and the P. lingua-group. The close relationship between the P. lingua-group and *P. laevis* and *P. ensata* revealed here agrees with the findings of Yang^25^, who assigned them to section *Pyrrosia*. Hovenkamp did not consider the P. lingua-group to be a well-established monophyletic group because the morphology of the spore was heterogeneous and there are no autapomorphies in this group. We found that species of subclade D all featured long-creeping rhizomes and fronds from monomorphic to moderately dimorphic with distinct stipitate and lamina ovate to lanceolate. In this subclade, persistent boat-shaped ray hairs exist in all species and coarsely sparse tubercules are the main spore ornamentation. The presence of common features in subclade D suggests that this group is monophyletic. Clade IV was mostly similar to subg. *Niphopsis* of Vasques *et al*.^46^ and Niphopsis clade of Zhou *et al*.^35^ with *P. niphoboloides* an exception. Nevertheless, the phylogenetic relationships among species, especially the species of subclade D were still uncertain.

Clade V includes subclades E and F. Subclade E contains only *P. piloselloides*, which is a member of the P. piloselloides-group. Subclade F contains species of the P. confluens-group, the P. lanceolata-group and an undecided species, *P. foveolata*. Both of the former two groups were considered as monophyletic by Hovenkamp^4^, on the basis of restricted hydathodes, monomorphic indumenta, large sori with short paraphyses (in the P. confluens-group), and sunken sori with centrally situated paraphyses (in the P. lanceolata-group). Nearly all species of these two groups and *P. foveolata* have common features, such as: long-creeping rhizomes, fronds that are dimorphic in various ways (with the exception of *P. longifolia*), lamina that are elliptic or elongated to strap-shaped with decurrent base and indistinct stipes, sori that are sunken with distinct stellate paraphyses, indumenta that are monomorphic and persistent, indumenta rays that are short boat-shaped, and perispore that are bilateral with tuberculate and irregularly verrucated protuberances. Evidence was sufficient to support the P. confluens-group, P. lanceolata-group and *P. foveolata* as monophyletic. Clade V of our study was equivalent to subg. *Niphobolus* of Vasques *et al*.^46^ and Niphobolus clade of Zhou *et al*.^35^. *P. rasamalae* was included in Niphobolus clade inexplicably in Zhou *et al*.^35^, while, this species was treated as a member of Clade IV in our study with highly support.

Clade VI occupies the most obvious common characters, including thick and short rhizomes, monomorphic fronds, and distinct hydathodes. Subclades G and H are resolved as separate monophylies with highly supported values. Subclade G contains *P. bonii*, *P. calvata*, *P. subtruncata*, *P. fengiana*, *P. sheareri*, *P. drakeana* and *P. subfurfuracea*. Three species of the P. sheareri-group together with *P. polydactyla*, *P. hastata*, and *P. flocculosa* are nested in subclade H, which contains most species of the P. porosa-group. Our results are quite different from Yang’s treatment, in which *P. subfurfuracea* and *P. calvata* were added to the P. costata-group based on similar thick and short rhizomes, un-peltate (basifixed and pseudopeltate) scales and other features^25^. Subclade G shares the following common features: pseudopeltate or basifixed scales, monomorphic fronds with a distinct stipe, densely granulated perispores, and dimorphic indumenta with acicular rays appressed to a layer with woolly rays (indumenta of *P. sheareri* are appressed and boat-shaped). Subclade H contains species of the P. porosa-group, three species belonging to the P. sheareri-group and three undecided species - *P. transmorrisonensis*, *P. mannii*, and *P. penangiana*. Two separate species, *P. mannii* and *P. penangiana*, were resolved as the basal lineages of subclade H. *P. polydactyla* and *P. hastata* formed the sister group of the lineages including the P. porosa-group and *P. flocculosa*. The frond shape of *P. hastata* and *P. polydactyla* is most unique in *Pyrrosia* s.l., and both are pedately divided to 4/5 depth of the frond into 3–5 to 6–8 divisions with distinct stipes. The indumenta are boat-shaped and persistent. The other species of subclade H share many common features, for example, linear to narrow lanceolate and oblanceolate fronds with gradually narrowed and decurrent fronds base, and fronds estipitate; the indumenta are persistent and dense with acicular rays, and in most species, acicular rays are appressed to a layer with mainly woolly rays (woolly ray stellate hairs are not obvious in *P. gralla*, *P. assimilis*, and *P. penangiana*). Although *P. polydactyla* and *P. hastata* shared some morphological characters with subclade G, they were polyphyly in our study, and paraphyly in Vasques *et al*.^46^ and Zhou *et al*.^35^. Zhou *et al*. combined them as a *P. sheareri* group^35^.

### Incongruence between the cpDNA and *LEAFY* phylogenetic trees and potential reticulate evolution

The chloroplast genome is maternally inherited in ferns, and the nuclear genome exhibits amphilepsis. Consequently, comparative studies of these two genomes might uncover potential reticulate evolution, including introgression and/or hybrid speciation^62^. *LEAFY* is a well-studied single-copy gene in ferns, and it has been successfully used to resolve reticulate evolution at low taxonomic levels^63,64^. Considering the high proportion of hybridization in ferns and the advantages of *LEAFY*, both cpDNA and *LEAFY* were included in the phylogenetic analyses.

Each of four clades I, II, V and VI was resolved as monophyletic with high support values, both in the cpDNA and *LEAFY* gene trees. CpDNA and *LEAFY* gene trees showed incongruent phylogenetic positions for clade I and clade V. Both of clade I and V nested in clade IV in the nuclear tree. Hybridization and incomplete lineage sorting (ILS) are two important factors that lead to phylogenetic incongruence^65–67^. According to the result of ancestral area reconstruction, the potential ancestral area of clade I was area E, ancestral areas of clade V are area B or E, and the ancestral area of clades IV and VI was B. Dispersal events have been inferred between area B and E. By contrast, ILS is resulted from the ancestral alleles being sorted into some lineages randomly. In our study, both alleles of clade I and V nested in clade IV, thus, hypothesis of ILS seems less plausible although it is difficult to distinguish from ancient hybridization, especially without genomic data.

We suggested that clade V might be an ancient hybrid origin and reached the current distribution areas during species dispersal in history. Ancestral species of clade IV might be the male parent of clade V and ancestral species of clade VI might be the female parent of clade V. We suggested three potential scenarios of the origin of clade I: (1) the new established genus *Hovenkampia* was an ancient hybrid origin, ancestral species of *Platycerium* were the female parent and *Pyrrosia* s.s. were the male parent; (2) *P. schimperiana* is hybrid species with other species in *Hovenkampia* and species in clade IV as its parents; (3) three individuals of *P. schimperiana* we used in this study are hybrid individuals.

In subclade H, *P. porosa* had two divergent homoeologous copies of *LEAFY*, one copy of which clustered with *P. nudicaulis*, while another copy clustered with *P. assimilis* and then nested with *P. tonkinensis*. In the cpDNA tree, individuals of *P. porosa*, *P. nudicaulis*, and *P. assimilis* were clustered together and were distinctly separated from *P. tonkinensis*. In addition, the basal chromosome number within *Pyrrosia* s.l. was 36 or 37; most of the reported data of *P. porosa* are tetraploid and hexaploid^4^, only one diploids of *P. porosa* have been reported in India^68^. It revealed that some plants of the *P. porosa* might be allopolyploid. Furthermore, one individual of *P. gralla* clustered with *P. davidii*, and the copies of another individual nested in *P. tonkinensis* in the phylogenetic tree of *LEAFY*, while it was distinctly separated from *P. tonkinensis* in the cpDNA tree. The short branches of cpDNA trees (Fig. 1) and molecule dating (Fig. 3) showed that *P. porosa*, *P. nudicaulis*, *P. assimilis*, *P. tonkinensis*, and *P. gralla* were differentiated recently. Based on the results of cpDNA and nrDNA, a recent hybrid or reticulate evolution were revealed in P. porosa-group. In subclade D, *P. lingua* also had two divergent homoeologous copies of *LEAFY*, a copy of which clustered with *P. martini*, while another one clustered with *P. petiolosa* in the phylogenetic tree of *LEAFY*. However, individuals of *P. lingua* and *P. petiolosa* were distinctly separated in the cpDNA tree. Only diploids were reported in *P. lingua* and *P. petiolosa*. Incongruence between the cpDNA and *LEAFY* trees suggested a recent hybrid might exist in P. lingua-group. Nevertheless, owing to the limited information on chromosome numbers of *Pyrrosia* s.l., the acquisition of more exact results will require more samples and further comprehensive cytological studies.

### Morphological characters assessment and evolution

**Figure 4:**
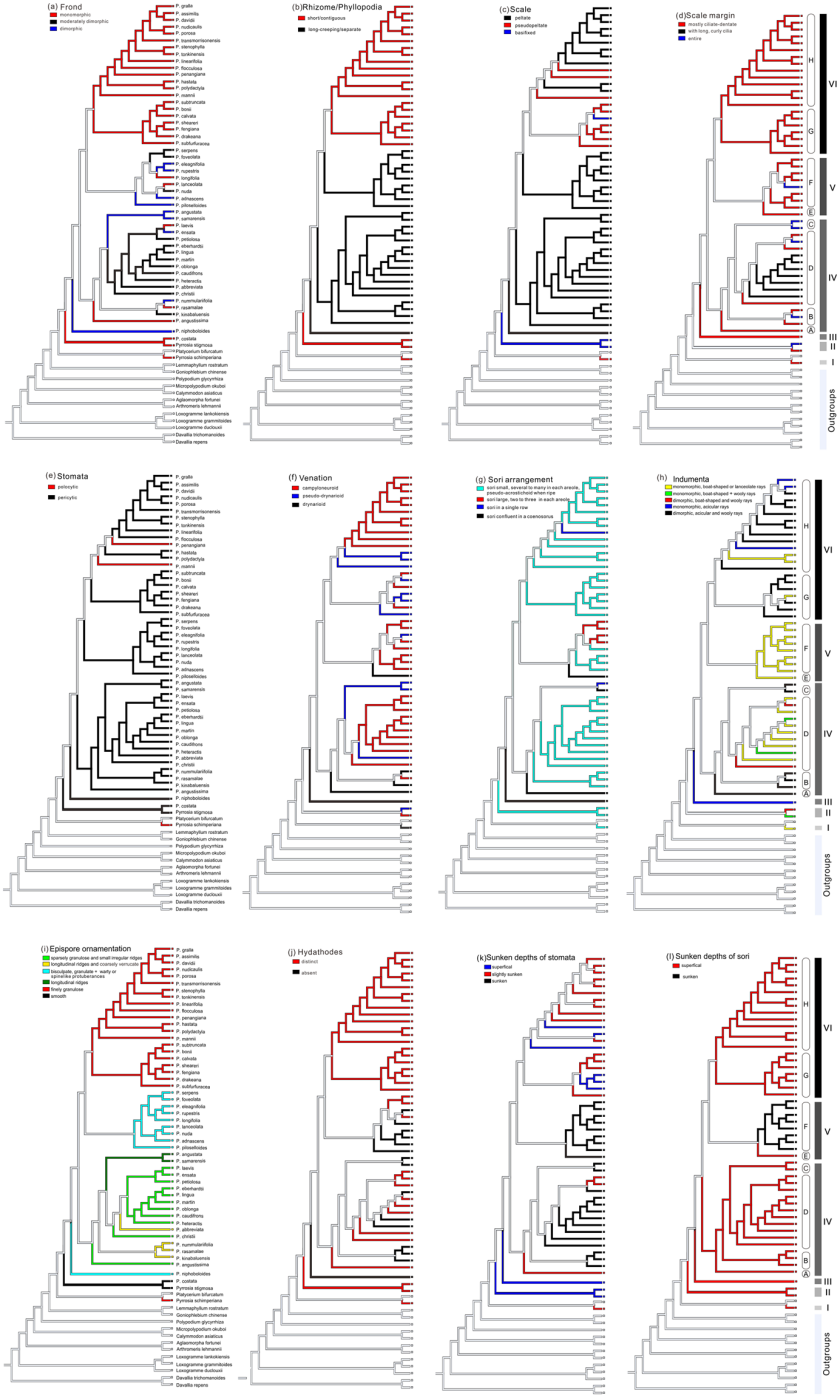
Selected characters evolution optimized onto the tree set obtained from the maximum likelihood (ML) analysis of cpDNA data set includes one individual for each species based on likelihood method in Mesquite.

Several features, including the presence of specialized sterile fronds, rhizome growth-form, the distance between adjacent phyllopodia, the insertion type of rhizome scales, scale margin morphology, stomata type, venation type, sori arrangement, indumenta shape and epispore ornamentation, were all treated as diagnostic characters for infrageneric classification and species delimitation of *Pyrrosia* s.l. in previous studies^2–4,6,8,20,22–24,31,61,69^. If we only use morphologic characters to evaluate the infrageneric classification in *Pyrrosia* s.l., most groups or sections are not monophyletic^4,25^. Ancestral character state optimization based on a stable molecular phylogenetic tree in this study enabled a synthetic evaluation of all diagnostic characters (Fig. 4).

(1) The presence of specialized fertile fronds is essentially different from monomorphic and moderately dimorphic fronds, in which fertile fronds are the same or longer and narrower than sterile fronds. Dimorphic fronds appeared in clade III and scattered in clades IV and V. Hovenkamp^4^ considered that monomorphic fronds transformed in the presence of specialized sterile fronds; thus, it can be observed that dimorphic fronds have evolved multiple, independent times in *Pyrrosia* s.l. (Fig. 4a). (2) Rhizome growth-forms and the distance between adjacent phyllopodia are relevant characters. Short and thick rhizomes correspond with contiguous phyllopodia and long-creeping rhizomes correspond with separate phyllopodia. Long-creeping rhizomes are homoplastic characters, occurring in clades III, IV and V (Fig. 4b). (3) Regarding the insertion type of rhizome scales, basifixed scales are considered ancestral, and they occur in clades II and VI. Peltate scales are the predominant type in *Pyrrosia* s.l., and both basifixed scales and pseudopeltate scales appear to have evolved at least five times (Fig. 4c). (4) Scales with ciliate-dentate margins are the mostly ancestral types, and scales with long and curly cilia margins are apomorphic within clade VI, while scales with entire margins independently evolving at least five times (Fig. 4d). (5) The dominant pericytic stomata type of *Pyrrosia* s.l. is a deuterogenic feature in Polypodiaceae. Three species of *Pyrrosia* s.l. (*P. schimperiana*, *P. mannii* and *P. penangiana*) still have polocytic stomata, which are common in other genera of Polypodiaceae. Polocytic stomata in *Pyrrosia* might be the result of reversion or secondary development (Fig. 4e). (6) Pseudo-drynarioid and campyloneuroid venation types of *Pyrrosia* s.l. are deuterogenic features in Polypodiaceae, and drynarioid venation is the ancestral feature. Species occupying pseudo-drynarioid and campyloneuroid venation were both polyphyletic, and they interspersed in clade II, IV, V and VI. A high frequency of homoplasy of venation types appears within *Pyrrosia* s.l. (Fig. 4f). (7) Sori arrangement is an important diagnostic character in *Pyrrosia* s.l. and the other ferns. Confluent sori and orbicular and discrete sori have been treated as the diagnostic character even at the species and genus level in Polypodiaceae^69–72^. Coenosori was be considered as one of the main characters to separate the previous genus *Drymoglossum* from *Pyrrosia* s.l., although some authors considered this classification “purely artificial”^69^. The homoplasy of confluent sori in *Pyrrosia* s.l. was confirmed in this study. Meanwhile, the other three types of sori arrangement were also homoplasy (Fig. 4g). (8) There are four main shapes of stellate hairs in *Pyrrosia* s.l.: hairs with straight acicular rays, those with straight boat-shaped rays, those with curly wooly rays, and those with straight boat-shaped and curly wooly rays at one axis. The indumenta of each species may be monomorphic or dimorphic. Monomorphic indumenta contain only one type of stellate hair above, while dimorphic indumenta contain two types of stellate hairs. We recognized five types of indumenta in *Pyrrosia* s.l. (Fig. 4h) and found that both monomorphic and dimorphic indumenta are homoplastic. Dimorphic indumenta occurred in clades II, IV and VI, while monomorphic indumenta occurred in all clades. In addition, clades I, II and V, and subclades D all have boat-shape or lanceolate hairs, and the other clades (subclades) except *P. hastata*, *P. polydactyla*, and *P. sheareri* have acicular hairs. Overall, indumenta of *Pyrrosia* s.l. are homoplasy (Fig. 4h). (9) Epispore ornamentation appears relatively unique for each clade or subclade. Clades III and V share the obviously bisculpate spores with dense, small granulate and warty or spike protuberances, and clades I and VI have spores with more or less dense finely granulose protuberances. Smooth spores appear only in clade II, and spores with longitudinal ridges appear only in subclade C. These two types of epispore ornamentations can be treated as synapomorphies of clade II and subclade C, respectively. Epispore ornamentation of subclades A and D appears sparsely granulose and irregularly ridged (with the exception of *P. abbreviata*), and those of subclade B and *P. abbreviata* of subclade D are longitudinally ridged with finely granulose protuberances (Fig. 4i).

Ancestral character state optimization revealed high levels of homoplastic evolution in *Pyrrosia* s.l. Only the epispore ornamentation is synapomorphic in clade II and subclade C, respectively. There is no single character that can be used as an apomorphy to distinguish groups (clades and subclades) in *Pyrrosia* s.l. from each other; thus, the combination of two or more characters are necessary to identify all groups. For instance, given that rhizome growth-forms are similar between subclades G and H and clade II, combining this character with the indumenta morphology and the fronds shape can identify clade II, while combining the frond shape and insertion type of rhizome scales can identify subclades G and H. Furthermore, some of the anatomical characters, such as the distribution pattern of collenchyma, sclerenchyma and parenchymatous cells in rhizomes, might be helpful in defining traits for monophyletic group recognition and species identification in *Pyrrosia* s.l.^6,22^.

### Ecological adaptations

Most species of *Pyrrosia* s.l. are extremely drought tolerant. The morphological characters such as the texture of the lamina that is coriaceous, thick-leathery, succulent, or peronate; the presence of a thick cuticle on the epidermis; sunken stomata; sunken sori; or distinct hydathodes are all xerophytic adaptations. Regarding drought resistance, poikilohydrous and succulent forms are considered two different growth-forms^4^. Poikilohydrous plants of *Pyrrosia* s.l. can roll and stretch their fronds in response to drought resistance, which are similar to resurrection plants. Most of these species, such as *P. porosa*, *P. schimperiana*, are short rhizomes, the anticlinal walls of the adaxial epidermis appear thin or slightly thickened, hypodermis and water tissue are absent or indistinct, and the indumenta form dense mat. Poikilohydrous plants are adaptable to seasonal climates with long dry periods. By contrast, succulent plants of *Pyrrosia* s.l. species possessing fronds with thick water tissues, such as *P. abbreviata*, *P. angustissima*, *P. longifolia* and *P. nummulariifolia*, can survive by storing water during short periods of drought. These species are long-creeping rhizome, the adaxial epidermis are strongly or moderately thick, and stomata are sunken or slightly sunken. In the phylogenetic tress, poikilohydrous plants of *Pyrrosia* s.l. nested in clades I, II and VI, while, succulent form species nested in clades III, IV and V. These two growth-forms adapting to drought resistance are all independently polyphyletic in *Pyrrosia* s.l.

In addition to morphological specializations, another key innovation associated with the success of *Pyrrosia* s.l. in more arid habitats is the form of photosynthesis known as crassulacean acid metabolism (CAM). The CAM cycle has been reported in five species of *Pyrrosia* s.l., namely, *P. confluens*, *P. dielsii*, *P. lanceolata*, *P. longifolia*, and *P. piloselloides* ^11,12,15,18^, while the C3 pathway is employed in *P. rupestris*, *P. costata*, *P. flocculosa*, *P. porosa*, *P. schimperiana* and *P. sheareri*. CAM is an important adaptation of photosynthetic carbon fixation, and CAM plants restrict gas exchange with the atmosphere during the daytime. The water use efficiency of CAM plants is better than that of C3 plants, and consequently, CAM plants can adapt to many tropical and subtropical environments with intermittent or strong seasonal water supply^73^.

Chloroplast-containing cells of CAM plants fix CO_2_ initially at night, and during the daytime, organic acids behind closed stomata create an internal CO_2_ source that is re-assimilated by rubisco in the chloroplast^74^. Stomatal behaviour is relate to water pressure and CO_2_ concentration^75^. CAM is correlated with various anatomical and morphological features responding to water pressure. We found that stomata of species that utilize the CAM pathway are deep sunken, stoma grooves are strongly contracted above the stomata, and the number of epidermal cells adjoining the stoma groove is 6–10 (12)^27^ (Supplementary Fig. S2). Furthermore, these species live in exposed trunks or rocks and occupy more exposed microhabitats, even living together with C3 epiphytes^15^. Based on the available ecological information and the characteristics of stomata and the epidermal cells^16,74^, we suggested that species utilizing CAM pathway including the five has been reported clustered in clade V. CAM pathway might have a single origin in *Pyrrosia* s.l. The results of our molecular dating indicate that the divergence time of clade V was dated to ca. 17.98 Ma (node 11), which revealed an early Miocene origin of the CAM pathway in *Pyrrosia* s.l.

The emergence of CAM photosynthesis in different taxa may be driven by the same external driving forces. Just as the occurrence of CAM pathways in Bromeliaceae was driven^73^, progressive aridification and declining CO_2_ concentrations during the Oligocene and Miocene may have gradually favored for the emergence of CAM photosynthesis in *Pyrrosia* s.l.

### Diversification of *Pyrrosia* s.l. 

The poor fossil record of polygrammoid ferns discourages estimations of exact differentiation times and recognition the distribution area of ancestors. Molecular dating by Schuettpelz and Pryer^60^ showed that *Pyrrosia* s.l. originated in Oligocene (ca. 35.1 Ma). Testo and Sundue^47^ estimated the origination time between *Pyrrosia* s.l. and *Platycerium* to be 63.41 Ma. The diversification of most of the main clades of *Pyrrosia* s.s. in Testo and Sundue’s study were also dated back to the Oligocene, with a steady period of ca. 20 My from the early Eocene to the early Oligocene^76^. Therefore, we believe *Pyrrosia* s.l. originated no later than Oligocene and underwent diversification during the Oligocene and Miocene.

Ancestral area reconstruction based on the DEC model in RASP 3.2 revealed that Southern Asia, the Indo-China Peninsula and southwestern and southern China (area B); and Africa (area E) are the probable ancestral areas of *Pyrrosia* s.l. Janssen *et al*.^44^ suggested that *Pyrrosia* s.l. might be of African origin because the African species *P. liebuschii* was the basal clade of *Pyrrosia* s.l. Holtum^5^ suggested that the African species may have dispersed from Asia because *P. sheareri* is closer to the primitive conditions, and this species predominantly lives in China with a centre of distribution located in Southeast Asia. Although *P. sheareri* is neither the ancestor nor the basal species of *Pyrrosia* s.l. based on our phylogenetic analyses, area B, particularly the Himalayan region, has a considerably higher species diversity. Furthermore, the phylogenetic analyses based on *LEAFY* determined that *P. costata* (representing clade II), was the basal clade, and the Africa species *P. schimperiana* (clade I) was nested into clade IV. The ancestral area of clade II was assumed to be area B or areas B and C, which further demonstrated that area B is most likely the original area of *Pyrrosia* s.l. Moreover, this area also displays high species diversity in nearly all subfamilies in Polypodiaceae, and it is another diversity center outside of the tropical Americas^77^. Southeast Asia is the origin area of *Thylacopteris* ^40^, *Microsorum* ^78^, *Lepisorus* ^79^ and drynarioid ferns^45^, as well as some taxa in Eupolypods II *Deparia* ^53^ and some angiosperms^49,55^. Similar patterns were also found in the closely related microsoroids, which have a diversity centre in southeastern Asia and colonized to African regions several times^44^.

The distribution of *Pyrrosia* s.l. presents an Africa-Asia disjunction, which is common in plants and has recently attracted much attention^42,44,49,54,55,57,80–83^. Four main driving mechanisms for the disjunctive distribution have been summarized: (1) transoceanic long-distance dispersal, (2) overland migration via land bridges, (3) boreotropical dispersal via Eocene forests, and (4) rafting of India^49,54,82,83^. Hovenkamp^4^ proposed that the disjunctive distribution pattern of *Pyrrosia* s.l. resulted from vicariance, such as the breakup of Africa from Gondwana (120–140 Ma) and the separation of Australia from India (125 Ma), rather than dispersal. The Indian plate became progressively more isolated from eastern Gondwana in the Cretaceous and Paleocene, and it moved northward towards Asia with glancing contact to Asia at ca. 57 Ma^84^. In our analyses, the divergence time of Africa lineages and Asia-Australasian lineages was estimated to be much younger (Fig. 3b); thus, the split could not be vicariance resulting from the breakup of Gondwana and Laurasia, and the Indian plate as either a raft or stepping stone is therefore too old for the origin of *Pyrrosia* s.l. In addition, the “boreotropical” floristic connection hypothesis, in which the plants moved across the North Atlantic during late Paleocene to the middle Eocene, presents a time frame that is too early to explain the Africa-Asia disjunction of *Pyrrosia* s.l.^85,86^. The closure of the Tethys Sea led to the direct connection between mainland Africa and western Asia during the early Miocene^87,88^, and the split time of the African lineages and Asia-Australia lineages seems to fit the time frame of the overland migration hypothesis (Fig. 3b). However, no species or fossil records of *Pyrrosia* s.l. have been reported in northern Africa or the adjacent Asian area, which weaken the support for this hypothesis. Overland migration is therefore an alternative dispersal scenario. Transoceanic long-distance dispersal has been used extensively to explain the intercontinental disjunction of plants^49,52–54,57,82,83,89,90^. Fern spores are minute, and like wind-dispersed seeds and pollens, they may have been transported transoceanic by prevailing monsoon wind systems or ocean currents between Africa and northern India^44,53,57,91^. Transoceanic long-distance dispersal might be the most plausible hypothesis to clarify the Africa-Asia disjunction of *Pyrrosia* s.l.

Within *Pyrrosia* s.s., multiple dispersal events from area B to C and sequentially to area D, and from area B to A can be inferred. Southwest Asia suffered frequent orogenesis, particularly the Himalayan regions, after experiencing many rapid uplifts and unroofing^92^. Meanwhile, Southeast Asia also suffered a complex interplay of plate movements and grew incrementally by the addition of continental fragments. Australia collided with Southeast Asia ca. 25 Ma years ago^93^. Frequent and severe geological movements during the Miocene provided the possibility for the dispersal between areas. The global climate fluctuated dramatically from the late Oligocene, as the monsoon system was established and subsequently strengthened by the late Oligocene warming, mid-Miocene climatic optimum and persistent Miocene cooling^86,94,95^. Dramatic climate fluctuation may have triggered the speciation and diversification of *Pyrrosia* s.l., and frequent habitat fragmentations and range transition may have led to the accumulation of species diversity.

We infer the following diversification scenario for *Pyrrosia* s.l.: *Pyrrosia* s.l. originated from Southern Asia, the Indo-China Peninsula and southwestern and southern China (area B) before the Oligocene, and the global climate subsequently underwent a rapid cooling at the late Eocene and the early Oligocene with the temperature declining ca. 5 °C^86^. Contemporaneously, Southeast Asia, particularly the Himalaya regions, experienced aridification. The sudden cooling and aridification may have been a devastating blow for *Pyrrosia* s.l. Some of the ancestral species may have migrated or dispersed to the much warmer southern areas and finally reached Africa. It is undisputed that climate cataclysm and long-distance dispersal can cause species extinction. The species of *Pyrrosia* s.l. that reached Africa were random, and thus, the African species are polyphyletic rather than monophyletic. A steady time interval of nearly 20 My before 34 Ma in Testo and Sundue’s^47^ study may also indicate lower speciation rates or/and increased extinction rates of *Pyrrosia* s.l. in that time. Fossil pollen data indicated that Oligocene climate and vegetation can be characterized as “tropical seasonal” but under fairly humid conditions in East Africa, and rain forests experienced strong successive retreats in the subsequent time period^96^. Ecosystems began to transform into much drier grassland, and many ecosystems, including widespread grasslands/savannah with many new C4 plants, were established across eastern Africa in the mid-Miocene based on grass macrofossils and pollen data. After 17 My of cooler conditions, the global climate reached a warm phase peak during the late middle Miocene (15–17 Ma), known as the mid-Miocene Climatic Optimum. During the Miocene, speciation of Asia-Australia groups was flourishing, which may have been triggered by frequent geological movements and dramatic climatic fluctuations during that time. In contrast, lower average speciation rates or/and increased extinction rates resulted in less diversity in ferns in Africa^97^, which further resulted in a disproportional species number between Africa and Asia-Australia.

## Materials and Methods

### Sample collection

Our taxon sampling strategy was designed to include all six subfamilies of Polypodiaceae: Platycerioideae, Loxogrammoideae, Drynarioideae, Microsoroideae, Polypodioideae and Grammitidoideae and its sister group Davalliaceae based on the latest classification for extant lycophytes and ferns, PPGI^1^. In Platycerioideae, all groups of *Pyrrosia* s.l. proposed by Hovenkamp^4^ as well as the two previously segregated genera *Drymoglossum* and *Saxiglossum* were included. In total, 112 individuals representing 51 species of *Pyrrosia* s.l. were sampled (Supplementary Table S2). One to five individuals from the different regions were collected for each species. 19 individuals were used besides *Pyrrosia* s.l.: *Platycerium bifurcatum* (Cav.) C. Chr., *Pl. coronarium* (O.F. Müll.) Desv. and *Pl. wallichii* Hook were sampled in *Platycerium*. Samples of Loxogrammoideae, Drynarioideae, Microsoroideae, Polypodioideae and Grammitidoideae and its sister group Davalliaceae were chosen as outgroups for the phylogenetic analyses.

Fresh healthy leaves were dried immediately in silica gel, and kept dry until DNA extraction. The voucher specimens were deposited at the Herbarium of Institute of Botany, the Chinese Academy of Sciences (PE) and the Herbarium of Institute of Medicinal Plant Development, Chinese Academy of Medical Sciences (IMD). Voucher information is listed in Supplementary Table S2.

### DNA extraction, PCR amplification, cloning, and sequencing

Total genomic DNA was extracted from silica gel-dried leaves or herbarium material using the Plant Genomic DNA Kit (Tiangen Biotech, Beijing, China) following the manufacturer’s instructions.

For each individual, five cpDNA regions (*matK*, *rbc*L, *psb*A–*trn*H and *rps*4 + *rps*4–*trn*S) and the nuclear gene region (*LEAFY*) were separately amplified with standard PCR. The *mat*K region was amplified using primers and PCR protocols introduced by the CBoL Plant Barcoding Working Group (http://www.barcodinglife.org/index.php/Public_Primer_PrimerSearch). The *rbc*L region was amplified using primers 1F^98^ and 1351R^99^, following the PCR protocol described by Hasebe *et al*.^100^. The *psb*A–*trn*H region was amplified using primers *psb*AF and *trn*HR according to the protocol outlined by Chen *et al*.^101^. Amplification primers and protocols to amplify *rps*4 + *rps*4–*trn*S were those described by Nadot *et al*. Smith and Cranfill^102,103^. *LEAFY* was amplified using primers 1dF and 3dR, which were designed by Zhao^48^.

The PCR products were purified using PEG 8000 or the TIANgel Midi Purification Kit (Tiangen Biotech, Beijing, China) following the manufacturer’s protocol. Then they were directly sequenced in both directions using the amplification primers with an ABI Prism^TM^ BigDye Terminator Cycle Sequencing Ready Reaction kit (Perkin Elmer, Norwalk, CT, USA). Sequences were analysed using at the ABI 3730XL automated sequencer (Applied Biosystems, Foster City, CA, USA). Cloning of samples with allelic variation in *LEAFY* was conducted with a pEASY-T3 Cloning Kit according to the manufactures’ protocols (Transgen Biotech), and 6 to 12 clones were sequenced for each sample. GenBank accession numbers are listed in Supplementary Table S2.

### Phylogenetic analysis

Sequences were assembled in the ContigExpress program of the Vector NTI Suite v.6.0 (Informax, North Bethesda, Maryland, U.S.A.). New combined sequences were assembled in single-region datasets that were aligned using CLUSTAL X v1.83^104^ and then manually adjusted in BioEdit v.7.1.11^105^. Phylogenetic analyses of the combinded cpDNA data set and *LEAFY* were performed using MP, ML and BI in PAUP * 4.0b10^106^, RAxMLv7.0.4^107^, and MrBayes v3.2.5^108^, respectively.

In MP analyses, all characters were equally weighted and gaps were treated as missing data. A heuristic search was performed with 1000 random addition replicates, tree bisection-reconnection (TBR) swapping and the MulTrees option in the analysis program. Bootstrap support values (BS_MP_), based on 1000 replicates with 10 random additions per replicate, and were used to estimate the confidence of the clades. We employed jModelTest v2.1.7^109^ to identify the best fitting models for ML and BI analyses. In the ML analyses, GTR + G was determined to be the best-fit model according to the Akaike information criterion (AIC) implemented in jModelTest and the BI analyses used the TIM1 + G model determined by the Bayesian information criterion (BIC) for cpDNA datasets. TrN + I + G was determined to be the best model by AIC and BIC for *LEAFY*. RAxML was conducted with the fast bootstrap option, using 1000 replicates. For BI analyses, four Markov chain Monte Carlo (MCMC) chains were run for 1,000,000 generations each, and were sampled every 1000 generations, starting with a random tree. The convergence of runs and estimation of burn-in were assessed using Tracer v.1.6^110^ and Bayesian posterior probabilities (PP_BI_) were calculated for the majority consensus tree of all sampled trees after discarding those sampled within the burn-in phase in MrBayes.

### Divergence time estimation

Bayesian approaches were employed of cpDNA data set to estimate the divergence times of *Pyrrosia* s.l. in BEAST v 1.8.0^111^ with an uncorrelated lognormal distributed (UCLD) relaxed clock model, the GTR + G substitution model and a Yule process tree prior. The MCMC chains were run for 100,000,000 generations with sampling every 1000 generations and at least 10% burn-in phase. The tree was calibrated at the most basal node (Polypodiaceae and Davalliaceae, 60.4 Ma) obtained from divergence time estimate carried out with leptosporangiate ferns^60^. The effective sample size (ESS) was estimated in Tracer v.1.6 to be >200 for each parameter. The maximum clade credibility tree with median branch lengths and a 95% highest HPD interval on nodes was compiled using TreeAnnotator v.1.8.0.

### Ancestral area reconstruction

Five biogeographical regions were defined: (A) eastern Asia (including central, eastern and northeastern China, Korea Peninsula, southern Japan and Far East); (B) southern Asia, the Indo-China Peninsula and southwestern and southern China; (C) Malesia (including Malaysia, Indonesia, and the Philippines); (D) Australasia (including Australia, New Guinea, New Zealand, and the South Pacific islands); and (E) Africa (including Madagascar) (Fig. 3a). The geographical distribution of *Pyrrosia* s.l. mostly followed Hovenkamp^4^ and referred to other literatures^3,8,32^. We carried out biogeography analyses using the DEC model^112,113^ implemented in RASP 3.2^114^. We inferred possible biogeographical scenarios across 1000 trees obtained from the BEAST analysis in the DEC analysis. A composite Akaike weight was used to summarize biogeographic reconstructions across trees^115^. The maximum area number was set to four because only *P. lanceolata* occupied four biogeographical regions.

### Morphological character evolution

The data employed for the reconstruction of the evolution of morphological characters were obtained and our own observations of morphological character variation using herbarium specimens (Supplementary Table S3) and referred to the previous publications^3–8,19–26^. In addition, we considered observations made during fieldwork.

The evolution of morphological characters was reconstructed with likelihood using Mesquite v3.04^116^. We input the tree set obtained from the ML analysis based on a simple cpDNA data set (supplementary Fig. S1). In order to exhibit the morphological characters clearly in the phylogenetic tree at species level, this data set includes one individual for each species. Due to the intraspecific variation of the sequences are small, we chose one individual randomly for each species. All characters were scored as discrete binary or multistate characters and treated as unordered and equally weighted (Supplementary Table S4).

## Electronic supplementary material

Supplementary information
