# Supplementary material for: Phylogeny, historical biogeography and characters evolution of the drought resistant fern Pyrrosia Mirbel (Polypodiaceae) inferred from plastid and nuclear markers
Source: Sci Rep. 2017 Oct 6;7:12757. doi: 10.1038/s41598-017-12839-w (PMC5630607; doi:10.1038/s41598-017-12839-w)
Supplement: Supplementary file 1 — Supplementary information [file 41598_2017_12839_MOESM1_ESM.pdf]

Supplementary information

Phylogeny, historical biogeography and characters evolution of the drought resistant fern *Pyrrosia* Mirbel (Polypodiaceae) inferred from plastid and nuclear markers

Xueping Wei[1], Yaodong Qi[1], Xianchun Zhang[2], Li, Luo[1], Hui Shang[3], Ran Wei[2], Haitao Liu[1] & Bengang Zhang[1,*]

[1]Key Laboratory of Bioactive Substances and Resources Utilization of Chinese Herbal Medicine, Ministry of Education, Institute of Medicinal Plant Development, Chinese Academy of Medical Sciences, Peking Union Medical College, Beijing, China.

[2]State Key Laboratory of Systematic and Evolutionary Botany, Institute of Botany, Chinese Academy of Sciences, Beijing 100093, China.

3. Shanghai Chenshan Plant Science Research Center, Chinese Academy of Sciences; Shanghai Chenshan Botanical Garden, Shanghai 201602, China.

*Correspondence and requests for materials should be addressed to B.Z. (email: bgzhang@implad.ac.cn)

Supplementary Figure S1. Phylogram of *Pyrrosia* s.l. obtained from the maximum likelihood (ML) analysis of the combined cpDNA data set which contains one sample for each species

Supplementary Figure S2. Photosynthesis form evolution and stomata of *Pyrrosia* s.l..

Supplementary Table S1. Different taxonomic treatments of *Pyrrosia* s.l..

Supplementary Table S2. Taxa, voucher and GenBank accession numbers for the sequences used in this study.

Supplementary Table S3. Specimens used for morphological characters observation.

Supplementary Table S4. Morphological character matrix.

Supplementary References

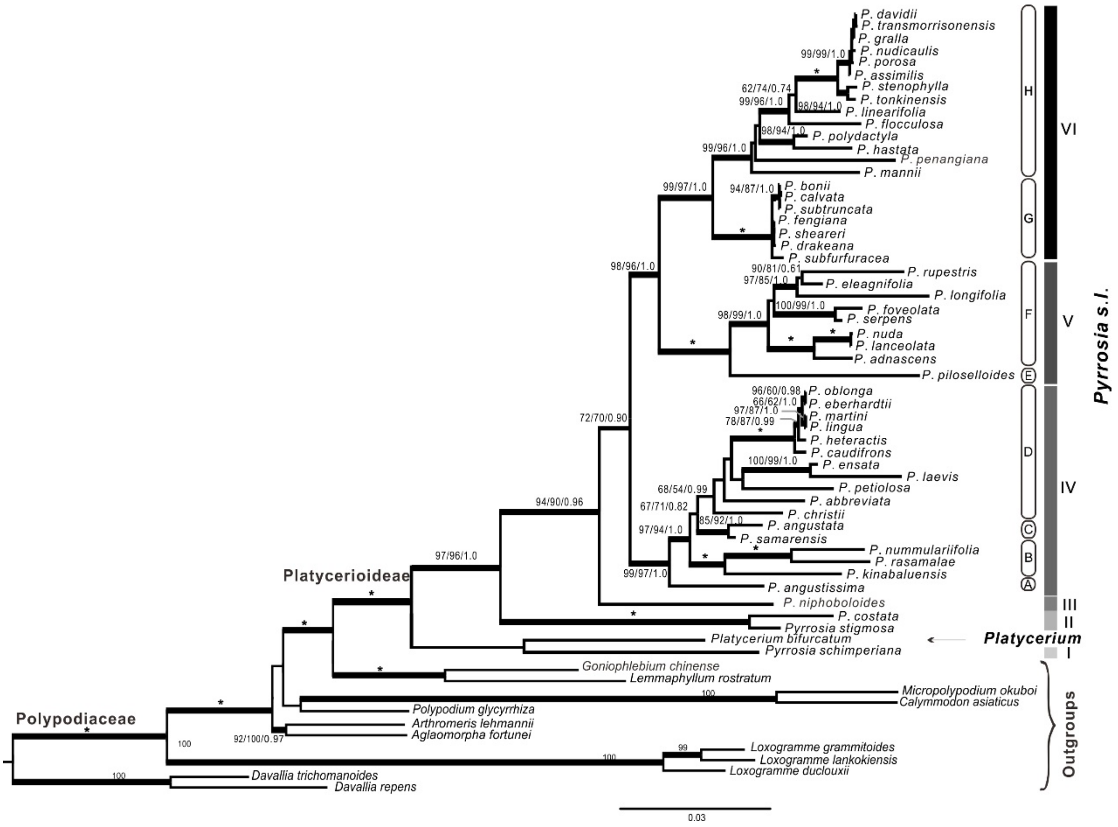

Supplementary Figure S1. Phylogram of *Pyrrosia* s.l. obtained from the maximum likelihood (ML) analysis of the combined cpDNA data set which contains one sample for each species, including sequences of *rbc*L, *mat*K, *psb*A-*trn*H, and *rps*4+*rps*4-*trn*S. Numbers on branches are support values (BS_{ML}/BS_{MP}/PP_{BI}). Bold branches indicate BS_{MP}, $BS_{ML} \geq 70\%$ and $PP_{BI} \geq 0.95$. Stars indicate BS_{MP}, BSML = 100% and PP_{BI}=1.0. Dash (-) indicates nodes with BS_{MP} or $BS_{ML} < 50\%$.

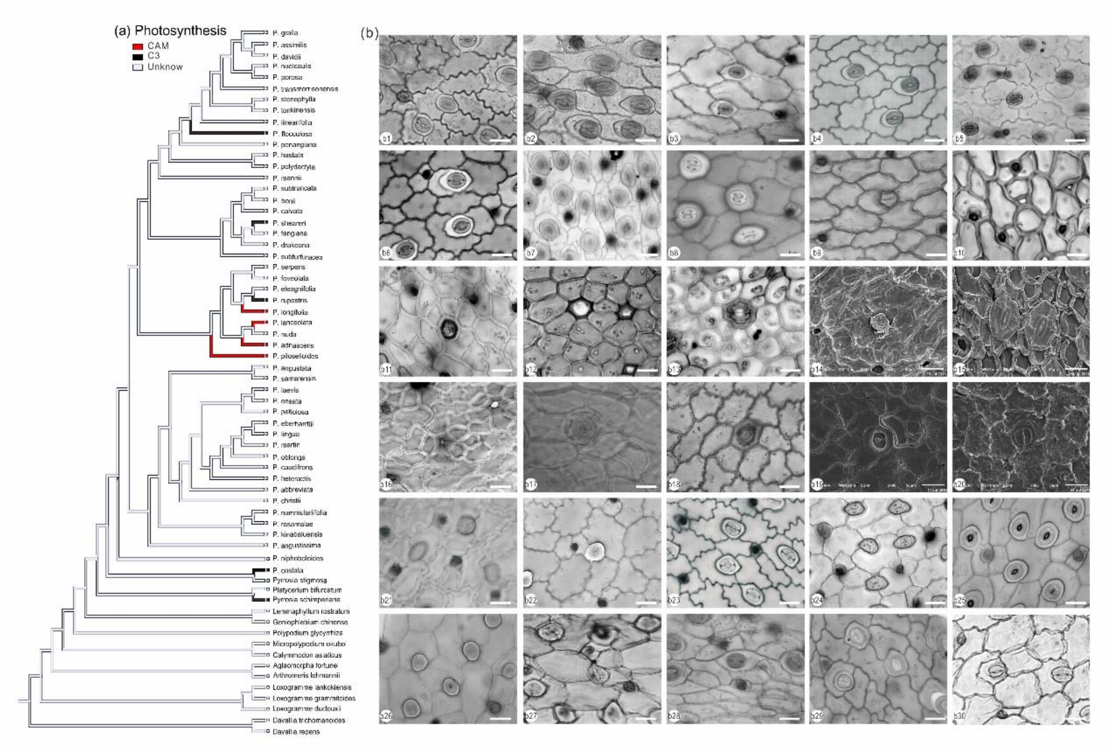

Supplementary Figure S2. Photosynthesis form evolution and stomata of *Pyrrosia* s.l..

(a) Photosynthesis form optimized onto the tree set obtained from the maximum likelihood (ML) analysis of cpDNA data set which contains only one sample for each species based on likelihood method in Mesquite. (b) Fronds abaxial epidermis showing stomata: b1. *Pyrrosia assimilis* (outside view under light microscope (LM), the same below without notes), b2. *P. porosa*, b3. *P. flocculosa*, b4. *P. penangiana*, b5. *P. mannii*, b6. *P. subtruncata*, b7. *P. sheareri*, b8. *P. serpens*, b9. *P. rupestris*, b10. *P. longifolia*, b11. *P. longifolia* (inside view under LM), b12. *P. lanceolata,* b13. *P. lanceolata* (inside view under LM), b14. *P. lanceolata* (outside view under scanning electron microscope (SEM)), b15. *P. lanceolata* (inside view under SEM), b16. *P. confluens*, b17. *P. confluens* (inside view under LM), b18. *P. piloselloides*, b19. *P. piloselloides* (outside view under SEM), b20. *P. piloselloides* (inside view under SEM), b21. *P. angustata*, b22. *P. laevis*, b23. *P. lingua*, b24. *P. heteractis*, b25. *P. abbreviate*, b26. *P. nummulariifolia*, b27. *P. rasamalae*, b28. *P. angustissima*, b29. *P. costata*, b30. *P. schimperiana*; scale bar=50μm.

Supplementary Table S1. Different taxonomic treatments of *Pyrrosia* s.l..

Reference	Study region	Genus	Infrageneric classification
Nayar and Chandra, 1965[1]	India	*Pyrrosia* Mirbel	Flocculosa group
			Heteractis group
			Mannii group
			Nayariana group
			Obovata group
			Varia group
Shing, 1983[2]	Global	*Pyrrosia* Mirbel	subgen. *Pyrrosia*
			sect. *Pyrrosia*
			ser. *Pyrrosia*
			ser. *Heteractides*
			ser. *Drakeanae*
			sect. *Dichlamys*
			ser. *Costatae*
			ser. *Molles*
			subgen. *Niphopsis*
		Drymoglossum C.Presl	
		Saxiglossum Ching	
Hovenkamp, 1986[3]	Global	*Pyrrosia* Mirbel	P. african-group
			P. costata-group
			P. porosa-group
			P. sheareri-group
			P. lingua-group
			P. albicans-group
			P. angustata-group
			P. confluens-group
			P. lanceolata-group
			P. piloselloides-group
Yang, 2012[4]	China	*Pyrrosia* Mirbel	subgen. *Pyrrosia*
			sect. *Nummulariifoliae*
			sect. *Pyrrosia*
			sect. *Lanceolatae*
			subgen. *Porosae*
			sect. *Drakeanae*
			sect. *Porosae*
			sect. *Costatae*
		Drymoglossum C. Presl	
		Saxiglossum Ching	
Zhou et al. 2017[5]	Global	*Pyrrosia* Mirbel	*Pyrrosia* clade

			Niphopsis clade<br>*Galeoglossa* subclade<br>*Heteractides* subclade<br>*Niphopsis* subclade<br>*Saxiglossum* subclade
			Neoniphopsis clade<br>*Candollea* subclade<br>*Drymoglossum* subclade<br>*Fallax* subclade<br>*Foveolata* subclade<br>*Niphobolus* subclade<br>*Serpens* subclade
			Niphobolus clade<br>*Dichlamys* subclade<br>*Drakeanae* subclade<br>*Mannii* subclade<br>*Polydactyla* subclade
		Hovenkampia Li Bing Zhang & X. M. Zhou	
Vasques et al. 2017[6]	Global	*Pyrrosia* Mirbel	subg. *Pyrrosia*<br>subg. *Lunae*<br>subg. *Neoniphopsis*<br>subg. *Niphobolus*<br>subg. *Niphopsis*<br>subg. *Solis*

Supplementary Table S2. Taxa, voucher and GenBank accession numbers for the sequences used in this study. Country abbreviations: CN, China; JP, Japan; ID, Indonesia; KE, Kenya; MM, Myanmar; SG, Singapore; VIE, Vietnam. † represents samples used in phylogenetic analyses for supplementary figure S1. *represents sequences download from GenBank.

Taxon	Voucher number and locality	GenBank accession numbers				
		*rbc*L	*psb*A-*trn*H	*rps*4+*rps*4-*trn*S	*mat*K	*LEAFY*
Pyrrosia abbreviata (Zoll. & Moritzi) Hovenkamp	ID, Wei et al. slws-16 (PE)	KY632744	KY632842	KY633159	KY632940	KY633035, KY633036
Pyrrosia abbreviata (Zoll. & Moritzi) Hovenkamp	ID, Zhang et al. 2923 (PE)	KY632745	KY632843	KY633160	KY632941	KY633037, KY633038
Pyrrosia abbreviata (Zoll. & Moritzi) Hovenkamp †	MM, Zhang X.C. 6726 (PE)	KY632746	KY632844	KY633161	KY632942	KY633039
Pyrrosia adnascens (Sw.) Ching	Hainan, CN, Wei & Wei wxp066 (PE)	KY632747	KY632845		KY632943	
Pyrrosia adnascens (Sw.) Ching †	Hainan, CN, Zhang et al. 5502 (PE)	KY632748	KY632846	KY633162	KY632944	
Pyrrosia adnascens (Sw.) Ching	Yunnan, CN, Li Z.Y. lzy08 (IMD)	KY632749	KY632847	KY633163	KY632945	
Pyrrosia adnascens (Sw.) Ching	VIE, Schneider & Liu 2011v92 (SZG)	KY632750	KY632848	KY633164	KY632946	KY633040
Pyrrosia adnascens (Sw.) Ching	ID, Wei et al. 386 (PE)	KY632751	KY632849	KY633165	KY632947	
Pyrrosia angustata (Sw.) Ching †	VIE, Wei Q. wq004 (IMD)	KY632752	KY632850	KY633166	KY632948	KY633041

Pyrrosia angustissima (Giesenh. ex Diels) Tagawa & K. Iwats.	Xizang, CN, Li Z.Y. 003 (PE)	KY632753	KY632851	KY633167	KY632949	
Pyrrosia angustissima (Giesenh. ex Diels) Tagawa & K. Iwats.	Chongqing, CN, Qi X.P. qi09-15 (PE)	KY632754	KY632852	KY633168	KY632950	KY633042
Pyrrosia angustissima (Giesenh. ex Diels) Tagawa & K. Iwats. †	Chongqing, CN, Liu H.M. cq308 (PE)	KY632755	KY632853	KY633169	KY632951	KY633043, KY633044, KY633045, KY633046
Pyrrosia assimilis (Baker) Ching	Hunan, CN, Zhang et al. 6153 (PE)	KY632756	KY632854	KY633170	KY632952	KY633047
Pyrrosia assimilis (Baker) Ching †	Jiangxi, CN, Wei & Li jgs043 (PE)	KY632757	KY632855	KY633171	KY632953	KY633048, KY633049, KY633050
Pyrrosia assimilis (Baker) Ching		DQ164464*	KJ528481*	DQ164495*		
Pyrrosia bonii (Christ ex Giesenh.) Ching †	Guangxi, CN, Guangxi Coll. Team 4212 (PE)	KY632758	KY632856	KY633172	KY632954	KY633051
Pyrrosia bonii (Christ ex Giesenh.) Ching	Guizhou, CN, Guo et al. 7074 (PE)	KY632759	KY632857	KY633173	KY632955	KY633052
Pyrrosia bonii (Christ ex Giesenh.) Ching	Guizhou, CN, Zhang et al. 7234 (PE)	KY632760	KY632858	KY633174	KY632956	
Pyrrosia calvata (Baker) Ching	Guangxi, CN, Lang K.Y. ly s.n. (PE)	KY632761	KY632859	KY633175	KY632957	KY633053
Pyrrosia calvata (Baker) Ching †	cultivated, CN, Wei X.P. 041501 (IMD)	KY632762	KY632860	KY633176	KY632958	KY633054
Pyrrosia calvata (Baker) Ching	Gansu, CN, Baishuijing Coll. Team 3729 (PE)					KY633055
Pyrrosia caudifrons Ching, Boufford & K.H. Shing †	Hubei, CN, Sun & Wu 022 (PE)	KY632763	KY632861	KY633177	KY632959	KY633056
Pyrrosia christii (Giesenh.) Ching †		DQ164465*		DQ164496*		

Pyrrosia costata (Wall. ex C. Presl) Tagawa & K. Iwats. †	Yunnan, CN, Li Z.Y. lzy01 (IMD)	KY632764	KY632862	KY633178	KY632960	KY633057
Pyrrosia costata (Wall. ex C. Presl) Tagawa & K. Iwats.	Yunnan, CN, Zhang & Dong 1429-1 (PE)	KY632765	KY632863	KY633179	KY632961	KY633058
Pyrrosia costata (Wall. ex C. Presl) Tagawa & K. Iwats.	Yunnan, CN, Zhang & Dong 1429-2 (PE)	KY632766	KY632864	KY633180	KY632962	KY633059
Pyrrosia davidii (Giesenh. ex Diels) Ching †	Beijing, CN, Wei X.P. wxp215 (PE)	KY632768	KY632866	KY633181	KY632963	KY633060, KY633061
Pyrrosia davidii (Giesenh. ex Diels) Ching	Beijing, CN, Wei R. wr174 (PE)	KY632769	KY632867	KY633182	KY632964	KY633062
Pyrrosia davidii (Giesenh. ex Diels) Ching	Shaanxi, CN, Wei R. wr039 (PE)	KY632770	KY632868	KY633183	KY632965	KY633063
Pyrrosia drakeana (Franch.) Ching †	Xizang, CN, Zhang et al. 936 (PE)	KY632771	KY632869	KY633184	KY632966	KY633064
Pyrrosia drakeana (Franch.) Ching	Yunnan, CN, Xu C.D. a252 (PE)	KY632772	KY632870	KY633185	KY632967	KY633065
Pyrrosia drakeana (Franch.) Ching	Sichuan, CN, Zhang X.C. 5896 (PE)	KY632773	KY632871	KY633186	KY632968	KY633066
Pyrrosia eberhardtii (Christ) Ching †	Yunnan, CN, Dong S.Y. 816 (PE)	KY632774	KY632872	KY633187	KY632969	KY633067
Pyrrosia eleagnifolia (Bory) Hovenkamp †		DQ642166*		DQ642205*		
Pyrrosia ensata Ching ex K.H. Shing †	Yunnan, CN, QTP Coll. Team 9235 (PE)	KY632767	KY632865	KY633188		
Pyrrosia fengiana Ching †	Yunnan, CN, Wu C. ch0904009 (PE)	KY632775	KY632873	KY633189	KY632970	KY633068
Pyrrosia flocculosa (D. Don) Ching †	Yunnan, CN, Zhang X.C. 2927 (PE)	KY632776	KY632874	KY633190	KY632971	KY633069, KY633070

Pyrrosia flocculosa (D. Don) Ching	Xizang, CN, Li & Li 14160 (PE)	KY632777	KY632875	KY633191	KY632972	
Pyrrosia flocculosa (D.Don) Ching	Yunnan, CN, Zhao J.C. 5948 (PE)	KY632778	KY632876	KY633192	KY632973	KY633071
Pyrrosia foveolata (Alston) C.V. Morton †		DQ642167*		DQ642206*		
Pyrrosia gralla (Giesenh.) Ching	Fujian, CN, Wei X.P. wys11 (IMD)	KY632779	KY632877	KY633193	KY632974	KY633072, KY633073, KY633074
Pyrrosia gralla (Giesenh.) Ching †	Yunnan, CN, Zhang X.C. 5667 (PE)	KY632780	KY632878	KY633194	KY632975	KY633075
Pyrrosia gralla (Giesenh.) Ching	Sichuan, CN, Zhang X.C. 5913 (PE)	KY632781	KY632879	KY633195	KY632976	
Pyrrosia hastata (Thunb. ex Houtt.) Ching †		AB575296*	AB575906*			
Pyrrosia heteractis (Mett. ex Kuhn) Ching	Yunnan, CN, Zhang et al. 6264 (PE)	KY632782	KY632880	KY633196	KY632977	
Pyrrosia heteractis (Mett. ex Kuhn) Ching †	Xizang, CN, Zhang X.C. 5052 (PE)	KY632783	KY632881	KY633197	KY632978	KY633076
Pyrrosia kinabaluensis Hovenkamp †	ID, Wei et al. 457 (PE)	KY632784	KY632882	KY633198	KY632979	KY633077, KY633078, KY633079
Pyrrosia laevis (J. Sm. ex Bedd.) Ching †	Yunnan, CN, Shing et al. 6769 (PE)	KY632785	KY632883	KY633199		KY633080
Pyrrosia lanceolata (L.) Farw. †	Xizang, CN, Li Z.Y.1924 (PE)	KY632786	KY632884	KY633200	KY632980	KY633081
Pyrrosia lanceolata (L.) Farw.	Yunnan, CN, Li Z.Y. lzy02 (IMD)	KY632787	KY632885	KY633201	KY632981	KY633082
Pyrrosia linearifolia (Hook.) Ching †	JP, Shing K.H. 78 (PE)	KY632788	KY632886	KY633202	KY632982	

Pyrrosia lingua (Thunb.) Farw.	Fujian, CN, Wei X.P. wys120206 (IMD)	KY632789	KY632887	KY633203	KY632983	
Pyrrosia lingua (Thunb.) Farw. †	Guangdong, CN, Wei et al. wxp009 (PE)	KY632790	KY632888	KY633204		KY633083, KY633084, KY633085, KY633086
Pyrrosia lingua (Thunb.) Farw.	Guangdong, CN, Wang F.G. 1341 (PE)					KY633087
Pyrrosia lingua (Thunb.) Farw.	Sichuan, CN, Zhu et al. 1268 (PE)					KY633088
Pyrrosia lingua (Thunb.) Farw.	Sichuan, CN, Li D. 23 (PE)					KY633089
Pyrrosia lingua (Thunb.) Farw.	Yunnan, CN, Zhang & Liu 6048 (PE)					KY633090
Pyrrosia longifolia (Burm. f.) C.V. Morton †	ID, Zhang et al. 082 (PE)	KY632791	KY632889	KY633205	KY632984	KY633091, KY633092, KY633093, KY633094
Pyrrosia longifolia (Burm. f.) C.V. Morton	ID, Wei et al. 2446 (PE)	KY632792	KY632890	KY633206	KY632985	KY633095
Pyrrosia longifolia (Burm. f.) C.V. Morton	SG, Zhang X.C. 6540 (PE)	KY632793	KY632891	KY633207	KY632986	KY633096, KY633097
Pyrrosia mannii (Giesenh.) Ching	Yunnan, CN, Zhao J.C. zjc s.n. (PE)	KY632794	KY632892	KY633208	KY632987	KY633098
Pyrrosia mannii (Giesenh.) Ching †	Xizang, CN, Zhang X.C. 5311 (PE)	KY632795	KY632893	KY633209	KY632988	KY633099
Pyrrosia mannii (Giesenh.) Ching	Yunnan, CN, Qi X.P. q088 (PE)	KY632796	KY632894	KY633210	KY632989	
Pyrrosia martini (Christ) Ching †	Jiangxi, CN, Yang & Oyang 008 (PE)	KY632797	KY632895	KY633211	KY632990	KY633100, KY633101, KY633102, KY633103
Pyrrosia niphoboloides (Luerss.) M.G. Price †		DQ642168*		DQ642207* (*rps*4)		
Pyrrosia nuda (Giesenh.) Ching †	Yunnan, CN, Xu C.D. 29958 (PE)	KY632798	KY632896	KY633212	KY632991	KY633104

Pyrrosia nuda (Giesenh.) Ching	Yunnan, CN, Li Z.Y. lzy04 (IMD)	KY632799	KY632897	KY633213	KY632992	
Pyrrosia nudicaulis Ching †	Yunnan, CN, Zhang et al. 6261 (PE)	KY632800	KY632898	KY633214	KY632993	KY633105
Pyrrosia nudicaulis Ching	Yunnan, CN, Zhang et al. 6265 (PE)	KY632801	KY632899	KY633215	KY632994	
Pyrrosia nummulariifolia (Sw.) Ching	ID, Wei et al. slws-08 (PE)	KY632802	KY632900	KY633216	KY632995	KY633106, KY633107, KY633108
Pyrrosia nummulariifolia (Sw.) Ching	Yunnan, CN, Shui et al. 80085 (PE)	KY632803	KY632901	KY633217	KY632996	KY633109
Pyrrosia nummulariifolia (Sw.) Ching †	Yunnan, CN, Li Z.Y. lzy03 (IMD)	KY632804	KY632902	KY633218	KY632997	KY633110, KY633111, KY633112, KY633113, KY633114
Pyrrosia oblonga Ching †	Guangxi, CN, 061 (PE)	KY632805	KY632903	KY633219	KY632998	KY633115
Pyrrosia penangiana (Hook.) Holtt. †	ID, Wei et al. 3014 (PE)	KY632806	KY632904	KY633220	KY632999	KY633116, KY633117, KY633118, KY633119
Pyrrosia petiolosa (Christ) Ching †	Sichuan, CN, He et al. 6735 (PE)	KY632807	KY632905	KY633221	KY633000	KY633120
Pyrrosia petiolosa (Christ) Ching	Beijing, CN, Wei X.P. wxp217 (PE)	KY632808	KY632906	KY633222	KY633001	KY633121
Pyrrosia petiolosa (Christ) Ching	Shaanxi, CN, Wu C. ch081007 (PE)	KY632809	KY632907	KY633223	KY633002	
Pyrrosia petiolosa (Christ) Ching	Shaanxi, CN, Wei R. wr035 (PE)					KY633122
Pyrrosia piloselloides (L.) M.G. Price	Hainan, CN, Schneider et al. 5535 (PE)	KY632810	KY632908	KY633224	KY633003	
Pyrrosia piloselloides (L.) M.G. Price	Hainan, CN, Zhang X.C. 5549 (PE)	KY632811	KY632909	KY633225	KY633004	KY633123

Pyrrosia piloselloides (L.) M.G. Price †	Hainan, CN, Wei & Wei wxp060 (PE)	KY632812	KY632910	KY633226	KY633005	KY633124
Pyrrosia polydactyla (Hance) Ching	Taiwan, CN, Lu P.F. 21867 (PE)	KY632813	KY632911	KY633227	KY633006	KY633125
Pyrrosia polydactyla (Hance) Ching †	Taiwan, CN, Liu Y.C. qy1 (PE)	KY632814	KY632912	KY633228	KY633007	KY633126, KY633127
Pyrrosia polydactyla (Hance) Ching	Taiwan, CN, Lu P.F. 21430 (PE)	KY632815	KY632913	KY633229	KY633008	KY633128
Pyrrosia porosa (C. Presl) Hovenkamp	Fujian, CN, Wei X.P. wys10 (IMD)	KY632816	KY632914	KY633230	KY633009	KY633129, KY633130, KY633131, KY633132, KY633133, KY633134
Pyrrosia porosa (C. Presl) Hovenkamp †	Yunnan, CN, Zhang et al. 6223 (PE)	KY632817	KY632915	KY633231	KY633010	KY633135, KY633136
Pyrrosia porosa (C. Presl) Hovenkamp	Yunnan, CN, Shui et al. 80900 (PE)	KY632818	KY632916	KY633232	KY633011	KY633137
Pyrrosia porosa (C. Presl) Hovenkamp	P. porosa DQ164466	DQ164466*		DQ164497*		
Pyrrosia rasamalae (Racib.) K.H. Shing †	ID, Wei et al. 3019 (PE)	KY632819	KY632917	KY633233	KY633012	KY633138, KY633139, KY633140
Pyrrosia rasamalae (Racib.) K.H. Shing	ID, Zhang X.C. 8018-2 (PE)	KY632820	KY632918	KY633234	KY633013	KY633141
Pyrrosia rupestris Ching †		AY362558*		AY362623*		
Pyrrosia samarensis (C. Presl) Ching †		DQ642170*		DQ642209*		
Pyrrosia schimperiana (Mett. ex Kuhn) Alston	KE, Liu et al. 27233 (PE)	KY632821	KY632919	KY633235	KY633014	KY633142
Pyrrosia schimperiana (Mett. ex Kuhn) Alston †	KE, Liu et al. 27327 (PE)	KY632822	KY632920	KY633236	KY633015	KY633143
Pyrrosia schimperiana (Mett. ex Kuhn) Alston	KE, Liu et al. 27272 (PE)	KY632823	KY632921	KY633237	KY633016	KY633144, KY633145

Pyrrosia serpens (G. Forster) Ching †		DQ164471*		DQ164503*		
Pyrrosia sheareri (Baker) Ching †	Chongqing, CN, Three Gorges Coll. Team 1800 (PE)	KY632824	KY632922	KY633238	KY633017	KY633146
Pyrrosia sheareri (Baker) Ching	cultivated, CN, Wei X.P. 2016041502 (PE)	KY632825	KY632923	KY633239	KY633018	KY633147
Pyrrosia sheareri (Baker) Ching	Hunan, CN, Liu H.M. a217 (PE)	KY632826	KY632924	KY633240	KY633019	KY633148
Pyrrosia stenophylla (Bedd.) Ching †	Xizang, CN, Li Z.Y.l1740 (PE)	KY632827	KY632925	KY633241	KY633020	
Pyrrosia stenophylla (Bedd.) Ching	Xizang, CN, Zhang X.C. 3959 (PE)	KY632828	KY632926	KY633242	KY633021	
Pyrrosia stigmosa (Sw.) Ching †		JX103716*		JX103758*		
Pyrrosia subfurfuracea (Hook.) Ching	Yunnan, CN, Wei & Wei wxp185 (PE)	KY632829	KY632927	KY633243	KY633022	KY633149
Pyrrosia subfurfuracea (Hook.) Ching †	Xizang, CN, Li Z.Y.1925 (PE)	KY632830	KY632928	KY633244	KY633023	KY633150
Pyrrosia subfurfuracea (Hook.) Ching	Yunnan, CN, Liu & Zhang 7760 (PE)	KY632831	KY632929	KY633245	KY633024	KY633151
Pyrrosia subtruncata Ching †	Yunnan, CN, Zhang & Li 5621 (PE)	KY632832	KY632930	KY633246	KY633025	KY633152
Pyrrosia tonkinensis (Giesenh.) Ching †	Hainan, CN, Wei & Wei wxp058 (PE)	KY632833	KY632931	KY633247	KY633026	KY633153
Pyrrosia tonkinensis (Giesenh.) Ching	Yunnan, CN, Li Z.Y. lzy05 (IMD)	KY632834	KY632932	KY633248	KY633027	
Pyrrosia tonkinensis (Giesenh.) Ching	Guizhou, CN, Guo et al. 7106 (PE)	KY632835	KY632933	KY633249	KY633028	KY633154
Pyrrosia tonkinensis (Giesenh.) Ching	Guizhou, CN, Guo et al. 7233 (PE)	KY632836	KY632934	KY633250	KY633029	KY633155

Pyrrosia tonkinensis (Giesenh.) Ching	Hainan, CN, Zhang et al. 1568 (PE)	KY632837	KY632935	KY633251	KY633030	KY633156
Pyrrosia transmorrisonensis (Hayata) Ching †	Taiwan, CN, Liu Y. C. ys1 (PE)	KY632838	KY632936	KY633252	KY633031	KY633157, KY633158
Platycerium bifurcatum (Cav.) C. Chr. †	cultivated, CN, Wei X.P. 41503 (PE)	KY632839	KY632937	KY633253	KY633032	MF450483, MF450484
Platycerium bifurcatum (Cav.) C. Chr.	cultivated, CN, Zhao H.J. T109 (PE)					MF450485, MF450486
Platycerium coronarium (O.F. Müll.) Desv.	VIE, Wei Q. wq002 (IMD)	KY632840	KY632938	KY633254	KY633033	
Platycerium wallichii Hook.	MM, Zhang X.C. 6730 (PE)	KY632841	KY632939	KY633255	KY633034	
Loxogramme duclouxii Christ†	CN, Wei X.P. wxp201721 (IMD)	MF450443	MF450453	MF450464	MF450473	
Loxogramme lankokiensis (Rosenst.) C. Chr. †	CN, Wei X.P. s.n. (PE)	MF450444	MF450454	MF450465	MF450474	MF450487, MF450488
Loxogramme grammitoides (Baker) C. Chr. †	CN, Wei X.P. wxp201722 (IMD)	MF450445	MF450455	MF450466	MF450475	MF450489
Aglaomorpha fortunei (Kunze ex Mett.) Hovenkamp & S.Linds. †	CN, Bai Y.J. JXPX01 (IMD)	MF450446	MF450456	MF450467	MF450476	MF450490, MF450491
Arthromeris lehmannii (Mett.) Ching†	CN, Wei X.P. wxp201719 (IMD)	MF450447	MF450457	MF450468		MF450492
Goniophlebium chinense (Christ) X.C. Zhang†	CN, Wei X.P. wxp201718 (IMD)	MF450448	MF450459	MF450469	MF450478	
Lemmaphyllum rostratum (Bedd.) Tagawa†	CN, Wei X.P. &Wei R. wxp108 (PE)	KX891375*	MF450458	KX891407*	MF450477	KX874653*
Lepisorus marginatus Ching						JQ806670*
Tricholepidium normale (D. Don) Ching						JQ806684*,JQ806686*

Polypodium glycyrrhiza D.C. Eaton†	chloroplast, partial genome	KP136832*	KP136832*	KP136832*	KP136832*	
Microgramma baldwinii Brade						KM114152*
Micropolypodium okuboi (Yatabe) Hayata†	CN, Liu H.M. s.n. (PE)	MF450449	MF450460	MF450470	MF450479	MF450493
Calymmodon asiaticus Copel. †	CN, Zhang X.C. 3531 (PE)	MF450450	MF450461		MF450480	
Davallia repens (L. f.) Kuhn†	CN, Wei X.P. &Wei R. wxp092 (PE)	MF450451	MF450462	MF450471	MF450481	
Davallia trichomanoides Blume†	ID, Wei et al. 006 (PE)	MF450452	MF450463	MF450472	MF450482	MF450494, MF450495

Supplementary Table S3. Specimens used for morphological characters observation.

Taxon	Specimens
Pyrrosia abbreviata (Zoll. & Moritzi) Hovenkamp	Wei et al. slws-16 (PE), Zhang et al. 2923 (PE), Zhang X.C. 6726 (PE), Sun H.F. 6806 (PE), Sun H.F. 7157 (PE)
Pyrrosia adnascens (Sw.) Ching	Wei et al. 386 (PE), Wei & Wei wxp066 (PE), Zhang et al. 5502 (PE), Li Z.Y. lzy08 (IMD), Schneider H. & Liu H.M. 2011v92 (SZG), Chen S.Q. 7087(PE), Ye G.D. 2259(PE), Zhang et al 1422(PE), Cai K.H. 182 (PE), Liang X.R. 62319 (PE), Liang X.R. 66099 (PE), Liang X.R. 65070 (PE), Chen N.Q. 43553 (PE), Chen S.Q. 11336 (PE), Chow et al. 78965 (PE), Lau S.K. 26648 (PE), Liang H.Y. 64984 (PE), Wang C. 36757 (PE), Liang H.Y. 65070 (PE), Zhang Q.Q. 520 (PE), Zhou N.S. 2560 (PE), Zhou N.S. T23 (PE), Ye G.D. 2259 (PE), Ye G.D. & Cai G.L. (PE), Cai G.L. 157 (PE), Qiu P.X. 7833 (PE), Anonymous s.n. (PE), Y.Kudo et al. 15995 (PE), K.K.Tsoong 945 (PE), Wang Z.R. C622-2 (PE), Wang Z.R. C622-1 (PE), Wang Z.R. C622-1 (PE), Liang K. 68009 (PE), Xia Z.D. 81021 (PE), Hu S.Y. 11220 (PE), Hu S.Y. 6573 (PE), Hu S.Y. 9611 (PE), Hu S.Y. 9710 (PE), Hu S.Y. 8995 (PE), Hu S.Y. 5547A (PE), Hu S.Y. 9844 (PE), Hu S.Y. 6709 (PE), Hu S.Y. 9886 (PE), Hu S.Y. 12861 (PE), Hu S.Y. 9683 (PE), Hu S.Y. 11955 (PE), Hu S.Y. 5174 (PE), Hu S.Y. 9518 (PE), Hu S.Y. 20640 (PE), Ko S.P. 31876 (PE), Chun S.P. 41758 (PE), Liang N.K. 60708 (PE), Tsang W.T. 25783 (PE), Zhang & Dong 1422 (PE), Cai K.F. 510 (PE), Yu T.T. 1032 (PE), Yu T.T. 1032 (PE), Chow K.S. 80349 (PE), Li M.K. 1496 (PE), 236-6 Team 1567 (PE), 236-6 Team 1693 (PE), 236-6 Team 1693 (PE), Lau S.K. 4937 (PE), Lau S.K. 3512 (PE), Lau S.K. 6319 (PE), Tsang W.T. 255 (PE), Dong S.Y. 433 (PE), Dong S.Y. 739 (PE), Dong S.Y. 911 (PE), Dong S.Y. 19 (PE), Sun H.F. 403 (PE), Sun H.F. 44 (PE), Sun H.F. s.n. (PE), T.Murakami et al. s.n. (PE), C.F.van Beusekom et al. 574 (PE), Hideo Tabata et al. 11194 (PE), Hideo Tabata et al. 11093 (PE), B.Bartholomew et al. 3800 (PE), Wang C. 36296 (PE), Wang C. 34347 (PE), Wang C. 34395 (PE), Wang C. 36110 (PE), Ko S.P. 52285 (PE), McClure F.A. 9644 (PE), Tsang W.T. 15915 (PE), Tsang W.T. 15915 (PE), Tsui T.M. 61 (PE), Tsang W.T. 16212 (PE), Lei C.I. 813 (PE), Lei C.I. 813 (PE), Levine 1969 (PE), G.W.Groff.C.O. 945 (PE), Tsiang Y. 633 (PE), Chun W.Y. 6246 (PE), Chung H.H. 1391 (PE), Anonymous s.n. (PE), Tsoong K.K. 945 (PE), Liou T.N. 897 (PE), Chun N.K. 41758 (PE), Chun N.K. 41694 (PE), Chun N.K. 41692 (PE), Ko S.P. 51559 (PE), Y.Tsiang 0205 (PE),

	Anonymous 31062 (PE), Tsang 16535 (PE), Lei C.I. 813 (PE), Williams R.S. s.n. (PE), Alston A.H.G. 12630 (PE), Wang Y.L. 476 (PE), Makino J. s.n. (PE), Zhang & Li 219 (PE), Zhang & Li 014 (PE), Wuzhishan Fern Survey 402 (PE), Wuzhishan Fern Survey 402 (PE), Wuzhishan Fern Survey 476 (PE), Wuzhishan Fern Survey 160 (PE), Zhang & Li 97 (PE), Zhang & Li 050 (PE)
Pyrrosia angustata (Sw.) Ching	Wei Q. wq004 (IMD), Burchaw L.W. 12 (PE), Sun H.F. 87 (PE), Sun H.F. 557 (PE), Keng L.B. 7036 (PE), Sun H.F. s.n. (PE), Anonymous 6993 (PE), Anonymous s.n. (PE), Anonymous 7116 (PE), Anonymous 6930 (PE), Bot N.Y. 500 (PE)
Pyrrosia angustissima (Giesenh. ex Diels) Tagawa & K. Iwats.	Li Z.Y. 003 (PE), Qi X.P. qi09-15 (PE), Sun & Wu 032 (PE), Liu Z.Y. 19439 (PE), Zhang X.C. 3362 (PE), Zhang X.C. & Liu Z.Y. 2722 (PE), Baishuijiang Team 5006 (PE), Baishuijiang Team 1765 (PE), Threegorges Team 0361 (PE), Threegorges Team 0361 (PE), Threegorges Team 0317 (PE), Threegorges Team 0317 (PE), Threegorges Team 0558 (PE), Threegorges Team 0558 (PE), Wang T. FSB-792 (PE)
Pyrrosia assimilis (Baker) Ching	Zhang et al. 6153 (PE), Wei & Li jgs043 (PE), Zhang et al. 1997 (PE), Wu S.F. 7056(PE), Anonymous 0492 (PE), Zhang S.Y. 3838 (PE), Zhang S.Y. 3838 (PE), Anonymous 30193 (PE), Anonymous 27598 (PE), Anonymous 0158 (PE), Wang N.J. 0711 (PE), Xiong Y.G. 09895 (PE), 236 Team 1030 (PE), Zhang X.C. et al. 1917 (PE), Yue J.S et al. 2656 (PE), Hu Q.M. 5335 (PE), Nie 5675 (PE), Yue J.S et al. 4841 (PE), Xiangxi Team 0323 (PE), Wu S.F. et al 7056 (PE), Census team 307 (PE), Luo L.B. 60 (PE), Liu L.H. 1802 (PE), Liu Q.X. 4021 (PE), Liu L.H. & He G.Z. 016261 (PE), Liu L.H. 10109 (PE), Liu L.H. 1661 (PE), Tsiang Y. 6851 (PE), Tsoong K.K. 3204 (PE), Chao H.C. 1784 (PE), Fu & Zhang 96015 (PE), Ko S.P. 50644 (PE), Chun N.K. 40826 (PE), Kwak S.P. 80155 (PE), Tsui T.M. 55 (PE), Tsui T.M. 55 (PE), Tsang W.T. 20896 (PE), Tsang W.T. 20896 (PE), Tsang W.T. 26077 (PE), Tsang W.T. 26223 (PE), Cheo H.C. 41 (PE), Tsang W.T. 21356 (PE), Tsang W.T. 21356 (PE), Lau S.K. 2542 (PE), Tsoong K.K. 3204 (PE), Liu H.M. GX069 (PE), Liu H.M. GX151 (PE), Zhang X. C. et al. 4129 (PE), Zhou & Qin 675 (PE), Yan Y.H. 2339 (PE), Yan Y.H. 110021 (PE), Threegorges Team 0582 (PE), He G.S. 9735 (PE), Zhang X.C. et al. 6761 (PE), Guangxi Team 0898 (PE)
Pyrrosia bonii (Christ ex Giesenh.) Ching	Zhang et al. 7234 (PE), Guo et al. 7074 (PE), Guangxi Coll. Team 4212 (PE), Li et al 7364 (PE), Tsoong K.K. 1833 (PE), J.Cavalerie 2875 (PE), K.K.Tsoong 1833 (PE), Li Z.T. 602636 (PE), Qin H.C. 6478 (PE), Qin H.C. 5432 (PE), Qin H.C. 5432 (PE), Guangxi Team 2667 (PE), Guangxi Team 1018 (PE), Guangxi Team 4592 (PE),

	Guangxi Team 2677 (PE), Guangxi Team 0789 (PE), Guangxi Team 0785 (PE), Guangxi Team 0572 (PE), Guangxi Team 0436 (PE), Guangxi Team 4212 (PE)
Pyrrosia calvata (Baker) Ching	Lang K.Y. ly s.n. (PE), Wei X.P. 041501 (IMD), Baishuijing Coll. Team 3729 (PE), Dai T.L. 104590 (PE), Zhang X.C. & Liu Z.Y. 2694 (PE), Baise Team 1554 (PE), Anonymous 32 (PE), S.P.Ko 55644 (PE), Shan R.H 856 (PE), Wang Tso-Pin 9940 (PE), Anonymous 19025 (PE), Anonymous 19026 (PE), Liu S.E. 018462 (PE), Liu S.E. 018462 (PE), Liu S.E. 018471 (PE), Xu Y.C. 3 (PE), Xu Y.C. 3 (PE), Zhong B.Q. & Kuang K.R. 146 (PE), Zhang L.Y. 64-10 (PE), Cai X.T. 58-8522 (PE), Wu S.G. 359 (PE), Anonymous 074 (PE), Y.Tsiang 12284 (PE), Anonymous s.n. (PE), H.T.Tsai 51883 (PE), H.T.Tsai 61757 (PE), H.T.Tsai 53252 (PE), Feng K.M. 11420 (PE), Ching R.C. 154 (PE), Dai T.L. 107167 (PE), Zhang & Zhang 3460 (PE), Kong X.X. 5311 (PE), Yu T.H. 593 (PE), Tsiang Y. 9485 (PE), Tsiang Y. 9249 (PE), Tsai H.T. 52371 (PE), Tsai H.T. 52346 (PE), Zhang & Liu 2694 (PE), Zhang & Shi 994 (PE), Liu B.R. 871093 (PE), Zhou & Chen 899 (PE), Qi X.P. Q053 (PE), Shui Y.M. 80887 (PE), Liu Z.Y. 184488 (PE), Liu Z.Y. 184432 (PE)
Pyrrosia caudifrons Ching, Boufford & K.H. Shing	Sun & Wu 022 (PE), Shing K.H. 519 (PE), Guan K.J. 2330 (PE), Li H.J. 8393 (PE), Li H.J. 8393 (PE), Li H.J. 6840 (PE), B.Bartholomew et al. 1159 (PE), Xing G.X. & Lang X.Y. 519 (PE), Xing G.X. & Lang X.Y. 519 (PE), Xing G.X. & Lang X.Y. 525 (PE), Xing G.X. & Lang X.Y. 525 (PE), Xing G.X. & Lang X.Y. 1163 (PE), Guang K.J. et al. 2330 (PE), Guang K.J. et al. 2330 (PE), Kong X.R. 3691 (PE), Anonymous 8651 (PE), Anonymous 8669 (PE), Anonymous 8355 (PE), Guan Z.T. 8426 (PE), Wang Y.M. 6124 (PE), Beijing Team 002638 (PE), Liu B.R. 8310070 (PE), Guang K.J. et al. 2330 (PE)
Pyrrosia costata (Wall. ex C. Presl) Tagawa & K. Iwats.	Li Z.Y. lzy01 (IMD), Zhang & Dong 1429-1 (PE), Zhang & Dong 1429-2 (PE), Zhang X.C. 2927 (PE), Li B.S. 03976 (PE), Wu S.K. 360 (PE), Dong Q.Z. 796 (PE), Dong Q.Z. 803 (PE), Li B.S. & Chen S.Z. 03976 (PE), Tao D.D. 0412 (PE), Jiang N. et al. 8130 (PE), Wu S.G. 360 (PE), M.K.Li 1495 (PE), Anonymous 6793 (PE), Hideo Tabata et al. 1495 (PE), Zhao J.C. s.n. (PE), Zhang X.C. et al. 1429 (PE), Anonymous s.n. (PE), Zhang X.C. 2927 (PE), Zhang X.C. 2927 (PE), Chen W.L. 10950 (PE), Chen W.L. 10950 (PE), Chen W.L. 10950 (PE), Chen W.L. 10950 (PE), Chen W.L. 11065 (PE), Chen W.L. 11065 (PE), Chen W.L. 11065 (PE), Chen S.Z. & Li B.S. 03297 (PE), Chen S.Z. & Li B.S. 03297 (PE), Chen S.Z. & Li B.S. 01708 (PE), Chen S.Z. & Li B.S. 01708 (PE), Chen S.Z. & Li B.S.02351 (PE), Chen S.Z. & Li B.S.02351 (PE), Dianxi Team10778 (PE), Li M.K.1495 (PE)

Pyrrosia davidii (Giesenh. ex Diels) Ching	Wei X.P. wxp215 (PE), Wei R. wr174 (PE), Wei R. wr039 (PE), Hou X.Y. 12758 (PE), Lin et al. 34 (PE), Anonymous 22914A (PE), T.H.Shih 54 (PE), Anonymous 0149 (PE), Henan Team L232 (PE), Henan Team L232 (PE), Henan Team L232 (PE), Guan K.J. & Dai T.L. 1896 (PE), Guan K.J. & Dai T.L. 1896 (PE), Guan K.J. & Dai T.L. 975 (PE), Guan K.J. & Dai T.L. 975 (PE), Anonymous 0510 (PE), Anonymous 0510 (PE), Anonymous 35159 (PE), Yu H.J. 1816 (PE), Yu H.J. 1817 (PE), Anonymous 209 (PE), Anonymous 234 (PE), Gao S.Z. 1420 (PE), Gao S.Z. 87 (PE), Guo B.Z. 112 (PE), Wang Z.B. 16582 (PE), Fu S.J. 6198 (PE), Wang Z.B. 15386 (PE), Fu S.J. 4661 (PE), Jiang N. & Jin C.L. 00214 (PE), Wang J.W. 185 (PE), Zhang Z.Y. 16001 (PE), Wang Z.B. 16364 (PE), Xu Y.P. 635 (PE), Xu Y.P. 626 (PE), Zhang Z.W. 1496 (PE), P.C.Tsoong 544 (PE), W.Y.Hsia 4328b (PE), W.Y.Hsia 4328b (PE), K.S.Hao 4058 (PE), K.S.Hao 4098 (PE), K.S.Hao 4058 (PE), T.P.Wang 1539 (PE), T.P.Wang 1539 (PE), T.P.Wang 1539 (PE), H.W.Kung 2524 (PE), H.W.Kung 2524 (PE), H.W.Kung 3636 (PE), Liou et al. 212 (PE), Liou et al. 212 (PE), Liou et al. 98 (PE), Liou et al. 98 (PE), Liou et al. 3218 (PE), Liou et al. 3218 (PE), Liou et al. 3426 (PE), X.C.Zhang 176 (PE), X.C.Zhang 177 (PE), W.Y.Hsia 2860 (PE), W.Y.Hsia 2860 (PE), W.Y.Hsia 2860 (PE), Anonymous s.n. (PE), Y.Liu 12230 (PE), Tsi-Tang Li 507 (PE), H.W.Kung 1203 (PE), K.M.Liou 2289 (PE), K.M.Liou 2289 (PE), Cui Y.W. 1748 (PE), T.Tang 10730 (PE), Anonymous 62536 (PE), C.W.Wang 61426 (PE), C.W.Wang 62594 (PE), Wu et Yang 37213 (PE), J.sato 10160 (PE), J.sato 10160 (PE), Anonymous 61426 (PE), Liu Y 12545 (PE), C.W.Wang 62536 (PE), Wang C.T. 2742 (PE), Jin D.F. 87 (PE), C.W.Wang 60187 (PE), Anonymous 62594 (PE), Anonymous 67 (PE), Wang Z.R. C23 (PE), Liu X.Y. 1602 (PE), Wu & Yang 36801 (PE), X.C.Zhang 183 (PE), X.C.Zhang 183 (PE), Liu B. 465 (PE)
Pyrrosia drakeana (Franch.) Ching	Zhang et al. 936 (PE), Xu C.D. a252 (PE), Zhang X.C. 5896 (PE), Gao S.Z. s.n. (PE), Xie Y.T. 07428 (PE), Yang J.X. 1401 (PE), Fu K.J. 6282 (PE), Wang Z.B. 16417 (PE), Fu K.J. 4662 (PE), Yang J.X. 426 (PE), Xu Y.P. 594 (PE), Medicine Team 2032 (PE), Anonymous 3055 (PE), Huang Q. 165 (PE), T.P.Wang 1993 (PE), T.P.Wang 1993 (PE), H.W.Kung 3585 (PE), H.W.Kung 3585 (PE), H.W.Kung 3585 (PE), T.N.Liou et al. 592 (PE), T.P.Wang 6485 (PE), K.T.Fu 2858 (PE), T.N.Liou et al. 2233 (PE), T.P.Wang 6570 (PE), T.P.Wang 6570 (PE), T.N.Liou et al. 1977 (PE), Wang Z.B. 14624 (PE), He Y.Q. 642 (PE), W.Y.Hsia 5481 (PE), W.Y.Hsia 6321 (PE), W.Y.Hsia 6637 (PE), Liu K.R. 0854 (PE), Liu Y. 189 (PE), Liu Y. 189 (PE), Fu .G.X. 731 (PE),

	Shennongjia Team 22296 (PE), Shennongjia Team 22296 (PE), Guan Z.T. 6906 (PE), Wang et al. 284 (PE), K.T.Fu 2858 (PE), He Y.Q. 974 (PE), Bailongjiang Team 338 (PE), Bailongjiang Team 617 (PE), Jiang X.L. 46874 (PE), Hu & He 11671 (PE), Hu & He 11671 (PE), Jiang X.L. 34858 (PE), Jiang X.L. 10957 (PE), Jiang X.L. 11045 (PE), Yao Z.W. 4888 (PE), T.N.Liou et al. 1013 (PE), Fang W.P. 6671 (PE), Harry Smith 13012 (PE), Qu G.L. 2907 (PE), Qu G.L. 2907 (PE), Qu G.L. 3950 (PE), Qu G.L. 3950 (PE), Zheng X.J. 030171 (PE), Xie & Kong 39864 (PE), Xie & Kong 40437 (PE), Xie & Kong 40144 (PE), Song Z.P. 38074 (PE), Chuanqian Team 1080 (PE), Wang & Wang 86965 (PE), Wang & Wang 86965 (PE), Wang & Wang 86965 (PE), C.W.Wang 63590 (PE), C.W.Wang 68670 (PE), C.W.Wang 68670 (PE), C.W.Wang 68670 (PE), C.W.Wang 68234 (PE), C.W.Wang 69227 (PE), C.W.Wang 69227 (PE), C.W.Wang 69233 (PE), C.W.Wang 69233 (PE), C.W.Wang 69145 (PE), C.W.Wang 69145 (PE), H.T.Tsai 59928 (PE), Chen et al. 2147 (PE), Chen et al. 2147 (PE), Chen et al. 2147 (PE), B.Bartholomew et al. 519 (PE), B.Bartholomew et al. 18 (PE), Anonymous 33622 (PE), Guan & Wang 2993 (PE), Guan & Wang 2995 (PE), Guan & Wang 2995 (PE), Guan & Wang 2345 (PE), Guan & Wang 132 (PE), Guan & Wang 132 (PE), Kong X.X. 3939 (PE), Kong X.X. 3943 (PE), Jiang S. 9913 (PE), Anonymous 8359 (PE), Anonymous 8359 (PE), Kong X.X. 6233 (PE), Yang G.H. 59124 (PE), Dai T.L. 101568 (PE), Li X. 46078 (PE), Li X. 76826 (PE), Li X. 46671 (PE), Li X. 46810 (PE), Li X. 46626 (PE), Li X. 46584 (PE), W.Y.Hsia 6637 (PE), Li X. 46584 (PE), Anonymous 59124 (PE), Zhang & Ren 07597 (PE), Zhang & Ren 07472 (PE), Zhang & Ren 04904 (PE), Guan & Wang 2993 (PE), Yu D.J. 787 (PE), Dai T.L. 106858 (PE), Li X. 46810 (PE), Dai T.L. 103881 (PE), Dai T.L. 107441 (PE), Dai T.L. 102213 (PE), Li X. 46626 (PE), Zhou H.C. 579 (PE), Wang Z.R. 830 (PE), Qin R.C. 140 (PE), Qin R.C. 140 (PE), Zhou H.C. 8320 (PE), Fang W.P. 12848 (PE), T.C.Lee 3808 (PE), Zhu et al. 923 (PE), Kong X.X. 2535 (PE), Anonymous 14646 (PE), Zheng Z.S. 743 (PE), Anonymous 1264 (PE), Zhao et al. 150716 (PE), Jiang S. 9913 (PE), Anonymous 05279 (PE), Dai T.L. 105407 (PE), Dai T.L. 106447 (PE), Dai T.L. 106664 (PE), Dai T.L. 103881 (PE), Jiang X.L. 34858 (PE), Song Z.P. 39144 (PE), Song Z.P. 38908 (PE), Song Z.P. 38908 (PE), Song Z.P. 38079 (PE), Song Z.P. 38074 (PE), Xiong et al. 32060 (PE), Li X. 77398 (PE), Yang G.H. 65378 (PE), Yang G.H. 59414 (PE), Yu D.J. 1867 (PE), Huang et al. 1887 (PE), Zhang X.C. 0711 (PE), Wu & Cheng 115841 (PE), Anonymous 05216 (PE), Anonymous 7925 (PE), Zhang & Ren 04904 (PE), Zhang & Ren 04904 (PE), Zhang & Ren 07597 (PE),

	Zhang & Ren 04933 (PE), Zhang & Ren 05777 (PE), Zheng X.J. 0.30150 (PE), T.C.Lee 1798 (PE), Li X. 77398 (PE), Song Z.P. 38395 (PE), T.Naito et al. 225 (PE), T.Naito et al. 462 (PE), T.Naito et al. 462 (PE), Chen R. 5517 (PE), P.W.Fang 1775 (PE), P.W.Fang 6666 (PE), Fang W.P. 6666 (PE), T.H.Tu 4822 (PE), T.H.Tu 4822 (PE), Fang W.P. 6671 (PE), Fang W.P. 3966 (PE), T.T.Yu 787 (PE), T.T.Yu 787 (PE), T.T.Yu 787 (PE), Fang W.P. 3056 (PE), Fang W.P. 2710 (PE), T.T.Yu 1889 (PE), Song Z.P. 38074 (PE), Kong X.X. 1903 (PE), Wu S.G. 1945 (PE), Dai T.L. s.n. (PE), Jiang & Jin 02014 (PE), Zhu et al. 1084 (PE), H.T.Tsai 59928 (PE), H.T.Tsai 59986 (PE), H.T.Tsai 59579 (PE), K.M.Feng 5592 (PE), K.M.Feng 5592 (PE), W.Hancock 51 (PE), G.Forrest 28940 (PE), Feng G.M. 50322 (PE), Qiu B.Y. 54132 (PE), Qiu B.Y. 55004 (PE), Kunming Insitiution 51157 (PE), Kunming Insitiution 56109 (PE), C.W.Wang 69233 (PE), C.W.Wang 64110 (PE), C.W.Wang 64110 (PE), C.W.Wang 63590 (PE), C.W.Wang 70448 (PE), H.T.Tsai 57874 (PE), H.T.Tsai 59924 (PE), H.T.Tsai 59924 (PE), H.T.Tsai 57159 (PE), H.T.Tsai 57568 (PE), H.T.Tsai 57568 (PE), H.T.Tsai 59928 (PE), P.I.Mao 71 (PE), Wang & Liu 84662 (PE), T.T.Yu 8237 (PE), T.T.Yu 8237 (PE), G.Forrest 13119 (PE), G.Forrest 19841 (PE), Liu Y. 189 (PE), K.M.Feng 2380 (PE), K.M.Feng 3413 (PE), QTP Team 167 (PE), QTP Team 167 (PE), Anonymous 260a (PE), Anonymous 75-1920 (PE), Anonymous 75-1920 (PE), Anonymous 75-1920 (PE), Anonymous 180 (PE), Anonymous 180 (PE), Anonymous 75-1874 (PE), Anonymous 75-1874 (PE), Anonymous 76-423 (PE), Anonymous 76-423 (PE), Anonymous 76-423 (PE), Zhong B.Q. 6542 (PE), Zhang & Lang 928 (PE), Zhang & Lang 673 (PE), Zhang & Lang 673 (PE), Ying & Hong 650522 (PE), Ying & Hong 650522 (PE), Ying & Hong 650522 (PE), Ying & Hong 650522 (PE), Ying & Hong 650061 (PE), Ying & Hong 650061 (PE), Ying & Hong 650061 (PE), Ying & Hong 650061 (PE), Ni et al. 1322 (PE), Ni et al. 1635 (PE), Li et al. 06895 (PE), Li et al. 06895 (PE), Ni et al. 0258 (PE), Ni et al. 0258 (PE), Ni et al. 0258 (PE), Li et al. 6499 (PE), Li et al. 06499 (PE), Li et al. 02242 (PE), Li et al. 02242 (PE), Yi T.P. 79126 (PE), Yi T.P. 79076 (PE), Zhang & Wang 0333 (PE), C.W.Wang 66315 (PE), C.W.Wang 65171 (PE), C.W.Wang 65171 (PE), T.Naito et al. 854 (PE), T.Naito et al. 854 (PE), F.Ludlow et al. 6683 (PE), F.Ludlow et al. 5362 (PE), P.C.Tsoong 2564 (PE), Qin R.C. 34858 (PE), Xie Y.T. 2024 (PE), H.T.Tsai 59599 (PE), G.Forrest 28940 (PE), X.C.Zhang 4047 (PE), Qin et al. 4016 (PE), Wang Y.M. 4224 (PE), Li L.Q. 20 (PE), Mr.W.Hancock. 51 (PE), X.C.Zhang & L.Wang 4767 (PE), X.C.Zhang & L.Wang 4836 (PE), D.E.Boufford et al. 37675 (PE), Jin

	Xh,Wang LS,Wang Q et al. ST1104 (PE), FLPH Tibet Expedition 12-1931 (PE), FLPH Tibet Expedition STET0087 (PE), FLPH Tibet Expedition STET0087 (PE), FLPH Tibet Expedition 12-0936 (PE), FLPH Tibet Expedition 12-0936 (PE), FLPH Tibet Expedition 12-1931 (PE), FLPH Tibet Expedition 12-1931 (PE), FLPH Tibet Expedition 12-0779 (PE)
Pyrrosia eberhardtii (Christ) Ching	Dong S.Y. 814 (PE), Dong S.Y. 816 (PE), Zhang X.C.1604 (PE), Liang X.R. 62705 (PE), Wang C. 34416 (PE), 236-6 Team 1684 (PE), 236-6 Team 1686 (PE), Chen N.Q. 43987 (PE), Liang X.R. 62705 (PE), Liang X.R. 62705 (PE), Chen & Hou 70188 (PE), Chen & Hou 70188 (PE), Liang X.R. 65113 (PE), Liang X.R. 65113 (PE), Diaoluoshan Team 3243 (PE), S.Y.Dong 816 (PE), S.Y.Dong 814 (PE), X.C.Zhang et al. 1604 (PE), G.M.Zhang et Dong Li 076 (PE), C.Wang 34416 (PE), Zuo & Chen 44160 (PE), Tsang Wai-Tak 678 (PE), Tsang Wai-Tak 678 (PE), Tsang Wai-Tak 678 (PE), Lau S.K. 3114 (PE), Miyoshi Furuse 43987 (PE), 236-6 Team 1686 (PE)
Pyrrosia ensata Ching ex K.H. Shing	QTP Coll. Team 9235 (PE), Li B.S. 04299 (PE), Feng G.M. 5114 (PE)
Pyrrosia fengiana Ching	Feng K.M. 7977 (PE), QTP Coll. Team 5580 (PE), QTP Coll. Team 7394 (PE), Wu C. ch0904009 (PE), Zhang & Dong 1429 (PE), K.M.Feng7977 (PE), Jiang S.8736 (PE), QTP Team5646 (PE), Anonymous260a (PE), T.Naito et al.118 (PE)
Pyrrosia flocculosa (D. Don) Ching	Zhang X.C. 2927 (PE), Li & Li 14160 (PE), Zhao J.C. 5948 (PE), Zhang X.C. & Dong S.Y. 1410 (PE), A.H.G.Alston 15157 (PE), S.P.Khullar et al. 44 (PE), Hideo Tabata et al. 1480 (PE), Anonymous s.n. (PE), R.E.Holttum 25358 (PE), R.E.Holttum 31343 (PE), Anonymous 96 (PE), Anonymous 0176 (PE), Guangxi Team 3275 (PE)
Pyrrosia gralla (Giesenh.) Ching	Wei X.P. wys11 (IMD), Zhang X.C. 5667 (PE), Zhang X.C. 5913 (PE), Anonymous 50552 (PE), Liu K.R. 0359 (PE), Zhou H.C. 2683 (PE), Zhou H.C. 2683 (PE), Zhou H.C. 265 (PE), Zhou H.C. 2479 (PE), Chang 00740 (PE), B.Bartholomew et al. 1714 (PE), Wu Z.L. 33277 (PE), Jiang & Jin 01503 (PE), Jiang & Jin 00645 (PE), Anonymous 9485 (PE), Anonymous 9570 (PE), Anonymous 0071 (PE), Li & Zhu 75201 (PE), Li & Zhu 76338 (PE), Li 76637 (PE), Li & Zhu 76916 (PE), Li X. 70765 (PE), Li & Zhu 75355 (PE), Li X. 77307 (PE), Li X. 70115 (PE), Li & Zhu 76511 (PE), Li X. 71007 (PE), Li X. 71221 (PE), Zhang X.C. 2134 (PE), QTP Team 14090 (PE), QTP Team 14090 (PE), QTP Team 13460 (PE), QTP Team 13460 (PE), Lang et al. 2125 (PE), Lang et al. 2125 (PE), QTP Team 12525 (PE), Jiang S. 9717 (PE), Jiang S. 9717 (PE), Jiang & Jin 02024 (PE), Zhang D.H.

	1372 (PE), Zhou H.C. 575 (PE), He Y.Q. 1745 (PE), Zhou H.C. 1051 (PE), Ying J.S. 3410 (PE), Huang et al. 679 (PE), Chen Q.H. 4532 (PE), Anonymous 05256 (PE), Qiu & Liu 4506 (PE), Zhang & Ren 06720 (PE), Li X. 73818 (PE), Li X. 76694 (PE), Anonymous 05696 (PE), Jiang S. 8839 (PE), Yi T.P. 74438 (PE), Yi T.P. 74438 (PE), Kong X.X. 3549 (PE), Li X. 76561 (PE), Li & Zhu 75853 (PE), Li & Zhou 73730 (PE), Li & Zhou 73805 (PE), Li X. 72470 (PE), Li X. 71219 (PE), Li X. 76241 (PE), Li X. 70118 (PE), Li X. 70039 (PE), Li X. 71067 (PE), Li X. 73612 (PE), Li & Zhou 73888 (PE), Zhou & Su 107644 (PE), Zhou & Su 110932 (PE), Zhou & Su 109154 (PE), Anonymous 14138 (PE), Anonymous 14138 (PE), Dai T.L. 106835 (PE), Zhang & Zhou 0019 (PE), Anonymous 9410 (PE), He & Zhou 14219 (PE), Song Z.P. 39304 (PE), Jiang X.L. 35038 (PE), Li X. 46672 (PE), Zhang S.F. 854 (PE), T.Naito et al. 406 (PE), Anonymous 1710 (PE), Anonymous 22785 (PE), Anonymous 76561 (PE), Anonymous 76511 (PE), Anonymous 73888 (PE), Anonymous 76338 (PE), Anonymous 76791 (PE), Anonymous 75691 (PE), Yu P.H 1389 (PE), Suzuki-Tokio s.n. (PE), Anonymous s.n. (PE), Qin R.C. s.n. (PE), Liu S.E. 019210 (PE), Liu S.E. 15733 (PE), Jiang Y. 16529 (PE), Wang K. 92005 (PE), Wang K. 92007 (PE), Qiu B.Y. 54440 (PE), Qiu B.Y. 54440 (PE), Qiu B.Y. 54535 (PE), Wang & Zhu C811 (PE), Wang & Zhu C811 (PE), Wang & Zhu C811 (PE), Wang & Li C86961 (PE), Wang & Li C86961 (PE), Liu H.M. YN294 (PE), Liu H.M. YN287 (PE), Wang Z.R. 324 (PE), Lan S.B. 227 (PE), QTP Team 7371 (PE), QTP Team 6305 (PE), Feng G.M. 04535 (PE), Feng G.M. 04535 (PE), Anonymous 19024 (PE), Li Y.H. 169 (PE), Zhu & Liu 1774 (PE), Zhu W.M. 1621 (PE), Zhu et al. 1648 (PE), Zhu & Wu 01743 (PE), Kunming Insitiution 39 (PE), Kunming Insitiution 51863 (PE), Qin R.C. s.n. (PE), Qin R.C. s.n. (PE), K.M.Feng 3448 (PE), K.M.Feng 2490 (PE), Maire 2490 (PE), Liu S.E. 13640 (PE), G.Forrest 20374 (PE), QTP Team 7732 (PE), Xu C.D. 30000 (PE), X.C.Zhang 4005 (PE), X.C.Zhang 4005 (PE), Liu H.M. GX254 (PE), Chen et al. 12564 (PE), Wang Z.R. 376 (PE), Liu Z.Y. 1380 (PE), D.E.Bofford et al. 34869 (PE), Xi & Qin 07907 (PE), Qi X.P. Q046 (PE)
Pyrrosia hastata (Thunb. ex Houtt.) Ching	Togasi M. 1362 (PE), Zhang X.C. 5553 (PE), Anonymous s.n. (PE), M.Togasi 1362 (PE), K.H.Shing 63 (PE), W.P.Brooks 611 (PE), Taquet 3829 (PE), Anonymous 506 (PE), K.Satake s.n. (PE), J.Uchiyama s.n. (PE), U.Faurie 1563 (PE), Ching R.C. 1780 (PE)
Pyrrosia heteractis (Mett. ex Kuhn) Ching	Zhang et al. 6264 (PE), Zhang X.C. 5052 (PE), Wang W.C. 74393 (PE), Wang Z.R. 761 (PE), Liu S.E. 22434 (PE), Liu S.E. 018619 (PE), Wang Z.R. 761 (PE), Wang Z.R. 543 (PE), Zhang Z.Y. 64-41 (PE), Han et al. 81-547

	(PE), C.W.Wang 74393 (PE), C.W.Wang 73818 (PE), C.W.Wang 77239 (PE), C.W.Wang 67112 (PE), C.W.Wang 67467 (PE), C.W.Wang 78407 (PE), H.T.Tsai 55435 (PE), H.T.Tsai 62712 (PE), H.T.Tsai 54297 (PE), H.T.Tsai 54297 (PE), H.T.Tsai 54297 (PE), K.M.Feng 13730 (PE), T.T.Yu 20188 (PE), T.T.Yu 20188 (PE), T.T.Yu 20188 (PE), T.T.Yu 20545 (PE), T.T.Yu 20545 (PE), T.T.Yu 20545 (PE), T.T.Yu 20545 (PE), Ching R.C. s.n. (PE), Ching R.C. 50758 (PE), Ching R.C. 25433 (PE), Ching R.C. 25433 (PE), Ching R.C. 25433 (PE), K.M.Feng 7029 (PE), K.M.Feng 7029 (PE), K.M.Feng 7029 (PE), K.M.Feng 7314 (PE), K.M.Feng 7314 (PE), K.M.Feng 8015 (PE), K.M.Feng 8015 (PE), K.M.Feng 4465 (PE), K.M.Feng 4465 (PE), Wenshan Team 60-0138 (PE), Jiang et al. 8696 (PE), Jiang et al. 8696 (PE), Dong Q.Z. 705 (PE), Dong Q.Z. s.n. (PE), China-Russia Team 4439 (PE), Wang Z.R. 761 (PE), Wu S.G. 4157-62 (PE), Forrest 25094 (PE), G.Forrest 26697 (PE), G.Forrest 20201 (PE), G.Forrest 26697 (PE), Ni et al. 0938 (PE), Ni et al. 0377 (PE), Li et al. 03721 (PE), Li et al. 03721 (PE), Li et al. 03721 (PE), Li & Cheng 01333 (PE), Li & Cheng 01333 (PE), Li & Cheng 01333 (PE), Li & Cheng 01333 (PE), Li & Cheng 02133 (PE), Li & Cheng 02133 (PE), Li & Cheng 02133 (PE), Anonymous 309 (PE), Anonymous 574 (PE), Anonymous 309 (PE), Anonymous 250 (PE), Anonymous 250 (PE), Zhang J.W. 0864 (PE), QTP Team 932 (PE), QTP Team 932 (PE), Anonymous 574 (PE), Yi T.P. 79164 (PE), Jia S.X. 1977 (PE), Anonymous s.n. (PE), F.Ludlow et al. 7040 (PE), Anonymous 66951 (PE), Ching R.C. 50758 (PE), G.Forrest 25094 (PE), X.C.Zhang 5052 (PE), Zhang X.C. 4046 (PE)
Pyrrosia kinabaluensis Hovenkamp	Wei et al. 457 (PE)
Pyrrosia laevis (J. Sm. ex Bedd.) Ching	Shing et al. 6769 (PE), China-Russia Team 7936 (PE), China-Russia Team 7935 (PE), H.F.Sun s.n. (PE), Anonymous s.n. (PE), Anonymous s.n. (PE), Anonymous s.n. (PE), Yan Y.H. 1785 (PE), Shui et al. 80231 (PE), Shui et al. 80096 (PE)
Pyrrosia lanceolata (L.) Farw.	Li Z.Y. lzy02 (IMD), Li Z.Y.1924 (PE), Zhang X.C. 1735 (PE), Zhang X.C. 1422 (PE), QTP Coll. Team4194 (PE), Erl.Christophersen 446 (PE), X.C.Zhang et al. 1422 (PE), X.C.Zhang 1735 (PE), Wang & Liu 86200 (PE), C.W.Wang 73169 (PE), QTP Team 4194 (PE), QTP Team 4194 (PE), QTP Team 1910 (PE), QTP Team 1910 (PE), QTP Team 1785 (PE), QTP Team 1785 (PE), QTP Team 5039 (PE), QTP Team 5039 (PE), Cheng & Li 04613 (PE), Cheng & Li 04613 (PE), Cheng & Li 04008 (PE), Cheng & Li 04008 (PE), Cheng & Li 04008 (PE), Cheng & Li 04008 (PE), Cheng & Li 04008 (PE), Cheng & Li 04008 (PE), Cheng & Li 03901 (PE), Cheng & Li

	03901 (PE), Chen W.L. 10906 (PE), Chen W.L. 10906 (PE), Chen W.L. 10906 (PE), Anonymous s.n. (PE), H.F.Sun 663 (PE), Anonymous 1837 (PE), Anonymous 16588 (PE), H.F.Sun s.n. (PE), Anonymous s.n. (PE), Anonymous s.n. (PE), Robert L.Fleming 2428 (PE), Robert L.Fleming 2428 (PE), B.M.Eller 88-005 (PE), A.H.G.Alston 13578 (PE), Lin M.Y. s.n. (PE), Ajaula Nujoy s.n. (PE), C.O.Levine s.n. (PE), South Tibet Exp. Team (STET) STET1924 (PE)
Pyrrosia linearifolia (Hook.) Ching	Shing K.H. 78 (PE), Furuse M. 42814(PE), Sato J. 10161 (PE). Zhang X.C. 5551 (PE), Wang et al. 3946 (PE), Wang et al. 4071 (PE), Tianye et al. 154 (PE), Tianye et al. 154 (PE), J.sato 10161 (PE), J.sato 10161 (PE), Herbarium 82725 (PE), Anonymous s.n. (PE), N.Formosa 194 (PE), W.Hancock 96 (PE), Taquet 3854 (PE), J.Uchiyama s.n. (PE), Anonymous s.n. (PE), K.H.Shing 89 (PE), K.H.Shing 78 (PE), M.Togasi 1366 (PE), M.Togasi 1366 (PE), M.Togasi 1366 (PE), M.Togasi 1366 (PE), M.Togasi 1366 (PE), M.Togasi 1366 (PE), M.Togasi 1366 (PE), Palph G.Mills M.D. s.n. (PE), Palph G.Mills M.D. s.n. (PE), Palph G.Mills M.D. s.n. (PE), Y.Miyoshi s.n. (PE), M.Hutoh 12094 (PE), M.Tagawa 7322 (PE), M.Tagawa et al. 1522 (PE), E.Zogg et al. 11295 (PE), N.Satomi s.n. (PE), Miyoshi Furuse 36761 (PE), Miyoshi Furuse 23384 (PE), Miyoshi Furuse 11025 (PE), Miyoshi Furuse 11644 (PE), Miyoshi Furuse 34015 (PE), Miyoshi Furuse 32164 (PE), Miyoshi Furuse 32141 (PE), Miyoshi Furuse 34566 (PE), Faurie 3669 (PE), Miyoshi Furuse 52294 (PE), Miyoshi Furuse 55148 (PE), Miyoshi Furuse s.n. (PE), Miyoshi Furuse 56688 (PE), Miyoshi Furuse 55117 (PE), Miyoshi Furuse 42814 (PE), Miyoshi Furuse 42814 (PE), Miyoshi Furuse 42814 (PE), Miyoshi Furuse 43061 (PE), Miyoshi Furuse 43965 (PE), Miyoshi Furuse 43965 (PE), Miyoshi Furuse 55117 (PE), Miyoshi Furuse 43965 (PE)
Pyrrosia lingua (Thunb.) Farw.	Wei X.P. wys120206 (IMD), Wei et al. wxp009 (PE), Wang F.G. 1341 (PE), Zhu et al. 1268 (PE), Li D. 23 (PE), Zhang & Liu 6048 (PE), Faurie 02479 (PE), Miyoshi Furuse 2497 (PE), Miyoshi Furuse 2293 (PE), Miyoshi Furuse 7082 (PE), Miyoshi Furuse 5176 (PE), Miyoshi Furuse 2382 (PE), Miyoshi Furuse 4865 (PE), Miyoshi Furuse 2096 (PE), Miyoshi Furuse 2494 (PE), Miyoshi Furuse 2494 (PE), Miyoshi Furuse Q136-29 (PE), Miyoshi Furuse Q110-21 (PE), Miyoshi Furuse 84 (PE), Miyoshi Furuse F1013 (PE), X.C.Zhang et al. 1578 (PE), X.C.Zhang et al. 1577 (PE), X.C.Zhang et al. 1578 (PE), X.C.Zhang et al. 1562 (PE), G.M.Zhang et Dong Li 149 (PE), G.M.Zhang et Dong Li 138 (PE), H.Ohashi et al. 12790 (PE), Wuzhishan Fern Survey 453 (PE), H.Ohashi et al. 574 (PE), X.C.Zhang et al. 1670 (PE), Liu Z.Y. 19220 (PE), Liu Z.Y. 13823 (PE), Liu Z.Y. 15047 (PE), Liu

	Z.Y. 16682 (PE), Beijing Team 4280 (PE), Beijing Team 001609 (PE), Beijing Team 000953 (PE), C.O.Levine s.n. (PE), C.O.Levine s.n. (PE), Yan & Zhou 3362 (PE), Yan & Zhou 13402 (PE), Wu & Qi 1305 (PE), Wu & Qi 1227 (PE), Wu & Qi 1226 (PE), Qing & Xu 648 (PE), Qing & Xu 611 (PE), Qing & Xu 620 (PE), Qing & Xu 208 (PE), Yan & Liu 0602088 (PE), 236-6 Team 1871 (PE), L.Q.Li et al. 0641 (PE), L.Q.Li et al. 0635 (PE), Wu et al. 870 (PE), Wu et al. 638 (PE), Wu et al. 985 (PE), Wu et al. 569 (PE), Wu et al. 180 (PE), Wu et al. 176 (PE), Wu et al. 255 (PE), Wu et al. 333 (PE), Wu et al. 237 (PE), Zhou & Zhao 143 (PE), Zhou & Zhao 266 (PE), Zhou & Zhao 254 (PE), Zhou & Zhao 254 (PE), D.E.Boufford et al. A150072 (PE), Guizhou Team 15 (PE), Guizhou Team s.n. (PE), Xinan Team 0015 (PE), Xinan Team 0015 (PE), Zhang & Li 2945 (PE), Zhang & Li 1134 (PE), Zhang & Li 3178 (PE), Zhang & Li 3178 (PE), Zhang & Li 2945 (PE), Liu Z.Y. 181488 (PE), Liu Z.Y. 181572 (PE), Longxishan Team 0999 (PE), Longxishan Team 822 (PE), Longxishan Team 839 (PE), Longxishan Team 0301 (PE), Longxishan Team 0383 (PE), Longxishan Team 0950 (PE), Longxishan Team 0950 (PE), Longxishan Team 0950 (PE), Longxishan Team 0294 (PE), Bashan Team 0216 (PE), Xing G.X. 6296 (PE), Qi X.P. Q067 (PE), Shui et al. 80901 (PE), Guangxi Team 0525 (PE), Gannan Team 1019 (PE), Guangxi Team 4265 (PE), Qiannan Team 240 (PE), Qiannan Team 1015 (PE), FLPH Tibet Expedition 0901045 (PE), Qin & Qi 1451 (PE), Tetsuji Yamanaka s.n. (PE), Sanxia Team 1989 (PE)
Pyrrosia longifolia (Burm. f.) C.V. Morton	Zhang et al. 082 (PE), Wei et al. 2446 (PE), Zhang X.C. 6540 (PE), Fung H. 20204 (PE), Wang C. 33397 (PE), Ching-Russia Yuannan Team 9392 (PE), Ching-Russia Yuannan Team 9392 (PE), Ching-Russia Yuannan Team 9392 (PE), Ching-Russia Yuannan Team 9392 (PE), C.Wang 33361 (PE), H.Y.Liang 64277 (PE), C.Wang 35885 (PE), H.Y.Liang 63426 (PE), H.Y.Liang 61999 (PE), F.C.How 70505 (PE), H.Y.Liang 64297 (PE), S.P.Ko 52241 (PE), Zuo & Chen 43439 (PE), Fung H. 20204 (PE), Fung H. 20204 (PE), Fung H. 20204 (PE), Fung H. 20204 (PE), S.Y.Dong 934 (PE), S.Y.Dong et al. 550 (PE), Huang Q. 1447 (PE), Huang Q. 33597 (PE), Liang X.R. 64277 (PE), Deng L. 2503 (PE), H.F.Sun 284 (PE), H.F.Sun 354 (PE), H.F.Sun 43 (PE), H.F.Sun s.n. (PE), H.F.Sun 113 (PE), H.F.Sun 354 (PE), Anonymous 501 (PE), Anonymous 6833 (PE), Anonymous s.n. (PE), H.F.Sun s.n. (PE), H.F.Sun s.n. (PE), A.H.G.Alston 16087 (PE), A.H.G.Alston 12869 (PE), C.F.van Beusekom et al. 679 (PE), Anonymous 9093 (PE), Mr.W.Hancock. 96 (PE)

Pyrrosia mannii (Giesenh.) Ching	Zhao J.C. zjc s.n. (PE), Zhang X.C. 5311 (PE), Qi X.P. q088 (PE), Fung H. 3248 (PE), Fung H. 4427 (PE), S.Y.Dong 4427 (PE), S.Y.Dong et al. 2223 (PE), Robert L.Fleming 2185 (PE), Hideo Tabata et al. 0989 (PE), Hideo Tabata et al. 1572 (PE), F.M.Jarrett et al. 1003 (PE), Griffith s.n. (PE), Mr.W.Hancock. 483 (PE), Xu Y.C. 483 (PE), Xu Y.C. 483 (PE), Xu Y.C. 483 (PE), Xu S.G. 9765 (PE), Ching-Japan Team T359 (PE), Li & Li 14076 (PE), Li & Li 14076 (PE), Li & Li 14076 (PE), Zhang & Lang 3246 (PE), Zhang & Lang 3246 (PE), Zhang & Lang 3244 (PE), Zhang & Lang 3244 (PE), Zhang & Lang 3248 (PE), Qi X.P. Q088 (PE),
Pyrrosia martini (Christ) Ching	Yang & Oyang 008 (PE), Yan et al. 20071201 (PE), Beijing Team 001802 (PE), Wulingshan Team 2000 (PE), Guan & Wang 2542 (PE), Jian Z.P. 32472 (PE), Jian et al. 30234 (PE), Jian et al. 32492 (PE), Zhang et al. 401990 (PE), Jian et al. 31204 (PE), Jian et al. 32492 (PE), Zhang et al. 400407 (PE), Yan et al. 5122 (PE), Yan et al. 5122 (PE), Liu B.R. 850390 (PE), Liu B.R. 850390 (PE), Liu B.R. 850390 (PE), Liu B.R. 850390 (PE), Liu B.R. 900115 (PE), Liu B.R. 900115 (PE), Liu B.R. 900115 (PE), Liu B.R. 850449 (PE), Liu B.R. 850449 (PE), Liu B.R. 850449 (PE), Liu B.R. 850449 (PE), Liu B.R. 871143 (PE), Liu B.R. 871143 (PE), Liu B.R. 871143 (PE), Liu B.R. 871143 (PE), Liu B.R. 871143 (PE), Anonymous 19218 (PE)
Pyrrosia nuda (Giesenh.) Ching	Xu C.D. 29958 (PE), Li Z.Y. lzy04 (IMD), Shui Y.M. 20071122 (PE), Li Z.Y. & Wei R. wr0254 (PE), C.W.Wang 73862 (PE), C.W.Wang 73556 (PE), C.W.Wang 76951 (PE), C.W.Wang 73182 (PE), C.W.Wang 79605 (PE), C.W.Wang 79881 (PE), Anonymous 12884B (PE), Anonymous s.n. (PE), Anonymous s.n. (PE), B.Bartholomew et al. 1595 (PE), Anonymous 337 (PE), Anonymous 337 (PE), Jiang X.L. 50794 (PE), Jiang X.L. 50794 (PE), China-Russia Yunnan Team 5547 (PE), China-Russia Yunnan Team 9396 (PE), China-Russia Yunnan Team 7178 (PE), China-Russia Yunnan Team 2613 (PE), China-Russia Yunnan Team 201 (PE), China-Russia Yunnan Team 9396 (PE), China-Russia Yunnan Team 1640 (PE), China-Russia Yunnan Team 5547 (PE), China-Russia Yunnan Team 5547 (PE), China-Russia Yunnan Team 7178 (PE), China-Russia Yunnan Team 5547 (PE), Qin R.C. 50805 (PE), Qin R.C. 50805 (PE), Dong Q.Z. 202 (PE), Xing et al. 06855 (PE), Xing et al. 06932 (PE), Tsi Zhanhuo 91-252 (PE), China-Russia Yunnan Team 9396 (PE), Qin R.C. 50794 (PE), Qin R.C. 50795 (PE), Qin R.C. 50795 (PE), Qin R.C. 50805 (PE), Qin R.C. 50805 (PE), Qin R.C. 50811 (PE), Qin R.C. 50811 (PE), Anonymous 2501 (PE), Cai K.H. 337 (PE), Cai K.H. 337 (PE), Xu Y.C. 370 (PE), Xu Y.C. 370 (PE), Xu Y.C. 370 (PE), Xu Y.C. 291 (PE), Xu Y.C. 291 (PE), Xu Y.C. 291 (PE), Xu Y.C. 178 (PE), Xu Y.C. 178

	(PE), Xu Y.C. 178 (PE), Xu Y.C. 178 (PE), Feng G.M. 20864 (PE), Feng G.M. 20864 (PE), Mao P.Y. 20189 (PE), Anonymous s.n. (PE), Anonymous s.n. (PE), China-Russia Yunnan Team 201 (PE), China-Russia Yunnan Team 7618 (PE), Kunming Institution 353/3 (PE), Liu Z.E. 018143 (PE), Pei S.J. 59-9477 (PE), Y.Tsiang 12908 (PE), Y.Tsiang 12908 (PE), M.K.Li 1496 (PE), C.W.Wang 76173 (PE), C.W.Wang 74740 (PE), Zhang & Chen 4085 (PE), Shui et al. 80405 (PE), Shui et al. 80045 (PE), C.W.Wang 74955 (PE), Zhang & Li 5647 (PE), Zhang X.C. 6728 (PE)
Pyrrosia nudicaulis Ching	Zhang et al. 6261 (PE), Zhang et al. 6265 (PE), Wang C.W. 65188 (PE), QTP Coll. Team 6315 (PE), Anonymous 71912 (PE), Anonymous 10455 (PE), T.T.Yu 10455 (PE), T.T.Yu 10455 (PE), H.T.Tsai 57140 (PE), H.T.Tsai 57140 (PE), H.T.Tsai 63012 (PE), H.T.Tsai 63012 (PE), C.W.Wang 69248 (PE), C.W.Wang 69337 (PE), C.W.Wang 69337 (PE), C.W.Wang 69306 (PE), C.W.Wang 69306 (PE), C.W.Wang 69248 (PE), C.W.Wang 69248 (PE), C.W.Wang 68310 (PE), C.W.Wang 68310 (PE), C.W.Wang 64145 (PE), C.W.Wang 64145 (PE), C.W.Wang 64132 (PE), C.W.Wang 64132 (PE), C.W.Wang 70435 (PE), C.W.Wang 70435 (PE), C.W.Wang 67889 (PE), T.T.Yu 23009 (PE), T.T.Yu 23009 (PE), T.T.Yu 23009 (PE), Qu G.L. 2315 (PE), Qu G.L. 2315 (PE), Xie Z.J. 40210 (PE), Qin R.C. 23303 (PE), Qin R.C. 23303 (PE), Qin R.C. 25236 (PE), K.M.Feng 5669 (PE), K.M.Feng 5669 (PE), K.M.Feng 3639 (PE), K.M.Feng 7093 (PE), Qin R.C. 24771 (PE), Qin R.C. 24771 (PE), Jiang et al. 8655 (PE), Jiang et al. 8655 (PE), Kunming Institution 23868 (PE), Kunming Institution 23626 (PE), Kunming Institution 23754 (PE), Jiang et al. 9113 (PE), C.W.Wang 65188 (PE), C.W.Wang 65188 (PE)
Pyrrosia nummulariifolia (Sw.) Ching	Wei et al. slws-08 (PE), Shui et al. 80085 (PE), Li Z.Y. lzy03 (IMD), China-Russia Yuannan Team 799 (PE), China-Russia Yuannan Team 977 (PE), China-Russia Yuannan Team 1929 (PE), Xing et al. 6869 (PE), Xing et al. 6869 (PE), Qin R.C. s.n. (PE), M.K.Li 1498 (PE), M.K.Li 1498 (PE), H.F.Sun s.n. (PE), Anonymous s.n. (PE), H.F.Sun s.n. (PE), H.F.Sun 402 (PE), Anonymous s.n. (PE), Anonymous s.n. (PE), Anonymous s.n. (PE), Anonymous 8843 (PE), R.S.Williams 2260 (PE), B.Bartholomew et al. 3793 (PE), Shui et al. 80085 (PE), Shui et al. 80710 (PE)
Pyrrosia oblonga Ching	Ching R.C. 8117 (PE), Jiang R.H. 061 (PE)
Pyrrosia penangiana (Hook.) Holtt.	Wei et al. 3014 (PE), Holttum R.E. 25239 (PE), A.H.G.Alston 13741 (PE), W.Hancock 1892 (PE)

Pyrrosia petiolosa (Christ) Ching	He et al. 6735 (PE), Wei X.P. wxp217 (PE), Wu C. ch081007 (PE), Wei R. wr035 (PE), Wan T. FSB-781A (PE), Liu Z.Y. 13842 (PE), C.Y.Wu 64 (PE), Plants Investigation Team 0894 (PE), Plants Investigation Team 0725 (PE), Wang et al. 438 (PE), Wang et al. 144 (PE), Wang et al. 144 (PE), Li et al. 3337 (PE), Zhang et al. 1889 (PE), Ao Z.W. 012 (PE), Zhang Y.L. 19 (PE), Zhou et al. 6133 (PE), Zhou et al. 6348 (PE), Zhou et al. 4648 (PE), Zhou et al. 2159 (PE), Shandong Investigation Team 1580 (PE), Zhang & Liu 0097 (PE), Anonymous s.n. (PE), Anonymous s.n. (PE), Anonymous 0274 (PE), Resource Investigation Team D0100 (PE), Resource Investigation Team D0100 (PE), Resource Investigation Team D0100 (PE), Resource Investigation Team D0100 (PE), Resource Investigation Team D1184 (PE), Resource Investigation Team D1184 (PE), An Z.J. T0430 (PE), An Z.J. T0430 (PE), An Z.J. T0430 (PE), An Z.J. T0430 (PE), Resource Investigation Team L0416 (PE), Resource Investigation Team L0416 (PE), Resource Investigation Team L0416 (PE), Resource Investigation Team D550 (PE), Resource Investigation Team D550 (PE), Resource Investigation Team D550 (PE), C.Y.Wu 64 (PE), Zhang X.Q. 20300 (PE), Chen & Min 037 (PE), Chen & Min 037 (PE), Chen & Min 037 (PE), Guan & Dai 529 (PE), Guan & Dai 551 (PE), Guan & Dai 702 (PE), Guan & Dai 909 (PE), Guan & Dai 909 (PE), Guan & Dai 2070 (PE), Guan & Dai 175 (PE), Anonymous 34169 (PE), Anonymous 34169 (PE), Anonymous 34169 (PE), Anonymous 0097 (PE), Anonymous 0097 (PE), Henan Forestry Bureau 771 (PE), Henan Forestry Bureau 771 (PE), T.H.Shih 51 (PE), He Z.Z. 8631 (PE), He Z.Z. 6254 (PE), K.M.Liou 4872 (PE), K.M.Liou 4872 (PE), K.M.Liou 4872 (PE), K.M.Liou 4373 (PE), D.E.Boufford et al. 26496 (PE), T.N.Liou & C.Wang 34 (PE), T.P.Wang 2188 (PE), T.P.Wang 2188 (PE), K.S.Hao 4432 (PE), Anonymous s.n. (PE), Wang et al. C701 (PE), Wang et al. C701 (PE), Wang et al. C701 (PE), P.Licent S.J. 8433 (PE), P.Licent S.J. 8812 (PE), F.H.Chen 160 (PE), F.H.Chen 75 (PE), H.W.Kung 2037 (PE), F.H.Chen 160 (PE), F.H.Chen 210 (PE), Lin C.Q. 486 (PE), Li et al. 2589 (PE), Li et al. 101 (PE), Li et al. 101 (PE), Tianye et al. 410 (PE), Zhou et al. 2874 (PE), Li et al. 304 (PE), Li et al. 471 (PE), H.W.Kung 596 (PE), H.W.Kung 871 (PE), Bao F.H. 89-38 (PE), P.Licent S.J. 8283 (PE), P.Licent S.J. 3590 (PE), P.Licent S.J. 3615 (PE), P.Licent S.J. 9836 (PE), Anonymous 34 (PE), Nankai University 350 (PE), Nankai University 350 (PE), Nankai University 350 (PE), P.Licent S.J. 10092 (PE), Wang F.Z. 00227 (PE), Liu & Liu 13710 (PE), Liu & Liu 13710 (PE), Liu & Liu 13710 (PE), Liu & Liu 13112 (PE), Liu & Liu 13112 (PE), Wang et al. 2753 (PE), Wang et al. 2753 (PE), Wang et al. 2753 (PE), Wang et al. 2753 (PE), Liu &

	Zhao 530 (PE), Liu & Zhao 530 (PE), Wang et al. 2596 (PE), Liu & Liu 13614 (PE), Liu & Liu 13614 (PE), Liu & Zhao 00256 (PE), Liu & Zhao 00301 (PE), Liu & Zhao 00301 (PE), Liu & Zhao 00256 (PE), Anonymous s.n. (PE), Guan K.J. 5721 (PE), C.W.Wang 62592 (PE), C.W.Wang 62592 (PE), C.W.Wang 62593 (PE), C.W.Wang 62593 (PE), C.W.Wang 60784 (PE), C.Y.Yuang et al. 36426 (PE), T.N.Liou 904 (PE), K.M.Liou 646 (PE), Anonymous 1293 (PE), Anonymous 3431 (PE), Chen & Ni 34 (PE), Chen & Ni 34 (PE), K.M.Liou 1107 (PE), K.M.Liou 1107 (PE), K.M.Liou 3564 (PE), K.M.Liou 3564 (PE), K.M.Liou 3844 (PE), J.sato 5997 (PE), Nankai University 0341 (PE), Liu Z.E. s.n. (PE), Y.C.Wang 575 (PE), Y.C.Wang 575 (PE), T.N.Liou & K.M.Liou 1437 (PE), Anonymous s.n. (PE), China-Germany Team 566 (PE), Li F.Z. 0146 (PE), Li J.X. s.n. (PE), Resource Investigation Team D550 (PE), K.S.Hao 4432 (PE), T.P.Wang 877 (PE), T.P.Wang 877 (PE), T.P.Wang 9141 (PE), T.P.Wang 9141 (PE), Zhou H.C. 3899 (PE), T.P.Wang 11192 (PE), B.Bartholomew et al. 438 (PE), B.Bartholomew et al. 438 (PE), Guan & Wang 2753 (PE), Guan & Wang 2753 (PE), Guan & Wang 1366 (PE), Guan & Wang 1366 (PE), Guan & Wang 2205 (PE), P.C.Tsoong 12195 (PE), Anonymous 875 (PE), Anonymous 1291 (PE), Anonymous 973 (PE), Anonymous D202 (PE), Anonymous 1291 (PE), Anonymous s.n. (PE), Anonymous s.n. (PE), Palph G.Mills M.D. s.n. (PE), Palph G.Mills M.D. s.n. (PE), Palph G.Mills M.D. s.n. (PE), Palph G.Mills M.D. s.n. (PE), O.Zhurba s.n. (PE), T.N.Liou & P.C.Tsoong 3839 (PE), T.N.Liou & P.C.Tsoong 3839 (PE), Lai C.G. 217 (PE), Wang et al. 574 (PE), Xie Y.T. 2017 (PE), Yellow River Team 192 (PE), Zhang Z.Y. 15883 (PE), Qiao Y.L. 1258 (PE), Zhang Z.W. 1752 (PE), Wang Z.B. 15784 (PE), Peng & Zhang 85 (PE), P.Licent S.J. 2492 (PE), Fan F.D. 16 (PE), Anonymous 896 (PE), He X.Y. 932 (PE), Zhang Z.Y. 15154 (PE), Zhang Z.Y. 14941 (PE), Zhang Z.Y. 14787 (PE), Liu J.M. 10033 (PE), Anonymous 2543 (PE), Anonymous 1418 (PE), Anonymous 189 (PE), H.Migo s.n. (PE), Anonymous 0891 (PE), Guan K.J. 178 (PE), Guan K.J. 178 (PE), Ding & Wang 0491 (PE), Anonymous 21255 (PE), Ding & Wang 1085 (PE), Yuan et al. 2559 (PE), Lan & Jin 75077 (PE), Anonymous 228 (PE), Wang et al. 1912 (PE), W.C.Cheng 223 (PE), C.R.Chu 2924 (PE), H.P.Chang 79 (PE), Chin & Shun 33 (PE), C.Y.Chiao 2695 (PE), Qiu P.X. 85 (PE), Guan K.J. 75417 (PE), Guan K.J. 75417 (PE), Qiu P.X. 5227 (PE), Lu J.Q. 556 (PE), Anonymous 30659 (PE), Anonymous 29060 (PE), Anonymous 22597 (PE), He X.Y. 27522 (PE), Anonymous 29770 (PE), Wang J.M. 0177 (PE), Wang J.M. 0551 (PE), Wang J.M. 0177 (PE), Tan C.M. 92410 (PE), Y.Tsiang 10105 (PE), Anonymous 5726 (PE), Xing D. 6265 (PE), Xing

	& Xia 5631 (PE), Anonymous 542 (PE), Li Z.T. 2951 (PE), Liu Q.X. 20 (PE), Shennongjia Team 20215 (PE), Shennongjia Team 20215 (PE), Shennongjia Team 20099 (PE), Shennongjia Team 20099 (PE), Zhou D. 76100 (PE), Zheng H.H. 40 (PE), Qian M.Z. 1582 (PE), Zhou H.C. 264 (PE), Zhou H.C. 264 (PE), Zhou H.C. 2438 (PE), Lang K.Y. 03063 (PE), QTP Team 12013 (PE), QTP Team 12013 (PE), Kong X.X. 5580 (PE), Kong X.X. 5580 (PE), Yi T.P. 75101 (PE), Liu Z.Y. 15310 (PE), Kong X.X. 3520 (PE), Kong X.X. 3551 (PE), Zhang & Ren 4558 (PE), Yang G.H. 59563 (PE), Zhao et al. 1000356 (PE), Guan Z.T. 8928 (PE), Yu D.J. 3542 (PE), Yu D.J. 3223 (PE), Zhou & Xu 648 (PE), Anonymous 1910 (PE), Xie & Kong 40209 (PE), Xie & Kong 41721 (PE), Xie & Kong 40756 (PE), Zhang S.F. 383 (PE), Wan W.P. 5667 (PE), Li X. 77369 (PE), Li G.F. 64979 (PE), Li G.F. 60186 (PE), Anonymous 60228 (PE), Lang et al. 488 (PE), Lang et al. 488 (PE), Lang et al. 571 (PE), Lang et al. 571 (PE), Liu Z.Y. 15959 (PE), Guan Z.T. 8928 (PE), Yu D.J. 776 (PE), Liu et al. 10086 (PE), Zhu et al. 069 (PE), Kong X.X. 3534 (PE), Kong X.X. 6284 (PE), Anonymous 8470 (PE), Anonymous 8762 (PE), Anonymous 13376 (PE), Dai T.L. 100184 (PE), Yang G.H. 65606 (PE), Fang M.Y. 24969 (PE), Dai T.L. 107172 (PE), Dai T.L. 103743 (PE), Dai T.L. 104888 (PE), Fang M.Y. 24997 (PE), Dai T.L. 101634 (PE), Dai T.L. 103427 (PE), Fang M.Y. 24849 (PE), Dai T.L. 106825 (PE), Dai T.L. 106639 (PE), Dai T.L. 103893 (PE), Jiang S. 7067 (PE), Zhou H.C. 9000 (PE), Zhou H.C. 1064 (PE), Wang M.X. 07670 (PE), Song Z.P. 39386 (PE), Xiong & Zhou 90206 (PE), Jiang X.L. 11041 (PE), Jiang X.L. 11041 (PE), Jiang X.L. 11041 (PE), Jiang X.L. 11041 (PE), T.Naito et al. 342 (PE), Fang W.P. 0038 (PE), Fang W.P. 9091 (PE), Fang W.P. 38 (PE), T.T.Yu 776 (PE), Fang W.P. 9875 (PE), Jiang S. 10151 (PE), Jiang & Jin 00577 (PE), Diao Z.S. 624 (PE), Li P.Y. 5259 (PE), Li P.Y. 6739 (PE), Li P.Y. 5996 (PE), Jiang S. 7067 (PE), Chen & Lang 02266 (PE), Xu J.M. 2 (PE), Yi T.P. 75142 (PE), Fang M.Y. 24997 (PE), C.Pei 7720 (PE), Pei 8063 (PE), y.p.sun 1388 (PE), T.T.Yu 1858 (PE), Hopkinson 208 (PE), T.T.Yu 776 (PE), Harry Smith 13377 (PE), T.P.Wang 7949 (PE), Anonymous 573 (PE), Liu Z.Y. 181499 (PE), Sanxia Team 0351 (PE), Sanxia Team 0351 (PE), Liu Z.Y. 180669 (PE), Shandong Team 418 (PE), Sanxia Team 1321 (PE), Sanxia Team 1321 (PE), Wu Y.Z. ch0810001 (PE), Qi X.P. 4588 (PE), Bashan Team 2763 (PE), He L.D. 6735 (PE), Zhang X.C. 6726 (PE), Zhang et al. 836 (PE), Wan et al. FSB-781A (PE)
Pyrrosia piloselloides (L.) M.G. Price	Schneider et al. 5535 (PE), Zhang X.C. 5549 (PE), Wei & Wei wxp060 (PE), A.H.G.Alston 15962 (PE)

Pyrrosia polydactyla (Hance) Ching	Lu P.F. 21867 (PE), Liu Y.C. qy1 (PE), Lv P.F. 21430 (PE), T.Murakami et al. 182 (PE), Chen et al. 9846 (PE), Shen R.Y. 1211 (PE), W.Hancock 33 (PE), Y.Yamamoto s.n. (PE), Anonymous 1390 (PE), Tanaka 140 (PE), Tanaka 140 (PE), Y.Tateishi et al. 18116 (PE), Y.Tateishi et al. 16142 (PE), Y.Tateishi et al. 16142 (PE), H.Ohashi et al. 12974 (PE), D.Z.Fu & X.C.Zhang 96026 (PE), Huang et al. 4107 (PE), S.Suzuki 9336 (PE), Y.Yamamoto s.n. (PE), Y.Yamamoto s.n. (PE), Lv B.F. 21867 (PE)
Pyrrosia porosa (C. Presl) Hovenkamp	Wei X.P. wys10 (IMD), Zhang et al. 6223 (PE), Shui et al. 80900 (PE), FLPH Tibet Expedition 12-0959 (PE), Chen Y.S. 10-3399 (PE), C.E.D. 2556 (PE), QTP Team 6310 (PE), QTP Team 7732 (PE), QTP Plants Team 5192 (PE), QTP Plants Team 5185 (PE), QTP Plants Team 4659 (PE), Xie & Kong 396124 (PE), Guan & Wang 3252 (PE), Ying J.S. 4362 (PE), Ying J.S. 4362 (PE), Ying J.S. 4667 (PE), Ying J.S. 4667 (PE), Kong X.X. 5602 (PE), Zhao & Tan 123461 (PE), Zhao & Tan 123461 (PE), Zhao & Tan 123461 (PE), Zhao et al. 150717 (PE), QTP Plants Team 3790 (PE), Anonymous 75-1873 (PE), Lau S.K. 5478 (PE), S.P.Khullar 48 (PE), S.P.Khullar 57 (PE), S.P.Khullar 47 (PE), S.P.Khullar 46 (PE), Robert L.Fleming 1758 (PE), Robert L.Fleming 1262 (PE), E.B.Copeland 158 (PE), Lang et al. 1699 (PE), Lang et al. 1699 (PE), QTP Team 11356 (PE), QTP Team 11356 (PE), Guan & Wang 3252 (PE), Guan & Wang 2544 (PE), Zheng Z.S. 790 (PE), Xing & Lang 1656 (PE), Xing & Lang 0617 (PE), Kong X.X. 3648 (PE), QTP Plants Team 11609 (PE), Kong X.X. 2468 (PE), Kong X.X. 1756 (PE), Yang G.H. 57187 (PE), Li X. 71950 (PE), Guan Z.T. 6303 (PE), Li & Zhou 72641 (PE), Song Z.P. 39371 (PE), Jiang X.L. 35038 (PE), Hu & He 11972 (PE), Hu & He 11972 (PE), Pei 8076 (PE), Pei 8076 (PE), T.P.Wang 9429 (PE), Wan W.P. 3324 (PE), Wan W.P. 3324 (PE), Wan W.P. 3490 (PE), Wan W.P. 9533 (PE), Wan W.P. 9533 (PE), Wan W.P. 8824 (PE), Anonymous 4819 (PE), Wang P.S. 1092 (PE), Wang P.S. 1043 (PE), Wang P.S. s.n. (PE), Cao Z.Y. 0497 (PE), Cao Z.Y. 0497 (PE), Libo Team 1410 (PE), Libo Team 1410 (PE), Zhang & Zhang 7672 (PE), Anshun Team 646 (PE), Anshun Team 646 (PE), Zhang & Zhang 6419 (PE), Zhang & Zhang 5223 (PE), Zhang & Zhang 4023 (PE), Zhang & Zhang 813 (PE), Zhang & Zhang 6109 (PE), Zhang & Zhang 3051 (PE), Zhang & Zhang 3363 (PE), Cavalerie 146 (PE), Y.Tsiang 7083 (PE), Zhou N.S. 285 (PE), He J. 2231 (PE), H.Migo s.n. (PE), H.Shimada s.n. (PE), G.Masamune 1141 (PE), G.Masamune 1141 (PE), C.E.D. 7202 (PE), Liu X.X. 28502 (PE), Qin R.C. 7172 (PE), Qin R.C. 7172 (PE), China-Russia Team 5853 (PE), China-Russia Team 5516 (PE), China-Russia Team 5853 (PE), China-Russia Team 5853 (PE), China-Russia Team 5853 (PE), China-

	Russia Team 5853 (PE), China-Russia Team 7996 (PE), China-Russia Team 5853 (PE), China-Russia Team 1024 (PE), China-Russia Team 5516 (PE), China-Russia Team 5853 (PE), China-Russia Team 561 (PE), China-Russia Team 5339 (PE), Zhang & Dong 1405 (PE), C.W.Wang 74472 (PE), C.W.Wang 63009 (PE), C.W.Wang 63032 (PE), C.W.Wang 62930 (PE), C.W.Wang 62765 (PE), C.W.Wang 62765 (PE), H.T.Tsai 57518 (PE), H.T.Tsai 57283 (PE), H.T.Tsai 57283 (PE), H.T.Tsai 51425 (PE), H.T.Tsai 53654 (PE), 53066 (PE), H.T.Tsai 55386 (PE), H.T.Tsai 60126 (PE), H.T.Tsai 60773 (PE), Liu S.E. 13612 (PE), Liu S.E. 13462 (PE), Liu S.E. 14730 (PE), Liu S.E. 13201 (PE), K.M.Feng 12637 (PE), China-Russia Team 5077 (PE), Qin R.C. s.n. (PE), Anonymous 0048 (PE), X.C.Zhang C.S. 1104 (PE), Zhang X.C. 2796 (PE), Wang Z.R. 491 (PE), Wang Z.R. 744 (PE), Wang Z.R. 744 (PE), Dong Q.Z. 603 (PE), Dong Q.Z. 812 (PE), QTP Team 737 (PE), QTP Team 872 (PE), QTP Team 872 (PE), QTP Team 404 (PE), QTP Team 404 (PE), Liu S.E. 19375 (PE), Liu S.E. 018975 (PE), Xv W.X. 65 (PE), Qiu B.Y. 52644 (PE), Qiu B.Y. 52644 (PE), Yin W.Q. 778 (PE), Zhu & Wu 02648 (PE), Qiu B.Y. 57888 (PE), Wang H.C. 3418 (PE), Wang & Liu 84173 (PE), Anonymous 6045 (PE), Anonymous 28502 (PE), E.H.Wilson 5328 (PE), Liu S.E. 22163 (PE), Jiang Y. 16014 (PE), Qiu B.Y. 54188 (PE), Yin W.Q. 304 (PE), Yin W.Q. 304 (PE), Xin J.S. 355 (PE), Shui Y.M. 001756 (PE), G.Forrest 11766 (PE), QTP Team 5591 (PE), QTP Team 5591 (PE), QTP Team 5591 (PE), QTP Team 6821 (PE), QTP Team 6821 (PE), QTP Team 6310 (PE), QTP Plants Team 4478 (PE), QTP Plants Team 4478 (PE), QTP Plants Team 4478 (PE), QTP Plants Team 4549 (PE), Sun H.F. s.n. (PE), Sun H.F. s.n. (PE), H.F.Sun s.n. (PE), E.B.B. 158 (PE), F.Ludlow et al. 6682 (PE), A.Saulreie 434 (PE), Anonymous s.n. (PE), Anonymous s.n. (PE), Wu et al. 17 (PE), X.C.Zhang 2434 (PE), Wang et al. 3969 (PE), Wang et al. 4104 (PE), Wang et al. 81061 (PE), Wang et al. 80418 (PE), X.P.Qi 4543 (PE), Qi X.P. Q103 (PE), Shui Y.M. 80900 (PE), Qi X.P. Q059 (PE), Qi X.P. Q012 (PE), Shui Y.M. 80976 (PE), FLPH Tibet Expedition STET0082 (PE), FLPH Tibet Expedition 12-0959 (PE), FLPH Tibet Expedition 12-0959 (PE), FLPH Tibet Expedition 12-1912 (PE)
Pyrrosia rasamalae (Racib.) K.H. Shing	Wei et al. 3019 (PE), Zhang X.C. 8018-2 (PE), Sun H.F. 7014 (PE)
Pyrrosia rupestris Ching	Chinnock R.J. 8370 (PE), Constable E.F. 4141 (PE)
Pyrrosia schimperiana (Mett. ex Kuhn) Alston	Liu et al. 27233 (PE), Liu et al. 27327 (PE), Liu et al. 27272 (PE), Kornas J. 3573 (PE)

Pyrrosia serpens (G. Forster) Ching	Tindale M. et al. 7131a (PE)
Pyrrosia sheareri (Baker) Ching	Three Gorges Coll. Team 1800 (PE), Wei X.P. 2016041502 (PE), Liu H.M. a217 (PE), Tateishi et al. 21158 (PE), Wei R.wr0117 (PE), Yue J.S. 3370 (PE), F.H.Chen 2097 (PE), F.H.Chen 3580 (PE), F.H.Chen 5246 (PE), F.H.Chen 1259 (PE), Lu J.Q. 浙农 0129 (PE), Anonymous 10215 (PE), Qiu & Wu 6130 (PE), Deng et al. 4497 (PE), He X.Y. 20995 (PE), Anonymous 30650 (PE), He X.Y. 28806 (PE), Anonymous 29058 (PE), Zhang S.R. 6631 (PE), Chen G.R. 1607 (PE), Qiu P.X. 3892 (PE), Qiu P.X. 86 (PE), He X.Y. 22699 (PE), Anonymous 29846 (PE), Zhang S.R. 3983 (PE), C.Y.Chiao 1506 (PE), Wang R.Z. W454 (PE), Xiong Y.G. 05757 (PE), Xiong Y.G. 05542 (PE), Wang M.J. 0467 (PE), 236 Team 1161 (PE), Qiu P.X. 02463 (PE), Anonymous 0176 (PE), Yue J.S. 1789 (PE), Deng & Lang 930044 (PE), Wang M.J. 3580 (PE), Anonymous 63 (PE), Anonymous 2282 (PE), Anonymous 0176 (PE), Yue et al. 7210 (PE), K.K.Tsoong 4514 (PE), K.K.Tsoong 4514 (PE), Anonymous 4514 (PE), Anonymous 4514 (PE), K.K.Tsoong 4872 (PE), T.N.Liou et al. 2097 (PE), He X.Y. 21291 (PE), He X.Y. 21021 (PE), Zhejiang Team 26951 (PE), Zhejiang Team 29020 (PE), Zhejiang Team 28214 (PE), Zhejiang Team 29515 (PE), Wang J.X. 1578 (PE), Anonymous 0177 (PE), T.N.Liou et al. 592 (PE), Anonymous 2011 (PE), Zhang S.R. 3983 (PE), Anonymous 31217 (PE), Anonymous 30581 (PE), Anonymous 31366 (PE), He X.Y. 26501 (PE), 236Team 1161 (PE), Wang et al. 2278 (PE), Jiangxi Team 262 (PE), Jiangxi Team 262 (PE), Jiangxi Team 2853 (PE), Jiangxi Team 1050 (PE), Jiangxi Team 1050 (PE), Nie & Chen 07865 (PE), Yue J.S. 5368 (PE), Lai S.K. 02743 (PE), Lai S.K. 02890 (PE), Anonymous 6116 (PE), Anonymous 6011 (PE), Hu Q.M. 4736 (PE), Yue et al. 3370 (PE), Yue et al. 2488 (PE), Xiong J. 02971 (PE), Anonymous 4991 (PE), Li & Cheng 849 (PE), Li & Cheng 849 (PE), Anonymous 4196 (PE), Yue et al. 2030 (PE), Yue et al. 4541 (PE), Nie Min Xiang 92348 (PE), Anonymous 04009 (PE), Cheng & Yang 730384 (PE), S.H.Hsiung 294 (PE), Li Z.T. 1704 (PE), Nankai University s.n. (PE), Liu & He 15229 (PE), Xiangxi Team 0301 (PE), Zhang Z.G. 40 (PE), Anonymous 30 (PE), Liu L.H. 1785 (PE), Anonymous 80 (PE), Anonymous 80 (PE), Xi X.Y. 0019 (PE), Anonymous 1441 (PE), Anonymous 126 (PE), Anonymous 322 (PE), Anonymous 974 (PE), Luo L.B. 783 (PE), Y.Liu & Father 00293 (PE), Liu Q.X. 39 (PE), Anonymous 4514 (PE), Li X.G. 202924 (PE), Liu & He 015982 (PE), Tan P.X. 61910 (PE), Li X.G. 204252 (PE), Li & Wan 750198 (PE), H.F.Chow s.n. (PE), Shennongjia Team 30185 (PE), Fu & Zhang 1354 (PE), Fu & Zhang 1354 (PE), Fu & Zhang 1354 (PE), Fu & Zhang 1959 (PE), Fu & Zhang 1959

	(PE), Fu & Zhang 1829 (PE), Fu & Zhang 1829 (PE), Fu & Zhang 1829 (PE), Fu & Zhang 1829 (PE), Li H.J. 5468 (PE), Li H.J. 8691 (PE), Li H.J. 4009 (PE), Li H.J. 3430 (PE), Li H.J. 2482 (PE), Dai & Qian 鄂 1268 (PE), Dai & Qian 鄂 53 (PE), Zhou H.C. 100 (PE), Zhou H.C. 100 (PE), W.C.Cheng & C.T.Hwa 750 (PE), B.Bartholomew et al. 1925 (PE), B.Bartholomew et al. 2055 (PE), Li H.J. 6019 (PE), Li H.J. 912 (PE), Li H.J. 6010 (PE), Peng F.S. 106 (PE), Peng F.S. 8643 (PE), Li H.J. 8689 (PE), Li H.J. 8047 (PE), Li H.J. 4427 (PE), Li H.J. 6591 (PE), Li H.J. 5841 (PE), Li H.J. 9113 (PE), Li H.J. 5108 (PE), Li H.J. 8272 (PE), Fang M.Y. 24485 (PE), Anonymous s.n. (PE), Chuan J.Z. 0076 (PE), Chuan J.Z. 0076 (PE), Jiang S. 07597 (PE), Liu Z.Y. 4032 (PE), Liu Z.Y. 3857 (PE), Zhou et al. 107996 (PE), Fang M.Y. 24137 (PE), Yang G.H. 65600 (PE), Li G.F. 65042 (PE), Li G.F. 64846 (PE), Li G.F. 63371 (PE), Guan Z.T. 9487 (PE), Zhou & Li 111031 (PE), Zhou & Li 107998 (PE), Zhou & Li 111571 (PE), Zhou & Li 107642 (PE), Zhou & Li 107996 (PE), Guan et al. 245 (PE), Guan et al. 245 (PE), Wang F.Z. 10521 (PE), Lin L.Y. 1534 (PE), Sun X.L. 5659 (PE), Yu D.J. 3524 (PE), Anonymous s.n. (PE), Li M.S. 317 (PE), Deng & Zhou 0350 (PE), Guan Z.T. 8365 (PE), Dai Z.T. 102682 (PE), Zhou H.F. 26047 (PE), Li G.F. 62926 (PE), Li G.F. 63128 (PE), Li G.F. 62319 (PE), Li G.F. 61100 (PE), Li G.F. 61352 (PE), Li G.F. 63257 (PE), Li G.F. 62273 (PE), Li G.F. 61069 (PE), Dai T.L. 107121 (PE), Zhou & Li 111293 (PE), Zhang Z.R. 25123 (PE), Zhang & Shi 544 (PE), Zhang & Shi 544 (PE), Anonymous 49 (PE), X.C.Zhang et al. 2610 (PE), Jinfoshan Team 1775 (PE), Guan Z.T. 9487 (PE), Guan Z.T. 7900 (PE), Xiong & Zhou 90319 (PE), Xiong & Zhou 90281 (PE), Anonymous 4514 (PE), Anonymous D244 (PE), Zhang S.F. 175 (PE), Zhang S.F. 231 (PE), Qin R.C. 10340 (PE), Fang W.P. 1814 (PE), T.P.Wang 10544 (PE), T.P.Wang 10346 (PE), T.T.Yu 4353 (PE), Fang W.P. 5729 (PE), Fang W.P. 4539 (PE), Chen Zhiduan et al. 960957 (PE), Anonymous 3067 (PE), Anonymous 92001 (PE), Anonymous 3490 (PE), C.Pei 7265 (PE), C.Pei 7265 (PE), N.L.Chu 931 (PE), Dai T.L. 104795 (PE), Chuan J.Z. 0331 (PE), Anonymous 5659 (PE), Fang W.P. 5840 (PE), Fang W.P. 1066 (PE), Fang W.P. 1066 (PE), Qiannan Team 2132 (PE), Qiannan Team 2132 (PE), Qiannan Team 03154 (PE), Qiannan Team 03154 (PE), Anshun Team 1064 (PE), Anshun Team 1064 (PE), Chuanqian Team 1745 (PE), Chuanqian Team 1537 (PE), Zhang et al. 402066 (PE), Zhang et al. 401863 (PE), Jian et al. 31611 (PE), Zhang et al. 401575 (PE), Jian et al. 30574 (PE), Jian et al. 30957 (PE), Chuanqian Team 1745 (PE), Chuanqian Team 1627 (PE), Chuanqian Team 1685 (PE), Chuanqian Team 1235 (PE), Cao Z.Y. 0353 (PE), Cao Z.Y. 0353 (PE), Cao Z.Y. 0286 (PE), Cao

	Z.Y. 0286 (PE), Anshun Team 1536 (PE), Anshun Team 1536 (PE), Qiannan Team 02417 (PE), Qiannan Team 02417 (PE), Anshun Team 488 (PE), Anshun Team 488 (PE), Bijie Team 991 (PE), Bijie Team 991 (PE), Bijie Team 991 (PE), Qianbei Team 0074 (PE), Qianbei Team 0074 (PE), Qianbei Team 2119 (PE), Qianbei Team 2119 (PE), Qiannan Team 01013 (PE), Qiannan Team 01013 (PE), Zhang & Zhang 01002 (PE), Qiannan Team 01002 (PE), Qianbei Team 2861 (PE), Qianbei Team 2861 (PE), Qiannan Team 03533 (PE), Qiannan Team 03533 (PE), Anshun Team 1775 (PE), Zhang & Zhang 4619 (PE), Libo Team 1479 (PE), Libo Team 2128 (PE), Zhang & Zhang 946 (PE), Bijie Team 514 (PE), Zhang & Zhang 7371 (PE), Libo Team 2128 (PE), Yu P.H. 703 (PE), Wang P.S. 75787 (PE), Anonymous 1149 (PE), Jian et al. 50669 (PE), Anonymous 3490 (PE), Tu & Sun 3067 (PE), Tu & Sun 3067 (PE), Y.Tsiang 5084 (PE), C.Y.Chiao et al. 422 (PE), C.Y.Chiao et al. 422 (PE), C.Y.Chiao et al. 422 (PE), C.Y.Chiao et al. 56 (PE), C.Y.Chiao et al. 56 (PE), B.Bartholomew et al. 1840 (PE), B.Bartholomew et al. 1840 (PE), B.Bartholomew et al. 1560 (PE), Zhang Q.Q. 852 (PE), Anonymous 5399 (PE), Anonymous 5422 (PE), Li M.S. 764 (PE), LI M.S. 764 (PE), Li Z.Y. 10699 (PE), Lin L.G. s.n. (PE), Anonymous 342 (PE), Anonymous 342 (PE), Wang & Huang 3407 (PE), Qiu P.X. 1833 (PE), Qiu P.X. 1833 (PE), Qiu P.X. 1833 (PE), Jian et al. 400462 (PE), Jian et al. 400631 (PE), Anonymous 178 (PE), D.Z.Fu & X.C.Zhang 96022 (PE), S.Suzuki s.n. (PE), S.Tanaka s.n. (PE), Huang et al. 4084 (PE), Gao X.P. 53747 (PE), Deng L. 5641 (PE), Huang Z. 44195 (PE), Huang Z. 44282 (PE), Deng L. 7221 (PE), S.P.Kwak 80028 (PE), Tsang W.T. 20908 (PE), Anonymous 30548 (PE), Anonymous 20548 (PE), S.P.Ko 55882 (PE), Qiu P.X. 4777 (PE), Li & Chen 600467 (PE), Chen Z.Z. 51086 (PE), Deng X.F. 263 (PE), Anonymous 305 (PE), Lv Q.H. 3634 (PE), Liu X.Q. 28670 (PE), Anonymous 438 (PE), Anonymous 438 (PE), Anonymous 52811 (PE), Deng S.W. 90276 (PE), L.Tang 230 (PE), Tsang W.T. 20908 (PE), Tsang W.T. 20908 (PE), Zhu W.M. 4831 (PE), Liu S.E. 018484 (PE), Liu S.E. 018484 (PE), Liu Z.Y. 181407 (PE), Liu Z.Y. 180326 (PE), Liu Z.Y. 180273 (PE), Longxishan Team 869 (PE), Longxishan Team 01000 (PE), Longxishan Team 0998 (PE), Bashan Team 1504 (PE), Bashan Team 0976 (PE), Bashan Team 2359 (PE), Bashan Team 2227 (PE), FLPH Tibet Expedition 1022 (PE), Tan C.M. 95498 (PE), Sanxia Team 1987 (PE)
Pyrrosia stenophylla (Bedd.) Ching	Li Z.Y.l1740 (PE), Zhang X.C. 3959 (PE), Zhang X.C. 3992 (PE), STET0082 (PE), Jin X.H. et al, ST1017 (PE), D.Z.Fu & X.C.Zhang 96014 (PE), Li & Cheng 05139 (PE), Robert L.Fleming 2157 (PE), QTP Team 9268 (PE),

	QTP Team 9268 (PE), QTP Team 8886 (PE), QTP Team 8886 (PE), QTP Team 8886 (PE), QTP Team 9491 (PE), Mao P.Y. 00471 (PE), T.T.Yu 20861 (PE), T.T.Yu 20861 (PE), T.T.Yu 20861 (PE), Zhenxi Team 11331 (PE), QTP Team 3560 (PE), QTP Team 3560 (PE), QTP Team 3560 (PE), Zhang & Wang 0340 (PE), Zhang & Wang 0488 (PE), Anonymous 75-1873 (PE), Zhang & Lang 861 (PE), Zhang & Lang 861 (PE), Zhang & Lang 864 (PE), Cheng & Li 03062 (PE), Cheng & Li 03062 (PE), Cheng & Li 03261 (PE), Cheng & Li 04081 (PE), Cheng & Li 03630 (PE), Zhang J.W. 0896 (PE), Zhang & Lang 772 (PE), Zhang & Lang 772 (PE), Anonymous 03454 (PE), Anonymous s.n. (PE), Li & Cheng 03261 (PE), Li & Cheng 03261 (PE), QTP Team 1420 (PE), QTP Team 1420 (PE), QTP Team 03109 (PE), QTP Team 03109 (PE), QTP Team 03109 (PE), QTP Team 809 (PE), QTP Team 809 (PE), QTP Team 5038 (PE), QTP Team 5038 (PE), QTP Team 3560 (PE), QTP Team 3560 (PE), Tibet Team 1518 (PE), Tibet Team 1518 (PE), Tibet Team 1518 (PE), Tibet Team 713 (PE), Tibet Team 713 (PE), Li & Cheng 03454 (PE), Li & Cheng 03454 (PE), Li & Cheng 02127 (PE), Li & Cheng 02534 (PE), Li & Cheng 01905 (PE), Li & Cheng 01905 (PE), Li & Cheng 01905 (PE), Li & Cheng 02388 (PE), Li & Cheng 02388 (PE), , Li & Cheng 04081 (PE), Li & Cheng 03630 (PE), Chen W.L. 14365 (PE), Ying & Hong 650774 (PE), Ying & Hong 650774 (PE), Ying & Hong 650774 (PE), Ying & Hong 650774 (PE), Ying & Hong 650774 (PE), T.Naito et al. 991 (PE), T.Naito et al. 991 (PE), T.Naito et al. 910 (PE), T.Naito et al. 910 (PE), T.Naito et al. 880 (PE), T.Naito et al. 999 (PE), S.P.Khullar 38 (PE), G.Forrest 25022 (PE), Hideo Tabata et al. 0222 (PE), Hideo Tabata et al. 0325 (PE), X.C.Zhang 3992 (PE), X.C.Zhang 4014 (PE), X.C.Zhang 4014 (PE), X.C.Zhang & L.Wang 4768 (PE), X.C.Zhang & L.Wang 4895 (PE), X.C.Zhang & L.Wang 4741 (PE), X.C.Zhang & L.Wang 4741 (PE), Zhang X.C. 4048 (PE), Zhang X.C. 3959 (PE), FLPH Tibet Expedition 12-1912 (PE), Chen et al. 433 (PE)
Pyrrosia stigmosa (Sw.) Ching	Sun H.F. 79 (PE), Sun H.F. s.n. (PE), Zhang & Dong 1410 (PE), QTP Team 4169 (PE), H.F.Sun 79 (PE), F.M.Jarrett et al. 765 (PE)
Pyrrosia subfurfuracea (Hook.) Ching	Wei & Wei wxp185 (PE), Li Z.Y.1925 (PE), Liu & Zhang 7760 (PE), Cheng S.Z. 04622 (PE), Qin H.N. 933 (PE), Wang Z.R. 490 (PE), Wang Z.R. 797 (PE), Zhang et al 1092 (PE), Mao P.Y. 00470 (PE), Zhu W.M. 11533 (PE), K.M.Feng 8672 (PE), C.W.Wang 67587 (PE), T.T.Yu 19960 (PE), Cheng & Li 02250 (PE), Cheng & Li 01708 (PE), Cheng & Li 02375 (PE), Cheng & Li 01531 (PE), Chen W.L. 14381 (PE), Anonymous 2372 (PE),

	Anonymous Q1(1) (PE), Wang Z.R. 490 (PE), Cheng & Li 04622 (PE), Cheng & Li 01780 (PE), Liu H.M. GX043 (PE), FLPH Tibet Expedition STET1925 (PE)
Pyrrosia tonkinensis (Giesenh.) Ching	Wei & Wei wxp058 (PE), Li Z.Y. lzy05 (IMD), Guo et al. 7106 (PE), Guo et al. 7233 (PE), Zhang et al. 1568 (PE), Zhang X.C. 5646 (PE), Qi X.P. q055 (PE), Jiang R.H. 294 (PE), Wang P.S. 1918 (PE), Hou X.Y. 1807 (PE), Zhang & Zhang 199 (PE), Zhang & Zhang 2314 (PE), Zhang & Zhang 3518 (PE), Zhang & Zhang 4964 (PE), Zhang & Zhang 3145 (PE), Wang & Wang C746 (PE), Y.Tsiang 9202 (PE), Y.Tsiang 9415 (PE), Liang K. 69008 (PE), Zhang X.C. 1140 (PE), X.C.Zhang C.S. 1113 (PE), X.C.Zhang 1089 (PE), Qiu P.X. 4969 (PE), Qiu P.X. 4909 (PE), Zhou & Li 1255 (PE), Li T.Z. 602815 (PE), Lv Q.H. 2623 (PE), Li Z.T. 601167 (PE), Fang & Qin 31334 (PE), Qin R.C. 5354 (PE), China-Russia Team 7025 (PE), China-Russia Team 5499 (PE), China-Russia Team 5544 (PE), China-Russia Team 857 (PE), China-Russia Team 599 (PE), China-Russia Team 3310 (PE)
Pyrrosia transmorrisonensis (Hayata) Ching	Liu Y. C. ys1 (PE)

Supplementary Table S4. Morphological character matrix. (a)Frond: 1. monomorphic, 2. moderately dimorphic, 3. dimorphic; (b)Rhizome/Phyllopodia: 1. short/contiguous, 2. long-creeping/separate; (c)Scale: 1. peltate, 2. pseudopeltate, 3. basifixed; (d)Scale margin: 1. mostly ciliate-dentate, 2. with long, curly cilia, 3. entire; (e)Stomata: 1. polocytic, 2. pericytic; (f)Venation: 1. campyloneuroid, 2. pseudo-drynarioid, 3. drynarioid; (g)Sori arrangement: 1. sori small, several to many in each areole, pseudo-acrostichoid when ripe, 2. sori large, two to three in each areole, 3. sori in a single row, 4. sori confluent in a coenosorus; (h)Indumenta: 1. monomorphic, boat-shaped or lanceolate rays, 2. monomorphic, boat-shaped + wooly rays, 3. dimorphic, boat-shaped and wooly rays, 4. monomorphic, acicular rays, dimorphic, acicular and wooly rays; (i)Epispore ornamentation: 1. sparsely granulose and small irregular ridges, 2. longitudinal ridges and coarsely verrucate, 3. bisculpate, granulate + warty or spinelike protuberances, 4. longitudinal ridges, 5. finely granulose, 6. smooth; (j)Hydathodes: 1. distinct, 2. absent; (k)Sunken depths of stomata: 1. superficial, 2. slightly sunken, 3. sunken; (l)Sunken depths of sori: 1. superfical, 2. sunken.

Species	(a)	(b)	(c)	(d)	(e)	(f)	(g)	(h)	(i)	(j)	(k)	(l)
Pyrrosia abbreviata (Zoll. & Moritzi) Hovenkamp	2	2	1	2	2	2	1	1	2	1	3	1
Pyrrosia adnascens (Sw.) Ching	3	2	1	1	2	1	1	1	3	2	3	2
Pyrrosia angustata (Sw.) Ching	3	2	1	3	2	2	3	5	4	2	3	1
Pyrrosia angustissima (Giesenh. ex Diels) Tagawa & K. Iwats.	1	2	1	1	2	3	4	5	1	1	2	1
Pyrrosia assimilis (Baker) Ching	1	1	1	1	2	1	1	4	5	1	2	1
Pyrrosia bonii (Christ ex Giesenh.) Ching	1	1	2	1	2	2	1	5	5	1	2	1
Pyrrosia calvata (Baker) Ching	1	1	3	1	2	1	1	5	5	1	2	1
Pyrrosia caudifrons Ching, Boufford & K.H. Shing	2	2	1	2	2	1	1	1	1	2	3	1
Pyrrosia christii (Giesenh.) Ching	2	2	1	1	2	1	1	3	1	1	3	1
Pyrrosia costata (Wall. ex C. Presl) Tagawa & K. Iwats.	1	1	3	3	2	2	1	3	6	1	1	1

Pyrrosia davidii (Giesenh. ex Diels) Ching	1	1	1	1	2	1	1	5	5	1	2	1
Pyrrosia drakeana (Franch.) Ching	1	1	2	1	2	1	1	5	5	1	1	1
Pyrrosia eberhardtii (Christ) Ching	2	2	1	2	2	1	1	2	1	2	3	1
*Pyrrosia eleagnifoli*a (Bory) Hovenkamp	3	2	1	1	2	2	2	1	3	2	3	2
Pyrrosia ensata Ching ex K.H. Shing	3	2	1	3	2	1	1	3	1	1	2	1
Pyrrosia fengiana Ching	1	1	2	1	2	2	1	5	5	1	1	1
Pyrrosia flocculosa (D. Don) Ching	1	1	2	1	2	1	1	5	5	1	2	1
Pyrrosia foveolata (Alston) C.V. Morton	2	2	1	1	2	1	2	1	3	1	3	2
Pyrrosia gralla (Giesenh.) Ching	1	1	1	1	2	1	1	4	5	1	2	1
Pyrrosia hastata (Thunb. ex Houtt.) Ching	1	1	1	1	2	2	1	1	5	1	1	1
Pyrrosia heteractis (Mett. ex Kuhn) Ching	2	2	1	2	2	1	1	2	1	2	3	1
Pyrrosia kinabaluensis Hovenkamp	2	2	1	1	2	3	1	5	2	2	3	1
Pyrrosia laevis (J. Sm. ex Bedd.) Ching	1	2	1	1	2	1	1	1	1	1	2	1
Pyrrosia lanceolata (L.) Farw.	1	2	1	1	2	1	1	1	3	2	3	2
Pyrrosia linearifolia (Hook.) Ching	1	1	1	1	2	1	3	5	5	1	2	1
Pyrrosia lingua (Thunb.) Farw.	2	2	1	2	2	1	1	1	1	1	3	1
Pyrrosia longifolia (Burm. f.) C.V. Morton	1	2	1	3	2	1	1	1	3	2	3	2
Pyrrosia mannii (Giesenh.) Ching	1	1	2	1	1	2	1	5	5	1	1	1
Pyrrosia martini (Christ) Ching	2	2	1	2	2	1	1	1	1	1	3	1
Pyrrosia niphoboloides (Luerss.) M.G. Price	3	2	1	1	2	3	4	4	3	2	1	1
Pyrrosia nuda (Giesenh.) Ching	2	2	1	1	2	1	1	1	3	2	3	2
Pyrrosia nudicaulis Ching	1	1	1	1	2	1	1	5	5	1	2	1
Pyrrosia nummulariifolia (Sw.) Ching	3	2	1	1	2	3	1	5	2	2	3	1
Pyrrosia oblonga Ching	2	2	1	2	2	1	1	1	1	1	3	1
Pyrrosia penangiana (Hook.) Holtt.	1	1	2	1	1	1	1	4	5	1	1	1

Pyrrosia petiolosa (Christ) Ching	2	2	1	1	2	1	1	1	1	1	3	1
Pyrrosia piloselloides (L.) M.G. Price	3	2	1	1	2	3	4	1	3	2	3	1
Pyrrosia polydactyla (Hance) Ching	1	1	1	1	2	2	1	1	5	1	2	1
Pyrrosia porosa (C. Presl) Hovenkamp	1	1	1	1	2	1	1	5	5	1	2	1
Pyrrosia rasamalae (Racib.) K.H. Shing	1	2	1	3	2	1	1	5	2	2	3	1
Pyrrosia rupestris Ching	3	2	1	1	2	1	2	1	3	1	3	2
Pyrrosia samarensis (C. Presl) Ching	3	2	1	3	2	2	4	5	4	2	3	1
Pyrrosia schimperiana (Mett. ex Kuhn) Alston	1	1	2	1	1	3	1	1	5	1	2	1
Pyrrosia serpens (G. Forster) Ching	2	2	1	1	2	1	2	1	3	1	3	2
Pyrrosia sheareri (Baker) Ching	1	1	2	1	2	2	1	1	5	1	1	1
Pyrrosia stenophylla (Bedd.) Ching	1	1	1	1	2	1	1	5	5	1	2	1
Pyrrosia stigmosa (Sw.) Ching	1	1	3	1	2	1	1	2	6	1	1	1
Pyrrosia subfurfuracea (Hook.) Ching	1	1	2	1	2	1	1	5	5	1	2	1
Pyrrosia subtruncata Ching	1	1	2	1	2	2	1	5	5	1	2	1
Pyrrosia tonkinensis (Giesenh.) Ching	1	1	1	1	2	1	1	5	5	1	2	1
Pyrrosia transmorrisonensis (Hayata) Ching	1	1	1	1	2	1	1	5	5	1	2	1

Supplementary References

1. Nayar, B.K. & Chandra, S. Ferns of India, XV, *Pyrrosia* Mirbel. *Bull. Natl. Bot. Gard. Lucknow* **117**, 1–98 (1965).
2. Shing, K.H. A Reclassification of the Fern Genus *Pyrrosia*. *Am. Fern J.* **73**, 73–78 (1983).
3. Hovenkamp, P. H. *A Monograph of the Fern Genus Pyrrosia (Polypodiaceae)*. (E. J. Brill/Leiden University Press, 1986).
4. Yang, L.H. *A Systematic Study on the Fern Genus Pyrrosia Mirbel.* Ph. D thesis, (Yunnan University, 2012).
5. Zhou, X.M., *et al.* A plastid phylogeny and character evolution of the Old World fern genus *Pyrrosia* (Polypodiaceae) with the description of a new genus: *Hovenkampia* (Polypodiaceae), *Mol. Phylogenet. Evol.* **114**: 271–294 (2017).
6. Vasques D.T., Ebihara A. & Ito M. The felt fern genus *Pyrrosia* Mirbel (Polypodiaceae): a new subgeneric classification with a molecular phylogenetic analysis based on three plastid markers. *Acta Phytotax. Geobot.* **68** (2): 65–82 (2017).
